# Sentiment analysis in Bangla: A three-class dataset and comparative evaluation of machine learning, deep learning, and transformer approaches

**DOI:** 10.1371/journal.pone.0360952

**Published:** 2026-10-27

**Authors:** Md. Nahid Hasan, Jahanur Biswas, Antu Chowdhury, Md. Rakib Hossain, Mir Aminul Haque, Md. Muksit Ul Islam

**Affiliations:** 1 Department of Computer Science and Engineering, Dhaka International University, Dhaka, Bangladesh; 2 Department of Computer Science and Engineering, East West University, Dhaka, Bangladesh; University of Baghdad, IRAQ

## Abstract

Despite the increasing growth of sentiment analysis (SA) in several languages, Bangla is largely unexplored as a result of the low availability of annotated datasets, class imbalance, and challenging linguistic features such as inflectional morphology and code mixing. Existing work has concentrated mostly on limited or in-domain corpora, which leaves a void for general multi-domain sentiment resources based on real-world usage. The aim of the research work is to present such a balanced three-class sentiment dataset from Bangla social media text messages, with each post annotated by native speakers as positive, negative, or neutral. A robust preprocessing pipeline, including denoising, abbreviation expansion, and tokenization, was applied to improve data quality. Features were extracted using Count Vectorizer and Word2Vec embeddings, followed by the implementation of several machine learning (SVM, Random Forest, XGBoost) and deep learning (GRU, LSTM) models. In addition, fine-tuning was also applied to the BanglaBERT transformer model for comparison. The results show that BanglaBERT outperforms all other pretrained with approximately 80% accuracy, indicating good generalizability over sentiment classes. These observations support the success of transformer-based architectures trained on low-resource languages. The release of this dataset is expected to facilitate further advances in Bangla sentiment analysis and provide a scalable foundation for multilingual and cross-lingual NLP research.

## Introduction

Every keystroke, every tweet, every comment now holds the power to sway public opinion, inform key decisions, and even shape social movements. Among the multitude of online voices, Bangla, the language of more than 230 million speakers in Bangladesh and the Indian state of West Bengal, awaits exploration as a valuable information resource [1]. Although it is a historically and culturally important language, Bangla lags in sentiment analysis research for the most part because of limited annotated corpora and language-specific resources [2]. People use online discussion forums and social media to express their opinions on politics, social issues, and everyday matters, thereby shaping public discourse and influencing collective perceptions. These digital expressions inform consumer behavior and provide valuable insights that help decision-makers, institutions, and organizations understand public sentiment before taking action. Both researchers and analysts depend on working sentiment analysis tools in order to draw conclusions, but building those tools to work in Bangla is a fight against the hill.

Some factors exacerbate the problem of Bangla sentiment analysis. Some existing Bangla sentiment datasets are relatively small or domain-specific, and may exhibit class imbalance, which can pose challenges for developing robust machine learning and deep learning models [3]. Moreover, Bangla’s linguistic characteristics–-its rich inflectional morphology, long compound words (for example, “অর্থনৈতিকরাজনীতি” [economic politics]), and prevalent code‐mixing with English–-complicate tasks such as text normalization and feature extraction. Off‐the‐shelf tools developed for English often misinterpret Bangla text unless carefully adapted. Additionally, there is a lack of well‐established lexical resources, sentiment lexicons, and pretrained embeddings tailored specifically to Bangla. Although multilingual transformer models (such as mBERT or XLM‐R) offer some advantages, they generally do not match the performance of monolingual models trained on sizable in‐domain Bangla data [4]. Compounding these issues, most prior work has framed sentiment analysis as a binary problem (positive vs. negative), overlooking neutral sentiments that often dominate balanced news commentary and mixed-tone reviews.

To bridge this gap, this study focuses on a truly Bangla-centric resource: a newly created three-class sentiment dataset drawn directly from social media. Posts were manually compiled from platforms such as Facebook and X, ensuring that each entry reflects authentic, user-generated Bangla content. Ten native speakers then annotated every post with one of three sentiment labels–-positive, negative, or neutral–-capturing the full spectrum of sentiment nuances that binary frameworks often miss. By focusing on this dataset as a keystone contribution, a foundation is established upon which future Bangla sentiment research can reliably build. The preprocessing pipeline unfolds in three sequential steps: first, noncharacter symbols (emojis and punctuation) are removed to minimize noise; next, common shorthand notations are expanded (e.g., converting “ বি. দ্র.” to “ বিশেষ দ্রষ্টব্য”); finally, the text is tokenized. The word cloud in Fig 1 visualizes the most frequent tokens in our social-media corpus for Bangla (a) and English (b), with a font size proportional to the token frequency: in the Bangla cloud, the highest-frequency tokens are “ এই” (this), “ আর” (and/also), and “ না” (no), whereas the English cloud foregrounds “of”, “to”, and “the”.

**Fig 1:**
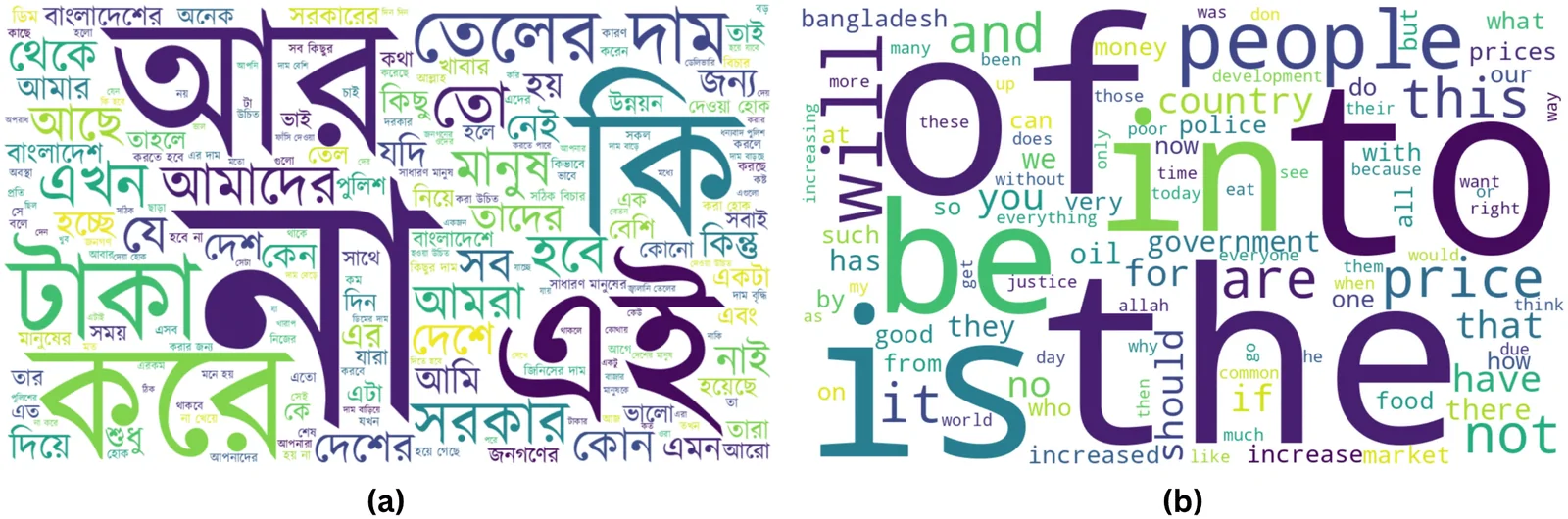
Word clouds of the most frequent social-media tokens in Bangla (a) and English (b).

To counteract imbalances across positive, negative, and neutral labels, the Synthetic Minority Over-Sampling Technique (SMOTE) was applied in a multiclass fashion [5], generating sufficient samples for underrepresented classes and yielding a balanced dataset.

Once the Bangla posts were balanced with SMOTE and cleaned (symbols removed, abbreviations expanded, and code-mixed text tokenized), the cleaned posts were converted into numerical representations in two ways. First, Count Vectorizer [6] was used to create a sparse matrix of word-frequency counts; this simple approach works well for machine learning algorithms such as SVM or Random Forest. Second, a Word2Vec model was trained on the Bangla corpus to generate 100-dimensional embeddings that capture semantic similarities among words (for example, “ রাজা” and “ রানী” result in similar vectors). These dense vectors serve as inputs for neural network models (GRU and LSTM). In addition, contextual embeddings were extracted from a pretrained BanglaBERT model to evaluate how a transformer-based representation performs on the same data.

Using these features, several classifiers were trained. For Count Vectorizer inputs, classical machine learning models (Decision Tree, Naïve Bayes, Support Vector Machine, Random Forest, XGBoost, and K-Nearest Neighbors) were tuned using Randomized SearchCV to identify the optimal hyperparameters. For Word2Vec embeddings, GRU and LSTM networks were constructed, followed by dropout and a dense output layer, and the learning rate and batch size were optimized through grid search on a validation split. In addition, the classification head of the BanglaBERT model was fine-tuned on the proposed dataset to compare its performance with that of other models. Although BanglaBERT achieved the best individual performance, overall consistency was further improved by combining the predictions of all models in a stacked ensemble, thereby enhancing the reliability of sentiment classification across positive, negative, and neutral classes.

By leveraging a variety of modeling approaches, The goal of this study is to support the development and refinement of sentiment analysis techniques tailored to the unique characteristics of the Bangla language. The stacked ensemble model uses predictions from all machine learning and deep learning models to provide the final prediction.

The key contributions of this study are listed below.

- **Developing a Bangla Sentiment Dataset with Three Classes:** To overcome the shortcomings of binary sentiment classification, a unique data set of Bangla social media posts with three sentiment labels, positive, negative, and neutral, annotated by native speakers, was created. The dataset can be found via the following link “https://data.mendeley.com/datasets/rh67mckhbh/2”.
- **Domain-Specific Preprocessing Pipeline:** Designed a preparation workflow specifically for Bangla that includes tokenization of code-mixed text, shorthand expansion, and symbol removal to provide high-quality input for modeling.
- **Balanced Dataset with SMOTE:** Addressing class imbalances via the Synthetic Minority Over-Sampling Technique (SMOTE), allowing for more reliable training across sentiment categories.
- **Multi-Model Classification and Stacked Ensemble:** A range of models, such as neural networks with Word2Vec embeddings, transformer-based BanglaBERT, and traditional machine learning with Count Vectorizer, are used to predict sentiment. In addition, a stacked ensemble was created utilizing a neural network and machine learning models to increase classification consistency and accuracy.

The remainder of this paper is organized as follows: the Literature Review section presents recent and relevant works in Bangla and multilingual sentiment analysis. The Methodology section describes the dataset collection, preprocessing, and model development procedures. The Results and Discussion section presents the model performance and compares the findings with existing approaches. The Conclusion section summarizes the study and outlines future research directions. Finally, the Acknowledgments section is provided at the end of the manuscript.

## Literature review

This section provides a comprehensive review of the existing body of research on sentiment analysis, with particular emphasis on Bangla-language studies. The main challenges, approaches, and limitations that motivated the development of the proposed sentiment analysis methodology tailored to Bangla are highlighted.

Ullah [7] proposed BCLSA, a concept-level sentiment analysis framework for Bangla that integrates syntactic, semantic, and affective knowledge. The CLSA-Bn algorithm models negation, modifiers, and concept polarity for interpretable classification, achieving up to 90.2% accuracy on 10,243 formal news articles and 12,084 informal social media comments, demonstrating efficiency suitable for real-time, low-resource deployment. Bhowmik et al. [8] proposed BN-GRAPH, a Bangla text sentiment analysis framework combining data augmentation, transformers, and graph neural networks. By leveraging multi-modal augmented data and graph-based feature engineering, the model effectively addresses class imbalance and captures contextual sentiment, achieving 89.57% accuracy and an F1-score of 0.8635 on a three-class sentiment dataset. To tackle multilingual product review analysis, Shahnaz Ipa et al. [9] developed BdSentiLLM, a system capable of classifying languages and performing sentiment analysis across English, Bangla, Banglish, and code-switched content. Using Llama-2, the model achieved the highest overall performance (F1 = 0.89), surpassing existing methods in handling mixed-language data. Rahman et al. [10] employed a new exhaustive Bangla language e-learning dataset, consisting of 6,268 YouTube remarks, to develop a method for sentiment analysis (SA). The dataset included 3,178 Bangla, 3,090 Romanized Bangla, and a combined set. Several models have been utilized, including the ML, DL, ensemble, hybrid, and transformer models. XLM-RoBERTa had the highest accuracy for the Bangla (89.46%) and combined (85.81%) datasets, but Artificial Neural Network (ANN) was the highest for the Romanized Bangla (89.59%). For the Bangla product SA, a system was preferred by Sunny et al. [11] who utilized various classification algorithms. The authors received 5016 Bangla sentiment data through social media pages and groups, as well as online purchasing sites such as Chaldal.com and Daraz. Their dataset is composed of 52.9% positive sentiments and 47.1% negative sentiments. They applied eight classifiers, which include Extra Tree, KNN, Logistic Regression (LR), Naïve Bayes (NB), Random Forest (RF), ANN, Support Vector Machine (SVM), and Multilayer Perceptron (MLP). They attained a maximum accuracy rate of 96.42% via the MLP. Hassan et al. [12], on the other hand, seem to have focused on Bangla conversation reviews via ML techniques. They gathered 1141 data points from the film and short film script as dialogue, in which there were 571 records of positive dialogue and 570 records of negative dialogue. After that, seven ML algorithms were applied, and 85.59% accuracy was achieved via SVM. Emotion understanding has recently explored diverse modalities beyond text. For instance, Deng et al. [13] proposed a prompt-tuning framework using CLIP for image emotion classification, generating instance-specific prompts that improve performance across six affective datasets. Similarly, Wang et al. [14] proposed MDKAT for multimodal emotion recognition, integrating modules for Cross-modal Feature Fusion, Adaptive Masked Self-Attention, Knowledge Aggregation, and Knowledge Transfer, achieving superior performance on CMU-MOSI and CMU-MOSEI datasets. Furthermore, Deng et al. [15] addressed the spread of negative emotions on social networks by framing it as a negative emotion intervention (NEI) problem. The study introduced NECM-RLDB, a reinforcement learning-based dynamic blocking approach that models spreaders’ capability, receivers’ sensitivity, and event popularity, outperforming traditional heuristic methods on both artificial and real-world datasets. A further study was executed by Mia et al. [16] who focused on Bengali Music Analysis from Lyrics with the help of several ML and NN classifiers. They created a novel dataset that included 2152 Bangla songs, collected from 6500 verses. BERT outperforms the other methods, scoring 65% accuracy. Khan et al. [17] used LSTM and Bi-LSTM models for Bangla sentiment analysis, as the language has diverse expressions and limited datasets, and for classifying text into positive, negative, and neutral sentiments. The LSTM model achieved 99% accuracy with F1-scores of 98%, showing the effectiveness of deep learning for this task. To solve the problem of emotion interpretation from lesser-spoken languages such as Bengali, considering the increased use of Banglish, Faisal et al. [18] presented a new dataset titled ‘Bengali & Banglish’ for detecting emotions in linguistically diverse contexts. They developed a new dataset with 80,098 texts. These emotions were subsequently classified into six categories. Afterward, they worked with three ML models, three DL models, and three Transformer Models. The transformer-based approaches, particularly BanglaBERT, achieved the best results in emotion classification with accuracies of 71.12% for Bengali and 64.31% for Banglish. Hasan et al. [19] devised a stacked ensemble model to detect Bangla religious hate comments via a curated dataset of 3,003 labeled instances. They combined four ML classifiers as first-level learners with a neural network meta-classifier, achieving an overall accuracy of 96.6%, which significantly improved upon the individual base models’ 80.7% accuracy. Alvi et al. [20] designed a strategy for SA of Bangla textual data via a Gated RNN. The authors used an existing dataset from Hoque et al. [21] with a total number of samples of 7000 and trained this dataset via a GRU. They applied three rounds of 10-fold cross-validation, with the dataset changed in each round, and obtained a better average accuracy of 78.41%. Research conducted by Rahman et al. [22] aimed to classify Bengali sports news comments from different publications according to emotion. The writers gathered raw data consisting of 2,492 sentences from the renowned Bengali newspaper Daily “Prothom Alo”. They utilized many classic DL approaches, including CNNs, MLPs, and Long Short-Term Memory (LSTM). Among them, the CNN displayed greater performance. Bhowmik et al. [23] implemented a process using an extended lexicon dictionary and embedded supervised ML models in their proposed algorithm, the Bangla Text Sentiment Score (BTSC). The authors carried out their study using two datasets from Rahman et al. [24], representing two domains: crickets and restaurants. The first dataset contains approximately 2,900 comments on crickets, and the second dataset comprises approximately 2,600 reviews of restaurants. Using their proposed BTSC algorithm, the authors carry out sentence polarity categorization into three classes: Positive, Negative, and Neutral. In the case of the BiGram feature, the SVM has outperforms the other methods, with an accuracy of 82.21% in classification. Additionally, Ayman [25] et al. focused on Bengali text classification by distinguishing its two linguistic forms–-Sadhu and Cholito–-using DL models, where Bi-LSTM outperformed LSTM with 92.52% accuracy on a dataset of 7,350 texts from diverse sources, demonstrating its effectiveness in classifying formal and informal Bengali.

A study focused on an Attention-based CNN for Bangla sentiment analysis was conducted by Sharmin et al. [26]. The researchers prepared a corpus of 2,979 Bengali reviews and comments for this study. An accuracy of 72.06% was attained with their CNN model. Another study performed by Bhowmik et al. [27] covered an extended lexicon dictionary method with the help of a rule-based BTSC algorithm by changing DL algorithms, and was implemented in datasets collected from Rahman et al. [24]. BERT-LSTM, a hybrid model, outperforms the other models, with an accuracy of 84.18%. Parvin et al. [28] introduced an ensemble CNN-RNN architecture model for emotion classification on their dataset of 9,000 text data points with an F1-score of 62.46%. Amin et al. [29] proposed a method for implementing Bangla food review sentiment polarity analysis. The researchers gathered 1,484 Bangla reviews from several popular online food ordering platforms. Among all the algorithms examined, LR appeared to be the most accurate, with an impressive accuracy of 90.91%. Uddin et al. [30] carried out a study on the Bangla Social Media Depression Assessment by collecting 5,000 Bangla data samples from different micro-blogging sources and selected four hyperparameters optimized for constructing GRU models, reaching an accuracy of 75.7%. Another investigation led by Ahmed et al. [31] concentrated on detecting depression from Bangla social media text and included a total of 981 texts. They applied several classifiers, such as LSTM, GRU, SVM, and NB, to determine whether a social media post is depressive. Among them, LSTM with Stylometric features achieved a maximum accuracy of 76.42%.

Masum et al. [32] introduced “BAN-ABSA,” a manually annotated Bengali aspect-based sentiment analysis dataset of 9,009 distinct comments. Experiments on this corpus show that the CNN outperforms the other methods in both aspect extraction and sentiment classification, with accuracies of 79.09% and 71.48%. Junaid et al. [33] carried out sentiment analysis on Bangla food reviews via a balanced dataset of 1,040 manually labeled reviews. They have trained five traditional ML models and three DL models with different feature extraction techniques. The best accuracy for the ML models was from LR with 75% using TF-IDF, whereas for the DL models, the LSTM using Word2Sequence features had the best performance, with 90.86% accuracy. Lucky et al. [34] focused on detecting child-abusive public remarks in the Bangla language by gathering data from newspapers and social media platforms. The dataset includes 999 samples, which are evenly distributed across three categories: positive, negative, and neutral. The authors tested several ML and DL approaches, and the BERT model obtained the best accuracy of 88%. Biswas et al. [35] proposed a system for analyzing the sentiment of food delivery services by observing the reactions of users. The dataset consisted of 5680 remarks from users on various Facebook profiles. Afterward, they implemented five machine-learning techniques: BERT, Char-CNN, Graph-CNN, LSTM, and Bi-LSTM. BERT outperforms all other methods, with an accuracy of 92.86%. Sharif et al. [36] performed sentiment analysis on Online Restaurant Reviews. The dataset contains 1000 restaurant evaluations. The authors used DT, MNB, RF, and K-fold cross-validation for classification. Among these classification approaches, they concluded that MNB offers the maximum 80.48% accuracy for 6-fold cross-validation. In another work, Banik et al. [37] evaluated Bangla Textual Movie Reviews on a dataset consisting of 800 reviews, considering two categories: positive or negative. The authors employed two popular ML algorithms, NB and SVM, with several feature extraction methods. As an output, the SVM performed marginally better than the NB by observing the stemmed unigram as a feature with an outstanding precision of 0.86. Islam et al. [38] developed a technique to execute SA with the aid of a BERT pre-trained model. A total of 1,002 public newspaper comments were analyzed for sentiment. Finally, the GRU achieved 71% accuracy in binary sentiment classification. Hassan et al. [39] proposed an LSTM-based approach for the classification of data from various microblogging sites. The study was conducted on a dataset of 9,337 reviews to classify positive and negative sentiments and achieved 78% accuracy.

Table 1 shows an overall summary of current trends in sentiment analysis for Bangla language.

**Table 1:** Summary of recent research trends in Bangla sentiment analysis.

Reference	Domain	Size of the Dataset	Methods	Accuracy
Ullah [7]	Formal News Articles and Informal Social Media Comments	Formal (10,243) Informal (12,084)	**BCLSA** (CLSA-Bn)	90.2% (formal) 86% (informal)
Bhowmik et al. [8]	Bangla Text Sentiment Analysis (Multi-class)	15,114 samples	**BN-GRAPH** (Transformers + GNN + Data Augmentation)	89.57%
Shahnaz Ipa et al. [9]	Multilingual Product Reviews	3,864 reviews & ratings	**BdSentiLLM** (Llama-2)	89% (F1-score)
Rahman et al. [10]	E-learning reviews from YouTube	3178 (Bangla) 3090 (Romanized Bangla) 6268 (Combined)	NB, SVM, RF, **ANN**, CNN, BILSTM, BERT, BanglaBERT, Multilingual BERT, Bangla Electra, **XLM-ROBERTA**, CNN + MNB, CNN + Ensemble, CNN + BILSTM	89.46% (Bangla) 89.59% (Romanized Bangla) 85.81% (Combined)
Sunny et al. [11]	Product Reviews	5,016	**MLP**, SVM, RF, NB, KNN, ANN	96.42%
Hassan et al. [12]	Conversation Reviews (Film/Script)	1,141	**SVM**, NB, LR, KNN, DT, RF, SGD	85.59%
Mia et al. [16]	Music Lyrics	2,152	NB, SVM, SGD, XGBoost, MLP, BLSTM, **BERT**	65%
Faisal et al. [18]	Emotion (Bangla/Banglish Texts)	80,098	RF, NB, XGBoost, CNN, BiLSTM, CNN + BiLSTM, **BanglaBERT**, BanglishBERT, XLM-RoBERTa	71.12% (Bangla) 64.31% (Banglish)
Alvi et al. [20]	General Sentiment	7,000	**GRU with cross-validation**	78.41%
Khan et al. [17]	Bangla Text Sentiment Analysis	12000+	**LSTM**, Bi-LSTM	99%
Rahman et al. [22]	Sports News	2,492	**CNN**, MLP, LSTM	75.57% (F1-score: 2 classes) 48.19% (F1-score: 5 classes)
Bhowmik et al. [23]	Aspect-Based (Cricket & Restaurant)	~5,500	LR, **SVM**, KNN, RF	82.21%
Sharmin et al. [26]	News Comments	2,979	**Attention-based CNN**	72.06%
Bhowmik et al. [27]	Aspect-Based (Cricket & Restaurant)	~5,500	HAN+LSTM, D-CAPSNET+Bi-LSTM, **BERT+LSTM**	84.18%
Parvin et al. [28]	Emotion	9000	**CNN + BiLSTM**	62.46% (F1-score)
Amin et al. [29]	Food Reviews	1,484	**LR**, SVM, NB	90.91%
Uddin et al. [30]	Depression Analysis	5,000	**GRU**	75.7%
Ahmed et al. [31]	Depression (FB Posts)	981	**LSTM**, GRU, SVM, NB	76.42%
Masum et al. [32]	Aspect-Based Food Comments	9,009	CNN, **BiLSTM**	79.09%
Junaid et al. [33]	Food Reviews	1,040	RF, SVM, MNB, LR, DT, **LSTM**, GRU, RNN	90.86%
Lucky et al. [34]	Child Abuse Detection	999	MNB, RF, DT, SVC, KNC, LR, RNN, **BERT**	88%
Biswas et al. [35]	Food Delivery Services	5,680	**BERT**, Char-CNN, Graph-CNN, LSTM, BiLSTM	92.86%
Sharif et al. [36]	Restaurant Reviews	1,000	DT, **MNB**, RF	80.48%
Banik et al. [37]	Movie Reviews	800	NB, **SVM**	86%
Islam et al. [38]	Newspaper Comments	1,002	BERT, **GRU**	71%
Hassan et al. [39]	Microblog Sentiment	9,337	**LSTM**	78%

## Methodology

In this section, the methodology that explicitly outlines the overall procedures of the research is presented. A dataset for sentiment analysis specifically in the Bangla language was created. Furthermore, an experimental framework was developed to integrate machine learning methods, deep learning methods, ensemble methods (specifically Stacking), and transformer-based models (such as BanglaBERT) for sentiment prediction. The dataset includes three classes: Positive, Negative, and Neutral. Fig 2 illustrates the complete workflow involved in the proposed research.

**Fig 2:**
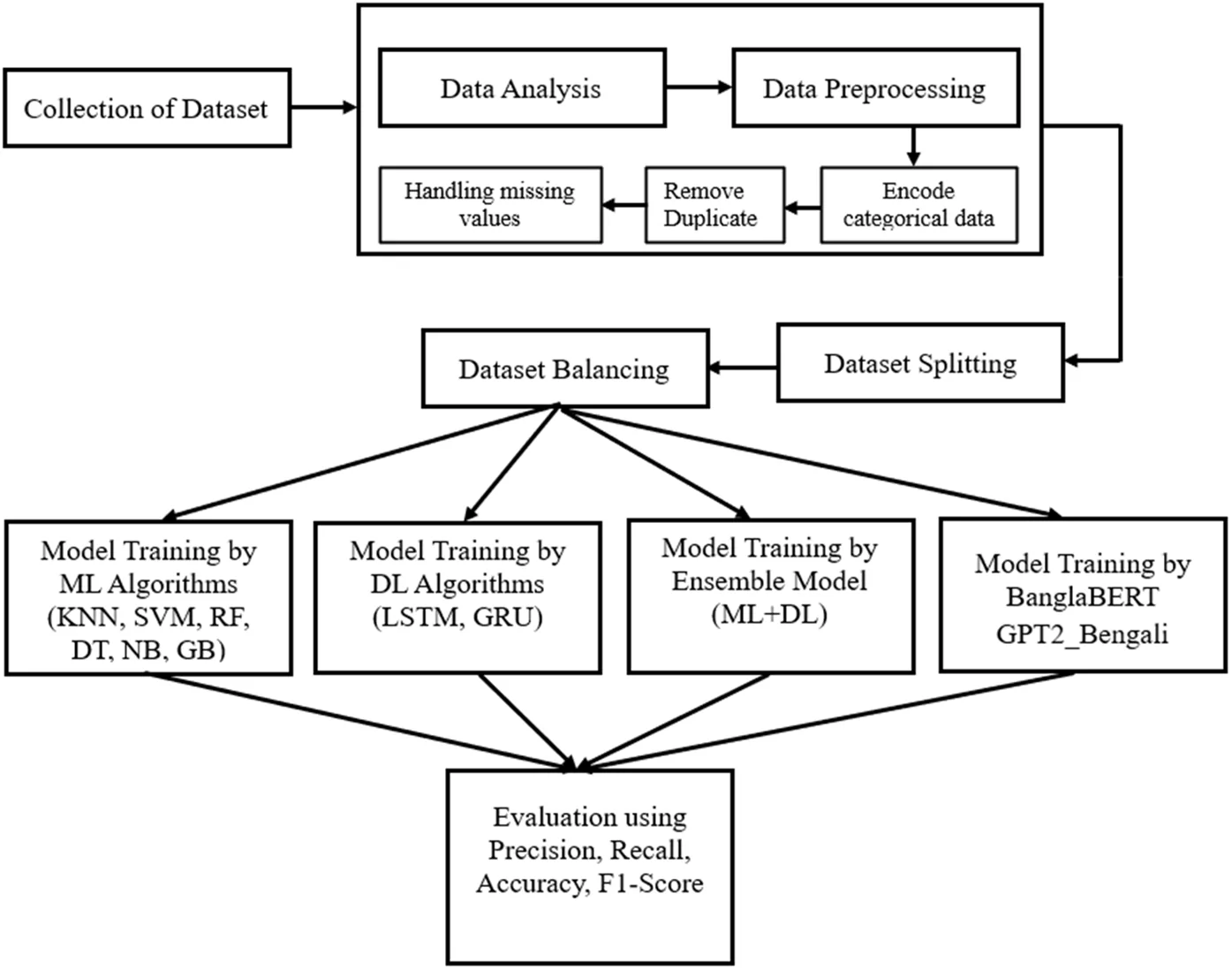
Schematic representation of our workflow.

### Data description

The Bangla sentiment dataset comprises a total of 9163 different comments from different online social media platforms. Most of the data are compiled from Facebook pages (https://www.facebook.com/DailyProthomAlo/; https://www.facebook.com/BBCBengaliService/). Some data are extracted from some popular Bengali Websites: BBC Bangla, YouTube Channel, Bangla Newspaper, X, etc. The authors of the paper collected the dataset manually. The length of the comments consists of approximately 3–120 words. There are several reasons for selecting these Websites for compiling comments. BBC Bangla and Prothom Alo are widely recognized online Websites among the Bengali-speaking community. Similarly, Facebook, YouTube, and other Bengali Newspapers are also popular for publishing reliable and authentic events. Over 100 million Bengali-speaking people use these websites, with approximately 60 million social media users from Bangladesh [40,41]. Most readers prefer expressing their opinions in Bangla in the comment section. The dataset includes comments related to various topics that were shared recently. Users typically express their sentiments, opinions, or suggestions in response to the shared news content. Most of the time, the comments about the events were in Bangla. In some cases, comments were written in English, or Bangla was transliterated using the English alphabet; such comments were not included in the dataset. Similarly, comments containing only sentiments expressed through emojis without any textual content were also omitted from the dataset. The authors worked on all the processes manually to create the dataset. To guarantee that only publicly accessible content was utilized for analysis, all the data were gathered in accordance with the terms and conditions of the corresponding platforms.

**Ethical considerations: A**ll data used in this study were collected from publicly accessible online platforms in accordance with the respective platforms’ terms and conditions. The dataset consists solely of publicly available user comments, and no private, restricted, or personally identifiable information was collected or stored. Usernames, profile details, and metadata were excluded to ensure anonymity. The study did not involve any direct interaction with the original content creators. As this work constitutes a secondary analysis of public data, informed consent from individual users was not required. In accordance with institutional guidelines, this component of the study was exempt from Institutional Review Board (IRB) review, and ethical approval and participant consent were therefore waived.

For data annotation, a group of native Bangla-speaking volunteers participated in labeling the dataset. All annotators provided informed consent prior to participation. Participation was entirely voluntary, and annotators were informed about the purpose of the study, the nature of the annotation tasks, the expected time commitment, and their right to withdraw at any time without consequence. Consent was obtained verbally using a standardized consent script. Verbal consent was documented by the study coordinator through written confirmation in the annotation records and was witnessed by at least one member of the research team. No personal, identifying, or demographic information was collected from the annotators. This annotation procedure was conducted in accordance with institutional ethical guidelines, and formal IRB approval was not required for this minimal-risk study involving voluntary adult participants.

#### Dataset annotation.

The comments of the Bangla text on the Sentiment dataset were annotated together by the authors, a group of third-year students of B. Sc. in CSE, and two graduate students from the Department of CSE, Dhaka International University, Bangladesh. All the volunteers agreed to categorize the dataset into three different classes of sentiment: Positive, Negative, and Neutral. A comment was given, and the responsibility of the volunteers was to suggest the sentiment individually. Table 2 shows the details of the volunteers included in the annotation.

**Table 2:** Information about the volunteers in data annotation.

Volunteers ID	Gender	Profession	Responsibility
V1	Male	Faculty/Author	Data Collection and Annotation
V2	Male	Faculty/Author	Data Collection and Annotation
V3	Male	Faculty/Author	Data Collection and Annotation
V4	Female	MS Student	Annotation
V5	Male	B.Sc. Student	Annotation
V6	Female	B.Sc. Student	Annotation
V7	Female	B.Sc. Student	Annotation
V8	Male	B.Sc. Student	Annotation
V9	Female	B.Sc. Student	Annotation
V10	Male	Program Officer	Annotation

Each volunteer participated in categorizing every comment on the dataset by providing their opinion. The majority voting technique was applied to determine the final sentiment of that sentence [42]. For example, the following comment was considered:

**“আমাদের দেশের ব্যবসায়িরা কসাই থেকে ও খারাপ”**

**(The businessmen of our country are worse than butchers)**

The voting system of our approach and the results are presented in Table 3.

**Table 3:** Category identification by voting system.

Comment: {আমাদের দেশের ব্যবসায়িরা কসাই থেকে ও খারাপ} (The businessmen of our country are worse than butchers.)	
**Volunteer ID**	**Selected Sentiment**
V1	Negative
V2	Negative
V3	Negative
V4	Negative
V5	Neutral
V6	Negative
V7	Negative
V8	Negative
V9	Negative
V10	Negative

From Table 3, it is noticeable that the comment received nine votes for Negative sentiment, whereas only one vote was for the Neutral class. As a result, the technique classified this comment as belonging to the Negative sentiment class. In Table 4, the comment had six votes for the Neutral sentiment class and four votes for the Negative class. Therefore, the method assigns the neutral sentiment label to that comment. In contrast, situations occasionally arose in which 50% of the participants selected the Positive class and the remaining 50% selected the Neutral class. In such cases, an additional volunteer was assigned to determine the sentiment of the comment. The votes were then recounted to finalize the sentiment class. Table 5 presents a small portion of the labeled dataset.

**Table 4:** Category selection of sentiment.

Comment: {সরকারের উচিত তেল ব্যাবহারে কি ভাবে করা উচিত তা জনগনকে শিক্ষানো} (The government should teach people how to use oil)	
**Volunteer ID**	**Selected Sentiment**
V1	Negative
V2	Neutral
V3	Neutral
V4	Negative
V5	Neutral
V6	Negative
V7	Neutral
V8	Neutral
V9	Negative
V10	Neutral

**Table 5:** Dataset sample.

Comment	Sentiment
{প্রেমের টানে বাংলাদেশের অর্থনীতি শ্রীলঙ্কার দিকে} (Bangladesh’s economy towards Sri Lanka)	0 (Negative)
{জিনিসপত্রের দাম বাড়তেই পারে} (Prices of goods may increase)	2 (Neutral)
{বতর্মান সংকট থেকে উতরানের জন্য জনগণের ঐক্যবদ্ধ হওয়া উচিৎ} (People should get united to get out of the current crisis)	1 (Positive)
{আমি ডিম খাইনা} (I do not eat eggs)	2 (Neutral)
{দ্রব্যমূল্যের দাম বৃদ্ধিতে সাধারণ মানুষ এত অসহায়.} (The common people are so helpless due to the price hike of commodities)	0 (Negative)
{অসাধারণ উন্নয়ন জোয়ার এসেছে আামাদের দেশে.} (Tremendous development has come to our country)	1 (Positive)
{সরকার কি করে আমার মাথায় আসেনা সাধারন মানুষ কিকরে চলবে} (I don’t know how the government will do it, how the common man will run)	2 (Neutral)
{পেট্রোল পাম্পে আগুন লাগাও.} (Set fire to the petrol pump)	0 (Negative)
{ভারতের মতো টিকিট সিস্টেম চালু করলে বারতি ভারা নিতে পারবেনা} (If you start a ticketing system like India, you will not be able to charge)	1 (Positive)
{রিজার্ভ শুন্য স্টক বাড়াতে গরিবের পিঠে লাথি} (Kick in the back of the poor to increase the reserve stock)	0 (Negative)

In Table 6, the final statistics of the complete dataset are presented. A total of 9163 different comments were gathered, which were categorized into three different sentiment classes: Positive, Negative, and Neutral. Many comments were categorized into the Negative class, whereas 2829 and 2192 comments were categorized into the positive and neutral classes, respectively.

**Table 6:** final statistics of bangla sentiment dataset.

Sentiment	No. of Comments
Negative (0)	4139
Positive (1)	2189
Neutral (2)	2826
**Total**	**9154**

#### Analysis of the proposed dataset.

To analyze the proposed dataset, Zipf’s Law was applied to examine the frequency distribution of all words in the dataset [43]. A common pattern is revealed via Zipf’s Law in natural language processing. Typically, this law counts all the words contained in the dataset, where the word frequencies decrease at a high rate as their position decreases, indicating that a limited set of words is utilized extensively, whereas the majority of words are rarely found. For instance, published findings on sentiment datasets in Bangla, such as BangDSA and KUMono ensure that the distributions of the word frequency align with this pattern, revealing that these datasets precisely capture the natural language usage [44]. Equation (1), expresses the inverse relationship between the rank and frequency. Finally, Fig 3 illustrates the word frequency distribution of our Bangla Sentiment dataset.


$$\begin{array}{c} f(r)=\frac{C}{r^{s}} \end{array}$$ (1)

where *f*(*r*) is the word frequency at rank *r*, *C* is the balance constant, and *s* is a parameter that is close to 1 for natural languages.

**Fig 3:**
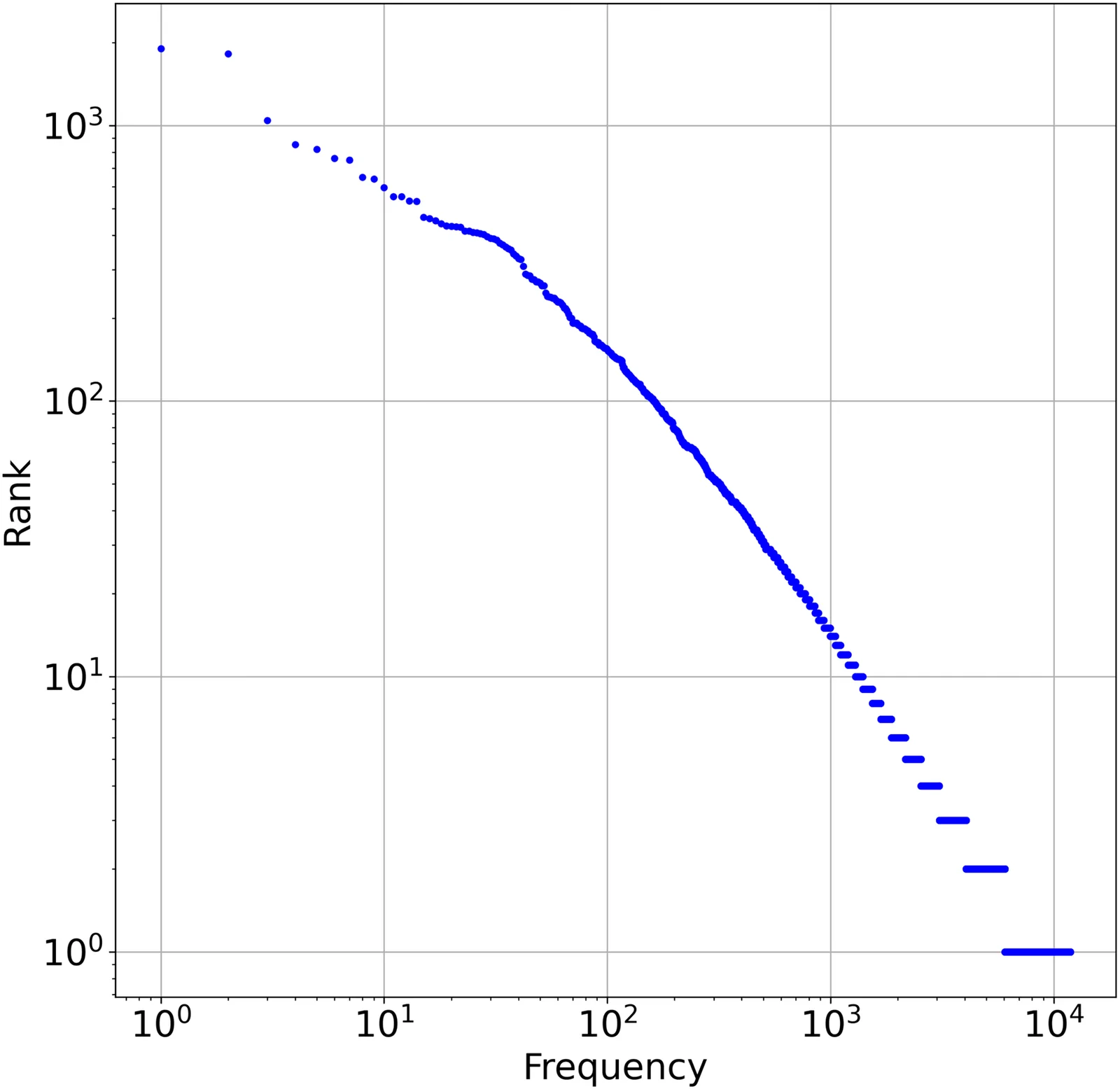
Word frequency distribution using Zipf’s Law.

### Data preprocessing

Data preprocessing is one of the most fundamental factors of machine learning and deep learning frameworks, specifically when working on text-based data such as Bangla. Generally, the raw text may contain inconsistencies, informal expressions, missing values, and non-standard forms that could hamper effective feature extraction. However, if the dataset is in the Bangla language, it could be more complex to analyze, classify, and predict due to its complex morphology, inconsistent spelling, and high-quality annotated corpora. As a result, to confirm accurate sentiment classification from Bangla text data, a structured preprocessing workflow was conducted, as shown in Fig 4. The preprocessing of this study was divided into four different aspects: Filtering, Extension, Lemmatization, and Tokenization.

**Fig 4:**
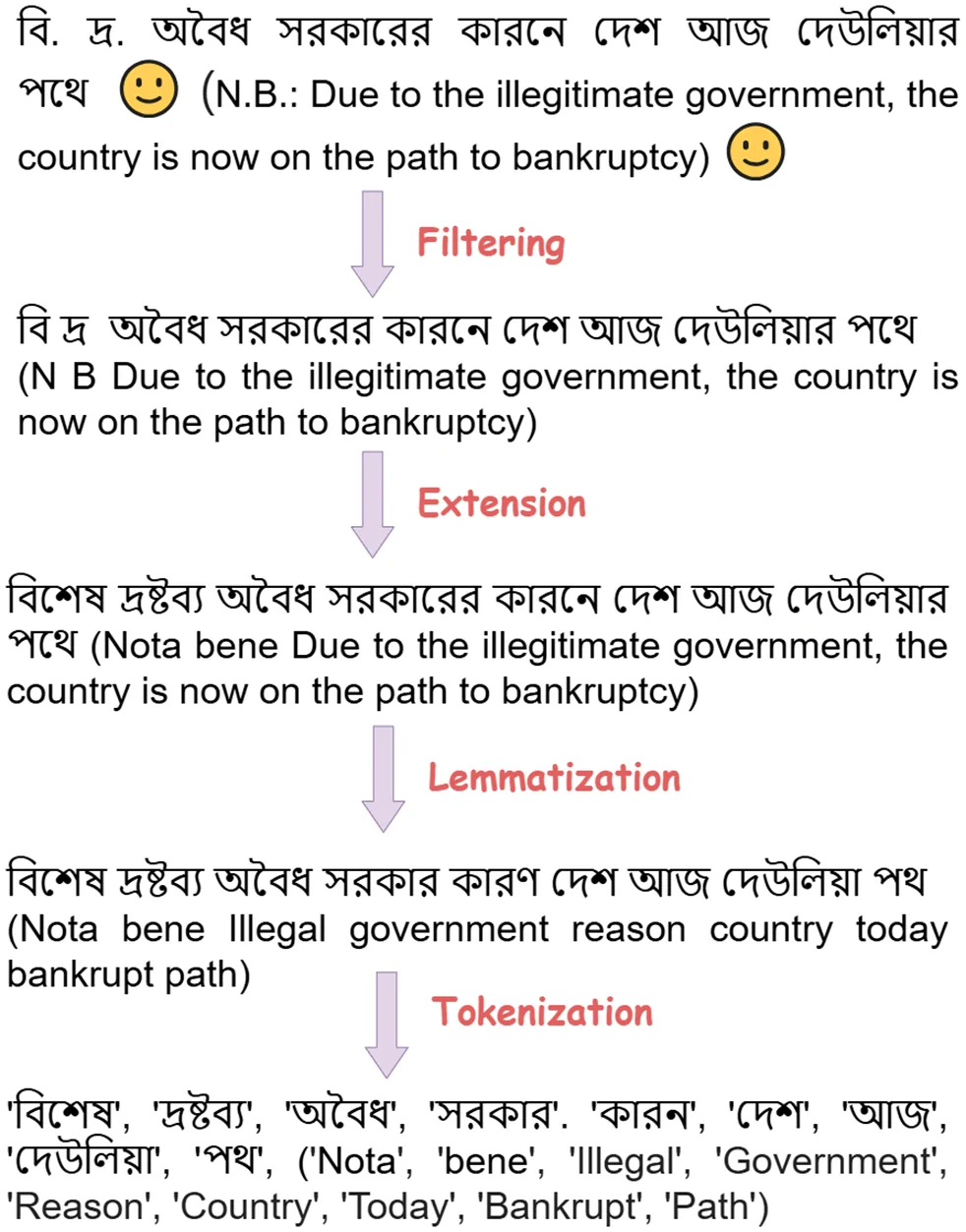
Data preprocessing procedures.

- First, the dataset was cleaned by filtering out unnecessary segments such as punctuation marks, special characters, emojis, and repetitive whitespaces. Similarly, any missing or null values were determined and suitably managed by omitting the row to maintain data integrity. These types of activities are collectively referred to as filtering.
- In the Bangla language, many short forms are used by Bangla-speaking people. It is impossible to understand the actual meaning of those short forms for machines. Consequently, abbreviated or short-form expressions must be extended to their full forms to enhance semantic clarity. In this study, we have expanded the short-form expressions shown in Fig 4.
- There are many extended forms of words used in the Bangla language. Lemmatization is a technique that is employed to discover the actual root or base form, which assists in shrinking various inflected versions of the same word. This method was utilized in the dataset to make the root or base form.
- Finally, the comments or sentences were separated into different word tokens. This technique was executed via Bangla-specific rules to precisely split the words, according to the language’s morphological complexity. The generated tokens are used as the input for the feature engineering and modeling phases.

### Data balancing

Handling the imbalance class is a crucial phase in prediction problems, as the applied models are trained on imbalanced datasets that could be biased toward the majority class or usually neglect the minority class, which may lead to inappropriate predictions. An imbalanced dataset can substantially decrease the ability of the utilized model to generalize well across all classes [45]. To overcome this challenge, SMOTE technology was introduced, which yields new, synthetic examples for the minority class by approximating intermediate samples on the basis of local neighbors [46–48]. This method assists in obtaining a more balanced class distribution without relying solely on copies of the original data, thereby enhancing the model’s learning ability [5]. For example, Fig 5 illustrates the data distribution of our dataset, where Fig 5(a) displays the imbalanced distributions and Fig 5(b) presents the balanced dataset after the SMOTE method is utilized. Before the dataset is balanced, the negative class has the most data with 4139. The Neutral class conveys approximately 2826 data points, and the Positive class consists of 2189 data points. These are fewer than those of the negative class. After applying SMOTE, all the classes contain the same amount of data as the Negative class shown in Fig 5(b).

**Fig 5:**
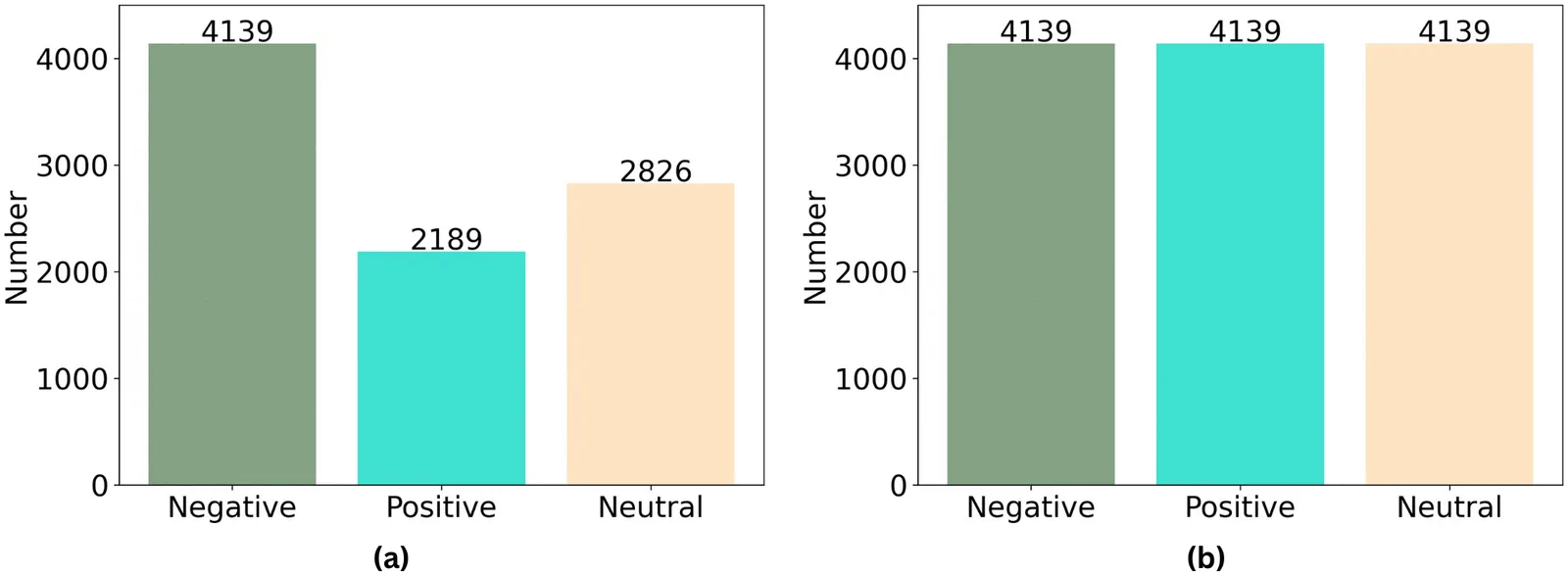
Dataset distribution: Before and after balancing.

### Data encoding

Several encoding techniques exist to convert text data to numeric data. Two encoding methods namely Word2Vec and Count Vectorizer was employed in this study. Word2Vec is utilized for neural network models whereas, Count Vectorizer is used for machine learning models. In word2vec, a widely used embedding method, addresses this need by mapping words into dense vector spaces, where the text data are represented via vectors. These vectors try to find inherent semantic representations of that word, meaning that the representations of the words king and queen will be similar. This is achieved by learning from a large text corpus. The Word2Vec model uses a 100-dimensional vector representation for each word. In contrast, the Count Vectorizer is also a popular choice for encoding numeric data. It creates a sparse matrix to represent each sentence where it counts each word’s frequency. The disadvantage of using a count vectorizer is that it does not capture the inherent semantic relationships among words, but the advantage is its simplicity. A single word in a sentence is often enough to label the sentence with negative sentiment. This is the reason for using a count vectorizer for machine learning algorithms.

### Applied models architectures

To obtain accurate predictions, various types of models were introduced in this study. The performance of the models was evaluated by partitioning the dataset into training (70%) and testing (30%) sets. Initially, several supervised machine learning classifiers were applied to the preprocessed dataset. Subsequently, two deep learning models were incorporated to improve the overall performance. To further enhance the results, an ensemble method called Stacking was utilized, which combines five machine learning models and two deep learning models. Additionally, a transformer-based model, BanglaBERT, was employed to achieve satisfactory outcomes in the investigation.

#### Supervised machine learning models.

Numerous supervised machine learning algorithms exist, and selecting the most suitable algorithm for a particular dataset could be a demanding and crucial decision for any researcher. An extensive review of the existing literature was conducted, and various model architectures and methods were analyzed to determine the most effective classifiers for the task. As a result, six supervised ML classifiers, namely NB, RF, DT, SVM, KNN, and XGBoost, were introduced in the proposed system.

**Multinomial Naive Bayes Classifier** The Multinomial Naive Bayes model is a probabilistic learning technique normally applied for text classification tasks, specifically in natural language processing. Bayes’ theorem is used to evaluate the model and considers that the features are conditionally independent given the class label. The model is incredibly useful when features convey frequency counts or term frequencies in records. The probability of a record *d* belonging to a class *c* is evaluated via Equation (2).


$$\begin{array}{c} P(d)\propto P(c)\prod_{i=1}^{n}P(w_{i}|c{)}^{x_{i}} \end{array}$$ (2)

**Random Forest Classifier** The Random Forest is itself an ensemble model in which multiple decision trees are created to achieve better outcomes and become more robust. During training, many decision trees are created, each of which is trained via a different subset of the features and data. By combining the results of each individual tree, the final forecast is produced, usually by majority voting for categorization. Equation (3), yields the final prediction $\hat{y}$ for a sample.


$$\begin{array}{c} \hat{y}=arg\sum_{i=1}^{T}I(h_{i}(x)=c) \end{array}$$ (3)

**Decision Tree Classifier** The Decision Tree classifier is a widespread supervised learning algorithm frequently applied in text classification schemes such as sentiment analysis. From the perspective of Bangla sentiment analysis, the model assists in categorizing textual inputs into sentiment classes such as positive, negative, or neutral. It constructs a tree-based modeling approach where internal nodes denote decisions on the basis of specific lexical and syntactic features, and leaf nodes suggest sentiment classes. Furthermore, at every decision node, the model specifies the feature that delivers the highest discrimination among sentiment categories. This selection is generally driven by Information Gain (*IG*), which depends on the concept of Entropy (*H*). For dataset *D* which consists of *C* sentiment classes, the calculations of the entropy and the Information Gain are demonstrated via Equation ((4a).)-((4b).), respectively.


$$\begin{array}{c} H(D)=-\sum_{i=1}^{C}p_{i}log_{2}(p_{i}) \end{array}$$ (4a)

$$\begin{array}{c} IG(D,A)=H(D)-\sum_{v\in Values(A)}\frac{\left|D_{v}\right|}{\left|D\right|}H(D_{v}) \end{array}$$ (4b)

**Support Vector Machine Classifier** Support Vector Machine (SVM) is a robust supervised learning algorithm that is effective for classifying text data, including Bangla sentiment analysis. It performs by discovering an optimal hyperplane that splits the sentiment classes (e.g., positive, negative, and neutral) with the highest margin. By using the kernel functions, the SVM can manage nonlinear data, and assemble them appropriately for high-dimensional, sparse text features such as TF-IDF or word embeddings. Equation (5), expresses how the SVM classifier solves the problem with a given training dataset, where $x_{i}$ is the feature vector and $y_{i}$ represents the class labels.


$$\begin{array}{c} \frac{1}{2}||\omega |{|}^{2}\text{ Subject to }y_{i}(w.x_{i}+b)\geq 1 \end{array}$$ (5)

**K-Nearest Neighbors Classifier** K-Nearest Neighbors (KNN) is a lightweight but powerful supervised machine learning classifier employed for categorical tasks. In this study, KNN allots a sentiment label (positive, negative, or neutral) to a text on the basis of the majority sentiment among its k closest texts in the training data. Equation (6), depicts how this model generally measures closeness via a distance metric such as the Euclidean distance.


$$\begin{array}{c} d(x,x)=\sqrt{\sum_{i=1}^{n}(x_{i}-y_{i}{)}^{2}} \end{array}$$ (6)

Here *x* and $x_{i}$ are feature vectors dragged from Bangla text (e.g., using TF-IDF). The model does not demand training but makes projections by comparing new samples with labeled examples.

**Extreme Gradient Boosting Classifier** Extreme Gradient Boosting is a highly effective ensemble learning algorithm based on decision trees. Advanced features, including regularization, parallel processing, and optimal data handling, are added to this method via XGBoost, which improves accuracy and speeds up training. In this Bangla sentiment analysis study, XGBoost estimates sentiment classes by integrating the outcomes of multiple weak learners (trees) sequentially. Each new tree concentrates on repairing the errors of the previous trees by minimizing the regularized objective function demonstrated in Equation (7), This model can effectively manage high-dimensional feature representations of Bangla text data and delivers strong performance on sentiment classification schemes.


$$\begin{array}{c} L=\sum_{i=1}^{n}l(y_{i},{\hat{y}}_{i})+\sum_{k=1}^{k}\Omega (f_{k}) \end{array}$$ (7)

#### Deep learning models.

To obtain better outcomes, several robust Recurrent Neural Network (RNN) models, specifically LSTM and GRU, were introduced. For instance, LSTM and GRU models are essential for Bangla text classification because of their ability to capture long-range dependencies and contextual information in sequential data [49]. These models can manage the morphological richness and syntactic complexity of the Bangla language more effectively than traditional methods can.

**Long Short-Term Memory Model** Long Short-Term Memory (LSTM) is an advanced form of the Recurrent Neural Network (RNN) that effectively models sequential data by maintaining important knowledge over long text sequences. In the context of Bangla sentiment analysis, LSTM comprehends the contextual relationships between words to categorize text into positive, negative, or neutral aspects. It comprises specialized gates–-namely input, forget, and output gates–-that handle the flow of information via the network, assisting in overcoming problems such as the vanishing gradient issue found in traditional RNNs. In this study, both Word2Vec and FastText embeddings were utilized for tokenization. The LSTM had an input size of 100 with a hidden size of 150. Then it passes through 6 linear layers, which also include the output layer. The architecture of the linear layers was as follows (4096, 1024, 512, 128, 64, 3), as shown in Fig 6. Dropout is used after every linear layer except the output layer, and the value for the dropout is 0.4 with the in-place option being false. The number of epochs was 900, the batch size was 1000, and the learning rate was 0.0001. Weight_decay is also applied to mitigate memorization with a value of 0.001. The best weights were captured during training.

**Fig 6:**
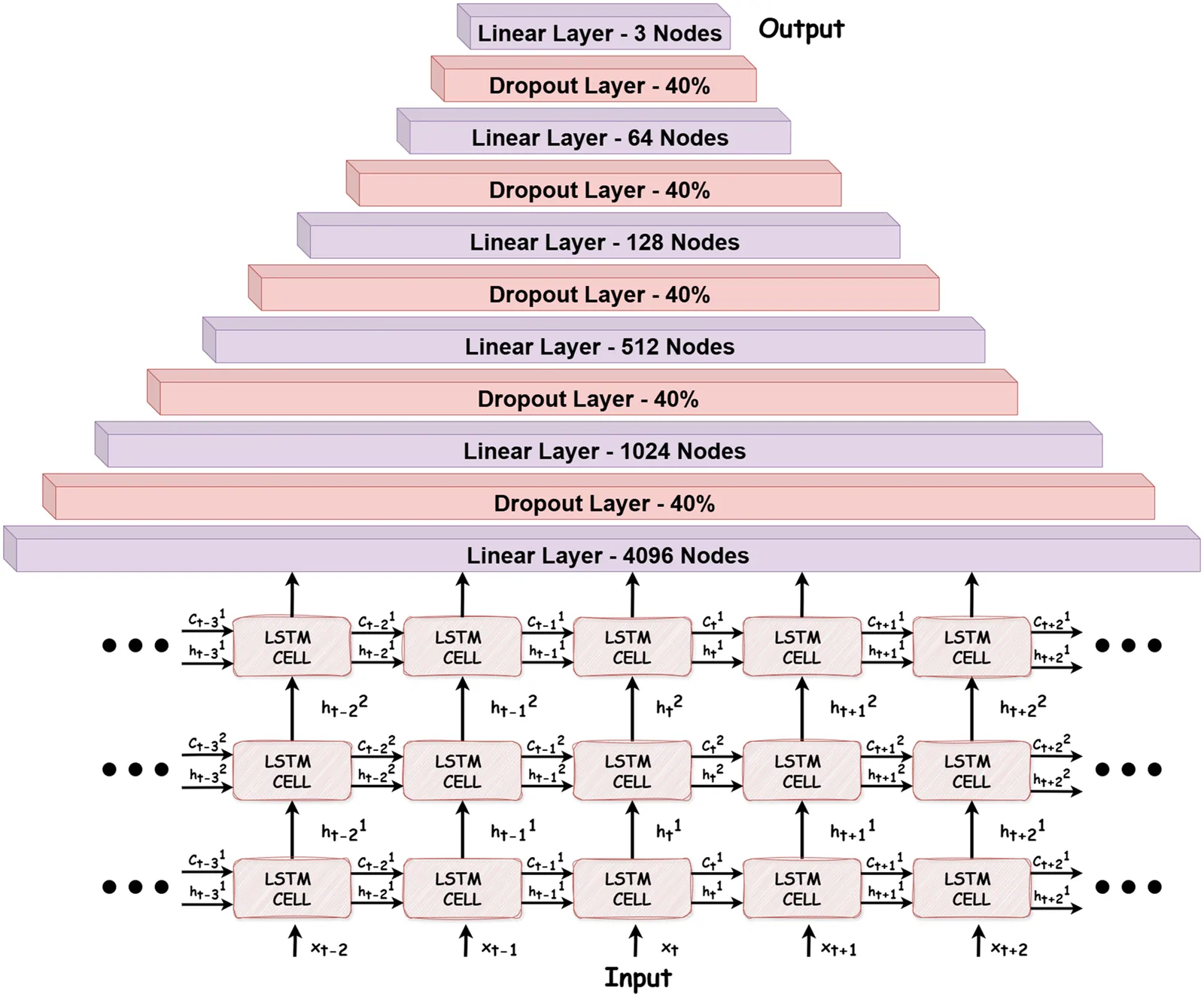
Deep LSTM model’s architecture.

**Gated Recurrent Unit Model** The Gated Recurrent Unit (GRU) is an optimized version of the traditional Recurrent Neural Network (RNN) that extracts and preserves temporal relationships in input sequences. For Bangla sentiment analysis, the GRU captures sequences of words and selects whether the sentiment is positive, negative, or neutral by comprehending their context. Unlike LSTM, GRU uses a simplified design with two main gates: an update gate that integrates the role of memorizing and forgetting, and a reset gate that handles how past information affects the current input. This design lowers the computational cost while maintaining strong performance in selecting essential sentiment-related patterns in Bangla text. In this study, both Word2Vec and FastText embeddings were utilized for tokenization. Two gated recurrent units were used one after another and then passed through six linear layers. The GRU had an input size of 100 with a hidden size of 150. Among the six linear layers, the last layer is the output layer. The architecture for the linear layers was (4096, 1024, 512, 128, 64, 3), as displayed in Fig 7, which is the same as that of the LSTM layers. The number of epochs is 200 with a batch size of 1000, and the learning rate equals 0.001. The Dropout layers and weight_decay are the same as those in the previous model. The best weights were also captured for the models.

**Fig 7:**
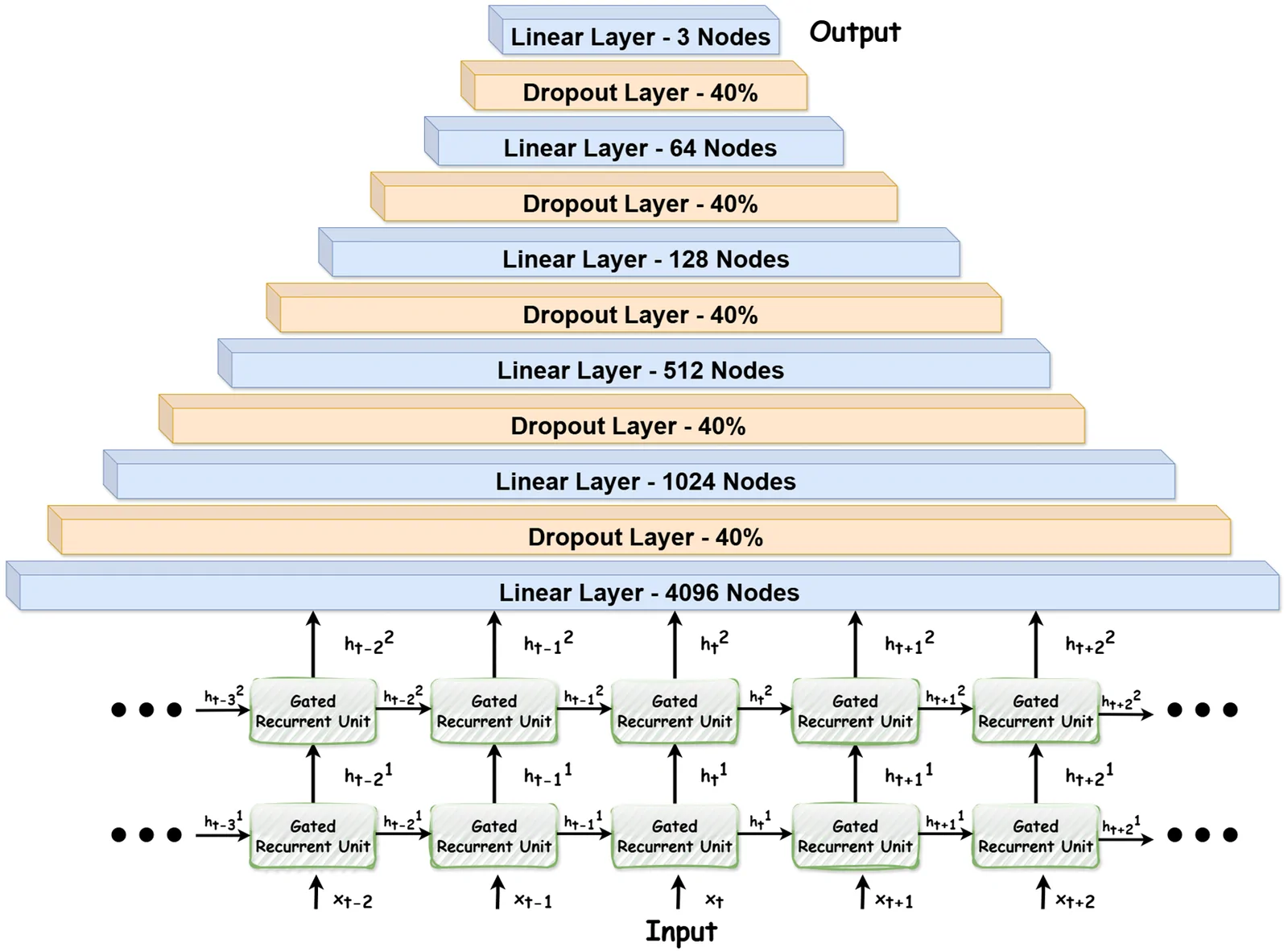
Deep GRU model’s architecture.

#### Ensemble model (stacking).

Sometimes, machine learning and deep learning models are not capable of producing the best results by themselves alone. In these scenarios, an ensemble model can help produce more acceptable results. The ensemble model combines the performance of several models to achieve better accuracy. Broadly, ensemble models can be divided into three types: bagging, boosting, and stacking. Among them, a stack-based ensemble model is often preferred because it uses another predictive model to produce the final output from the input of the previous layers’ model’s output. The two components of a stack-based ensemble model are referred to as the level 0 and level 1 classifiers. The dataset is used by the Level 0 classifier to generate the output. The output of the level 0 classifiers is then used to create a new dataset. To generate the actual result, the level 1 classifier is trained on the new dataset. Seven models are utilized in total for the level 0 classifier. Five of them are machine learning models, excluding the Multinomial Naive Bayes classifier, and the remaining two are deep neural networks. The outputs of these 7 models were utilized to construct a new dataset. This new dataset is then applied to the level 1 classifier. A neural network was used for the level 1 classifier. The architecture of this model is (7, 128, 64, 32, 3), as illustrated in Fig 8. All three layers are linear layers. The first layer is the input layer, followed by a hidden layer and an output layer. This output layer produces the final output. The learning rate, number of epochs, and batch size were 0.001, 100, and 100, respectively.

**Fig 8:**
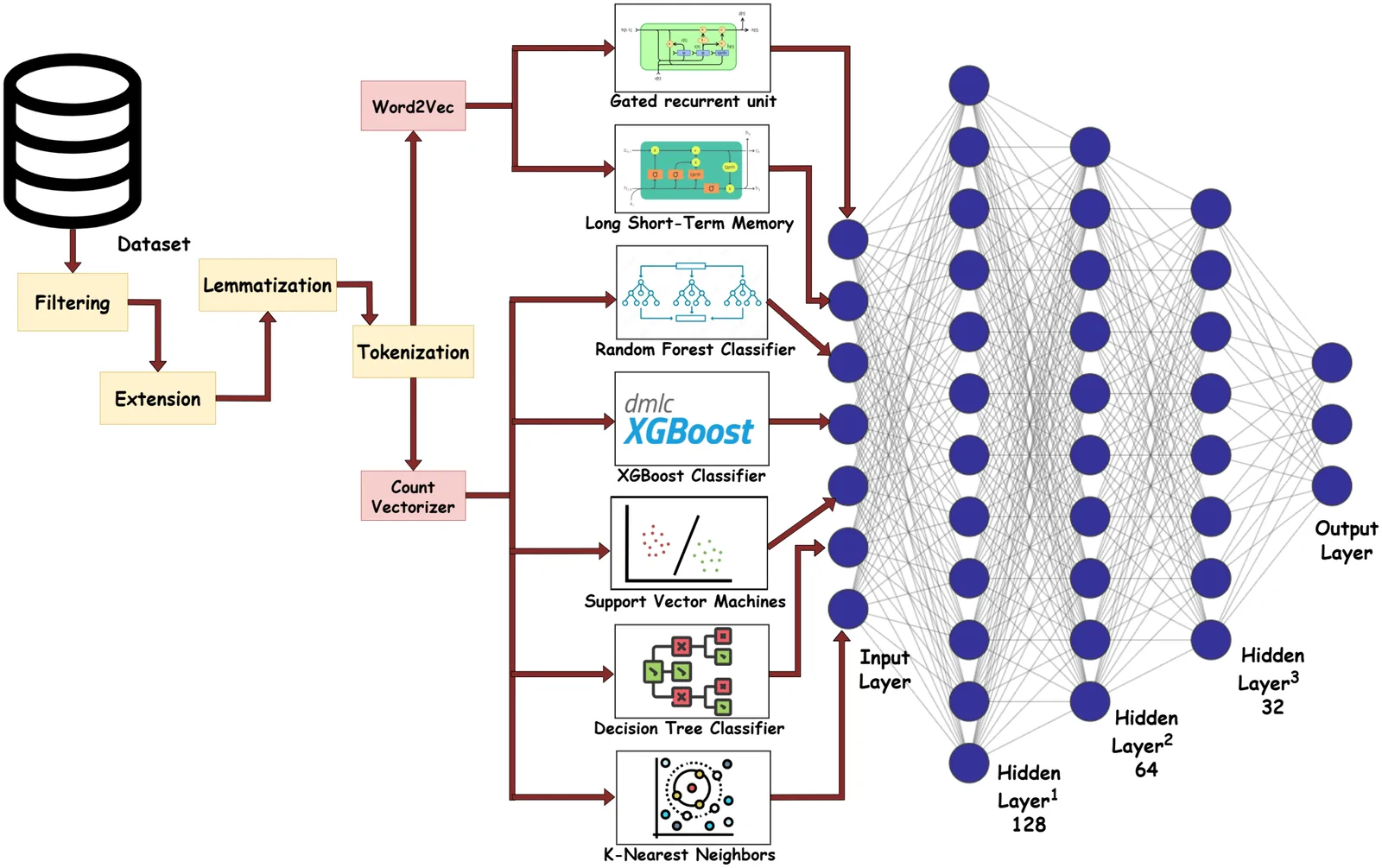
Ensemble (Stacking) model’s architecture.

#### BanglaBERT model.

The BanglaBERT model plays a significant role in natural language processing studies concerning Bangla datasets, especially because of the linguistic complexity and limited-resource nature of the Bangla language [50]. In this research, the BanglaBERT technique, which is based on the transformer architecture, was applied to increase sentiment analysis performance for the Bangla dataset. Specifically, the pretrained model “sagorsarker/bangla-bert-base” was employed because of its rich linguistic representation comprehended from large-scale Bangla corpora [51]. The BERT tokenizer linked with this model was utilized for tokenization, with the highest sequence length set to 150 tokens to balance the scope of context and computational performance. The fine-tuning procedure was carried out over 50 epochs, with a batch size of 128, and the AdamW optimizer with a learning rate of 2e-5 was used to confirm robust and optimized gradient updates. The model’s performance is obtained through the softmax function executed to the final hidden state of the [CLS] token depicted in Equation (8).


$$\begin{array}{c} \hat{y}=softmax(W.h_{\left[cls\right]}+b) \end{array}$$ (8)

where *h*_[*CLS*]_ defines the contextual embedding of the classification token, *W* is the weight matrix, *b* is the bias, and $\hat{y}$ indicates the expected probability distribution over sentiment classes. This setup facilitated the model in determining deep semantic patterns in Bangla, leading to superior prediction performance in comparison with traditional models.

#### GPT2_BENGALI model.

The GPT-2 BENGALI model was employed to investigate the effectiveness of autoregressive transformer architectures for sentiment analysis on Bangla text. GPT-2 is a decoder-only transformer that captures contextual dependencies through causal self-attention, making it suitable for sequence modeling tasks. In this study, pretrained GPT-2 BENGALI variants were utilized to leverage linguistic knowledge learned from large-scale Bangla corpora. The tokenizer associated with the GPT-2 model was used for subword tokenization, with the maximum sequence length fixed at 100 tokens to balance contextual coverage and computational efficiency. Since GPT-2 does not define a padding token, the end-of-sequence token was assigned as the padding token to ensure consistent input formatting. The model was fine-tuned using the AdamW optimizer with a learning rate of 2e-5 and a batch size of 16. Fine-tuning was conducted for 20 epochs, as GPT-based models typically converge rapidly. Gradient clipping was applied to stabilize training, and a linear learning rate scheduler was employed to ensure smooth optimization.

### Use of generative AI

The authors used a small amount of AI writing assistance in the preparation of the manuscript, but only for editorial help. These tools were not utilized in any aspect of the research design, code creation, data collection, analysis or interpretation.

- ChatGPT (OpenAI) served only to help arrange and polish the structure of this manuscript and to refine its clarity, coherence, and logical flow. The tool did not produce any novel data, source code or research data, results, or interpretations.
- Grammarly was used to help correct grammar and typos. The authors checked all the automated suggestions to ensure the precision and quality of the text.
- QuillBot was even used slightly to paraphrase some of the authors’ own writing of the Introduction and Literature Review to achieve greater readability. Source material or published content created by a third-party was not input into the tool, and it should not be used to generate original work for academic purposes.

These tools were applied ethically and transparently, and all the scientific work was still first-hand crafted and authored by the researchers.

## Results and discussion

This section presents the assessment results of the proposed system, including the applied machine learning classifiers, deep learning models, and transformer-based models (specifically BanglaBERT). To obtain reliable outcomes, the dataset was subjected to a complete preprocessing pipeline that included filtering, extension, lemmatization, and tokenization. To evaluate model performance, a 3×3 confusion matrix was constructed to assess how effectively the models classify instances according to their sentiment levels. The analysis of each successful method emphasizes the importance of performance evaluation metrics such as accuracy, precision, recall, and F1-score, thereby strengthening the validity of the outcomes. Additionally, model performance is further illustrated through newly constructed ROC curve plots, along with training versus testing accuracy and training versus testing loss graphs for the deep learning and BanglaBERT models.

### Performance of machine learning classifiers

In this study, several machine learning techniques, such as NB, RF, DT, SVM, KNN, and XGB, were initially applied to the Bangla dataset using their default hyperparameters. Fig 9 depicts the confusion matrices of the applied ML classifiers across three sentiment classes. Fig 9(b) and 9(f) illustrate the XGB and RF classifiers, respectively, which exhibit excellent performance, with higher true positive rates across all classes, particularly outperforming in correctly recognizing negative and neutral instances. The KNN Classifier shown in Fig 9(d) results in moderate outcomes for the positive class but incorrectly labels a remarkable number of negative sentiments. The SVM and MNB are displayed in Fig 9(a) and 9(e), respectively, which generate better-balanced projections but with inferior overall performance, especially indicating misclassification between negative and neutral classes. In Fig 9(c), the DT displays effectiveness adequately, with an empirical bias toward the positive instance. To identify the better-performing model, the performance metrics shown in Table 7 were evaluated. Analysis of the ML classifiers before hyperparameter tuning indicates that the RF and XGB classifiers achieved higher accuracies of 70.01% and 68.36%, respectively. However, the MNB method produced the lowest performance, with an accuracy of 51.53%.

**Table 7:** Performance comparison of six ML classifiers before and after hyperparameter tuning.

Model	Hyperparameter before Tuning				Hyperparameter after Tuning			
	Accuracy %	Precision %	Recall %	F1-Score %	Accuracy %	Precision %	Recall %	F1-Score %
NB	51.53	51	52	51	51.53	51	52	51
**RF**	**70.01**	**70**	**70**	**70**	**71.3**	**72**	**71**	**71**
DT	58.25	58	58	58%	58.74	59	59	59
SVM	57.73	58	58	58	69.57	70	70	70
KNN	66.34	66	66	66	67.43	68	67	66
XGBoost	68.36	70	68	68	70.49	71	70	70

**Fig 9:**
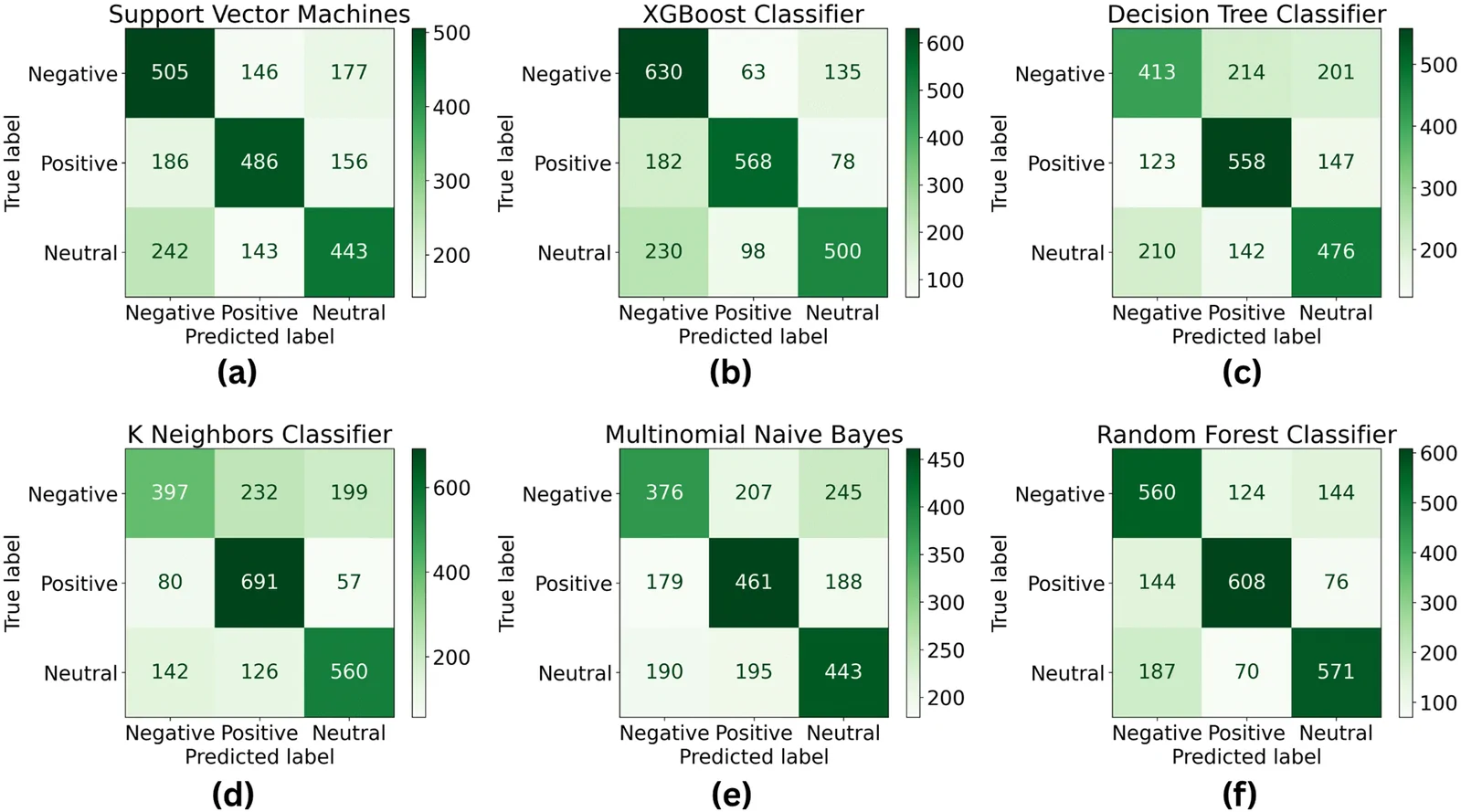
Confusion matrix of the ML methods before hyperparameter tuning.

For further investigation, the RandomizedSearchCV technique was used to obtain the optimal combination of Hyperparameters, thereby enhancing the model’s overall performance. Table 8 lists the most suitable Hyperparameter combinations along with the default combinations. In Fig 10, the confusion matrices of the employed ML classifiers after hyperparameter tuning for our Bangla dataset are presented. In particular, the KNN and SVM classifiers shown in Fig 10(d) and 10(a), respectively, demonstrate notable progress, in perfectly determining positive and neutral instances. In addition, the XGB and RF models are depicted in Fig 10(b) and 10(f), respectively, which continue to produce considerable outcomes, indicating a smaller number of classification errors across all categories. In Fig 10(c), the DT method presents a balanced outcome, whereas the MNB classifier shown in Fig 10(e) continues to have relatively low effectiveness, preserving identical patterns of miscategorization as before. For further clarification, the performance matrices of the utilized ML models after hyperparameter tuning are presented in Table 7. Among all the classifiers, the RF model achieved the highest results with an accuracy of 71.3%, followed closely by the XGBoost classifier (70.49%) and the SVM model (69.57%). These models also demonstrated substantial precision, recall, and F1-scores, all of which were approximately 70–72%. Conversely, the NB technique achieves the lowest result across all the models, with an accuracy of 51.53%. The DT and KNN methods yielded average outcomes, with the DT method achieving 58.74% accuracy and the KNN method attaining a minimum of 67.43%.

**Table 8:** Hyperparameter combination status of ML classifier before and after tuning.

Classifier	Hyperparameter before Tuning	Hyperparameter after Tuning
**NB**	‘alpha’: 1.0, ‘fit_prior’: True, ‘class_prior’: None	‘alpha’: 1.3, ‘fit_prior’: False, ‘class_prior’: 0.3
**RF**	‘criterion’: ‘gini’, ‘max_features’: ‘sqrt’, ‘n_estimators’: 100	‘criterion’: ‘entropy’, ‘max_features’: ‘log2’, ‘n_estimators’: 150
**DT**	‘criterion’: ‘gini’, ‘max_features’: None, ‘splitter’: ‘best’	‘criterion’: ‘entropy’, ‘max_features’: ‘sqrt’, ‘splitter’: ‘random’
**SVM**	‘C’: 1.0, ‘gamma’: ‘scale’, ‘kernel’: ‘rbf’	‘C’: 20, ‘gamma’: ‘auto’, ‘kernel’: ‘linear’
**KNN**	‘algorithm’: ‘auto’, ‘n_neighbors’: 5, ‘weights’: ‘uniform’	‘algorithm’: ‘ball_tree’, ‘n_neighbors’: 3, ‘weights’: ‘distance’
**XGBoost**	‘gamma’: 0’, ‘learning_rate’: 0.3, ‘n_estimators’: 100	‘gamma’: 0.01, ‘learning_rate’: 0.001, ‘n_estimators’: 500

**Fig 10:**
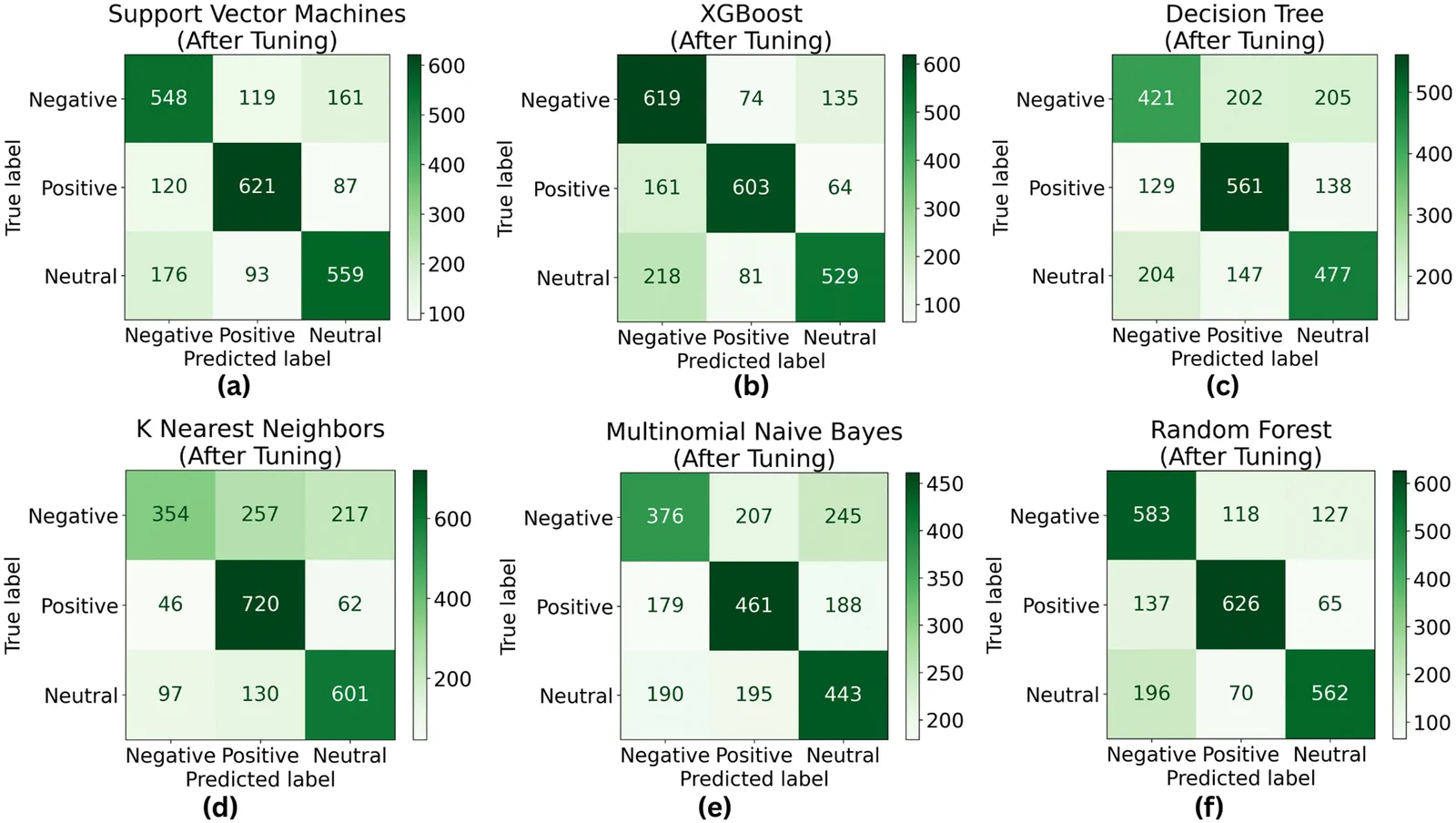
Confusion matrix of the ML methods after hyperparameter tuning.

As depicted in Table 7 and Fig 11, hyperparameter tuning had a positive effect on the performance of most classification models. For example, the SVM classifier exhibited the most significant progress, with its accuracy improving from 57.73% to 69.57%. Similarly, the RF and XGBoost classifiers also demonstrated performance improvements, with RF increasing from 70.01% to 71.3% and XGBoost increasing from 68.36% to 70.49%. In addition, the KNN and DT models demonstrated modest but reliable growth in accuracy after hyperparameter tuning. Conversely, the performance of NB remained constant, indicating that tuning had a negligible effect on its hyperparameters. Overall, both the table and the line chart demonstrate that hyperparameter tuning is notably effective for the applied classifiers, leading to improved overall performance.

**Fig 11:**
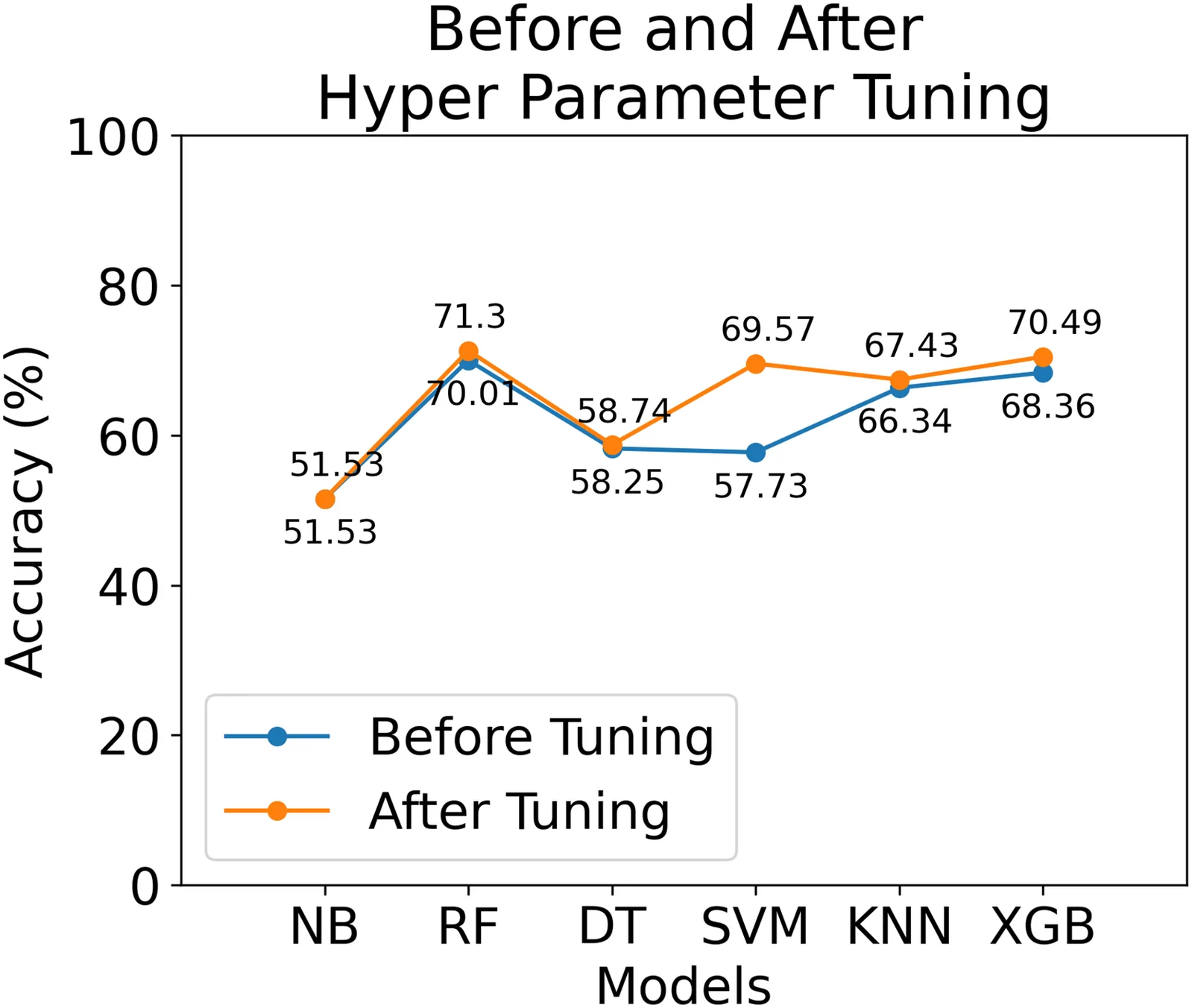
Accuracy comparison before and after hyperparameter tuning.

Table 9 highlights the impact of the TF-IDF approach on various machine learning models. Notably, the SVM classifier shows significant improvements across all metrics, with accuracy rising from 70.69% to 75%, accompanied by increases in precision, recall, and F1-score. The KNN model also demonstrates a significant improvement, particularly in precision and recall, resulting in a notable rise in its F1-score from 52.08% to 61.89%. In contrast, the Naive Bayes, Random Forest, and Decision Tree classifiers show minimal or no improvement after hyperparameter tuning while using the TF-IDF technique, with their performance metrics remaining essentially unchanged. The XGBoost model shows a slight performance decline, with accuracy decreasing from 62.04% to 61.23%, alongside small drops in other metrics. The best selected hyperparameters for all the applied ML models are shown in Table 10. Overall, the results underscore the varied effectiveness of hyperparameter tuning across different models, with some classifiers benefiting more than others.

**Table 9:** Performance comparison of TF-IDF classifiers before and after hyperparameter tuning (%).

Model	Accuracy Before	Precision Before	Recall Before	F1-Score Before	Accuracy After	Precision After	Recall After	F1-Score After	Improvement
Naive Bayes	58.82	59.24	58.82	58.90	58.94	59.21	58.94	59.00	0.0012
Random Forest	70.89	70.87	70.89	70.87	70.89	70.87	70.89	70.87	0.0
Decision Tree	65.26	65.19	65.26	65.20	65.26	65.19	65.26	65.20	0.0
SVM	70.69	71.57	70.69	70.61	75.00	75.08	75.00	74.91	0.0431
KNN	54.43	57.27	54.43	52.08	63.45	65.80	63.45	61.89	0.0902
XGBoost	62.04	63.11	62.04	62.15	61.23	62.39	61.23	61.26	−0.0081

**Table 10:** Best hyperparameters for each model and feature.

Feature	Model	Best Params
TF-IDF	Naive Bayes	{‘alpha’: 0.5}
	Random Forest	{‘max_depth’: None, ‘min_samples_split’: 2, ‘n_estimators’: 100}
	Decision Tree	{‘criterion’: ‘gini’, ‘max_depth’: None, ‘min_samples_split’: 2}
	SVM	{‘C’: 10, ‘kernel’: ‘rbf’}
	KNN	{‘n_neighbors’: 3, ‘weights’: ‘distance’}
	XGBoost	{‘learning_rate’: 0.3, ‘max_depth’: 5, ‘n_estimators’: 100}
POS Tagging	Naive Bayes	{‘alpha’: 0.5}
	Random Forest	{‘max_depth’: None, ‘min_samples_split’: 2, ‘n_estimators’: 100}
	Decision Tree	{‘criterion’: ‘gini’, ‘max_depth’: None, ‘min_samples_split’: 2}
	SVM	{‘C’: 10, ‘kernel’: ‘rbf’}
	KNN	{‘n_neighbors’: 3, ‘weights’: ‘distance’}
	XGBoost	{‘learning_rate’: 0.3, ‘max_depth’: 5, ‘n_estimators’: 100}
Unigram	Naive Bayes	{‘alpha’: 0.5}
	Random Forest	{‘max_depth’: None, ‘min_samples_split’: 2, ‘n_estimators’: 100}
	Decision Tree	{‘criterion’: ‘gini’, ‘max_depth’: None, ‘min_samples_split’: 2}
	SVM	{‘C’: 10, ‘kernel’: ‘rbf’}
	KNN	{‘n_neighbors’: 3, ‘weights’: ‘distance’}
	XGBoost	{‘learning_rate’: 0.3, ‘max_depth’: 5, ‘n_estimators’: 100}
Bigram	Naive Bayes	{‘alpha’: 0.5}
	Random Forest	{‘max_depth’: None, ‘min_samples_split’: 2, ‘n_estimators’: 100}
	Decision Tree	{‘criterion’: ‘gini’, ‘max_depth’: None, ‘min_samples_split’: 2}
	SVM	{‘C’: 10, ‘kernel’: ‘rbf’}
	KNN	{‘n_neighbors’: 3, ‘weights’: ‘distance’}
	XGBoost	{‘learning_rate’: 0.3, ‘max_depth’: 5, ‘n_estimators’: 100}
Trigram	Naive Bayes	{‘alpha’: 1.0}
	Random Forest	{‘max_depth’: None, ‘min_samples_split’: 2, ‘n_estimators’: 100}
	Decision Tree	{‘criterion’: ‘gini’, ‘max_depth’: None, ‘min_samples_split’: 2}
	SVM	{‘C’: 1, ‘kernel’: ‘rbf’}
	KNN	{‘n_neighbors’: 3, ‘weights’: ‘distance’}
	XGBoost	{‘learning_rate’: 0.3, ‘max_depth’: 5, ‘n_estimators’: 100}

In addition to accuracy, the comparable precision, recall, and F1-score values across all major classifiers indicate that the models maintain balanced performance across sentiment classes and are not substantially affected by class imbalance.

The comparison of POS tagging classifier performance before and after hyperparameter tuning is shown in Table 11. The results indicate varying impacts of using the POS tagger across different models. The Naive Bayes classifier shows a slight improvement, with accuracy increasing from 60.75% to 61.07%, along with small gains in precision, recall, and F1-score. Similarly, the Support Vector Machine (SVM) model demonstrates a modest improvement, with a slight increase in accuracy from 77.94% to 78.18%, and corresponding improvements in precision, recall, and F1-score. The Random Forest and Decision Tree models, however, show no change in their performance metrics after hyperparameter tuning, maintaining consistent results across all measures. The K-Nearest Neighbors (KNN) model shows significant improvement, particularly in precision and recall, resulting in a notable increase in its F1-score from 48.96% to 63.97%. On the other hand, the XGBoost model shows a small improvement, with a slight increase in its F1-score from 72.94% to 73.11%, though its accuracy remains almost unchanged. Table 10 presents the selected hyperparameters for each model when POS tagging is used.

**Table 11:** Performance comparison of POS tagging classifiers before and after hyperparameter tuning (%).

Model	Accuracy Before	Precision Before	Recall Before	F1-Score Before	Accuracy After	Precision After	Recall After	F1-Score After	Improvement
Naive Bayes	60.75	63.43	60.75	60.95	61.07	63.90	61.07	61.30	0.0032
Random Forest	77.25	78.17	77.25	77.44	77.25	78.17	77.25	77.44	0.0
Decision Tree	64.73	64.70	64.73	64.68	64.73	64.70	64.73	64.68	0.0
SVM	77.94	80.07	77.94	78.21	78.18	79.08	78.18	78.38	0.0024
KNN	55.56	71.43	55.56	48.96	67.03	75.21	67.03	63.97	0.1147
XGBoost	72.75	73.84	72.75	72.94	72.91	74.15	72.91	73.11	0.0016

Table 12 compares the performance of unigram classifiers before and after hyperparameter tuning, using metrics such as accuracy, precision, recall, and F1-score. The results show that the SVM model significantly improves accuracy, increasing from 62.96% to 65.06%, accompanied by gains in precision, recall, and F1-score. The K-Nearest Neighbors (KNN) model also reveals substantial progress, particularly in recall and F1-score, with accuracy rising from 51.29% to 57.53%. However, the Naive Bayes, Random Forest, and Decision Tree models exhibit minimal to no advancement, with performance metrics remaining essentially unchanged. The XGBoost model, in contrast, shows a decline in performance across all metrics, with accuracy dropping from 42.87% to 40.82% and the F1-score decreasing accordingly. The best hyperparameters of each classifier are displayed in Table 10. These results highlight the varying effectiveness of the unigram approach across different models.

**Table 12:** Performance comparison of unigram classifiers before and after hyperparameter tuning (%).

Model	Accuracy Before	Precision Before	Recall Before	F1-Score Before	Accuracy After	Precision After	Recall After	F1-Score After	Improvement
Naive Bayes	53.62	54.54	53.62	53.63	53.74	54.52	53.74	53.76	0.0012
Random Forest	64.05	64.04	64.05	63.94	64.05	64.04	64.05	63.94	0.0
Decision Tree	61.92	61.96	61.92	61.62	61.92	61.96	61.92	61.62	0.0
SVM	62.96	63.19	62.96	62.78	65.06	65.14	65.06	64.98	0.0209
KNN	51.29	53.54	51.29	49.57	57.53	58.98	57.53	56.87	0.0624
XGBoost	42.87	57.92	42.87	35.91	40.82	60.15	40.82	32.99	−0.0205

Table 13 presents the performance comparison of bigram classifiers before and after hyperparameter tuning. The results indicate no improvement in performance for the Naive Bayes, Random Forest, Decision Tree, and Support Vector Machine (SVM) models, as their metrics remain unchanged across all measures. However, the K-Nearest Neighbors (KNN) model shows a slight decrease in performance, with accuracy dropping from 50.81% to 50.16% and the F1-score decreasing slightly. Similarly, the XGBoost model also exhibits no change in its performance metrics. These results suggest that hyperparameter tuning had minimal impact on most models when using the bigram approach, with the KNN and XGBoost models experiencing slight declines. Different hyperparameters were selected for each model, as shown in Table 10.

**Table 13:** Performance comparison of bigram classifiers before and after hyperparameter tuning (%).

Model	Accuracy Before	Precision Before	Recall Before	F1-Score Before	Accuracy After	Precision After	Recall After	F1-Score After	Improvement
Naive Bayes	49.96	54.09	49.96	49.63	49.96	54.09	49.96	49.63	0.0
Random Forest	57.85	60.28	57.85	57.01	57.85	60.28	57.85	57.01	0.0
Decision Tree	57.37	60.44	57.37	56.32	57.37	60.44	57.37	56.32	0.0
SVM	59.46	61.93	59.46	58.46	59.46	61.93	59.46	58.46	0.0
KNN	50.81	56.80	50.81	48.57	50.16	54.32	50.16	50.30	−0.0064
XGBoost	37.32	43.06	37.32	24.40	37.32	43.06	37.32	24.40	0.0

The performance comparison of trigram classifiers before and after hyperparameter tuning is shown in Table 14. The K-Nearest Neighbors (KNN) model demonstrates a significant improvement, with a notable increase in accuracy from 42.83% to 48.63% and substantial increases in precision and recall. In contrast, the results show no improvement for the Naive Bayes, Random Forest, and XGBoost models, as their performance metrics remain unchanged. The Decision Tree and SVM models experience a slight decrease in performance after tuning, with accuracy and F1-score dropping slightly. Table 10 displays the best selected hyperparameters for all the applied models. Overall, the results suggest that hyperparameter tuning had a limited impact across most models, with the KNN classifier benefiting the most from the trigram approach.

**Table 14:** Performance comparison of trigram classifiers before and after hyperparameter tuning (%).

Model	Accuracy Before	Precision Before	Recall Before	F1-Score Before	Accuracy After	Precision After	Recall After	F1-Score After	Improvement
Naive Bayes	47.10	58.79	47.10	44.82	47.10	58.79	47.10	44.82	0.0
Random Forest	50.16	62.69	50.16	47.09	50.16	62.69	50.16	47.09	0.0
Decision Tree	49.64	63.73	49.64	46.37	49.56	63.62	49.56	46.29	−0.0008
SVM	50.81	62.98	50.81	47.98	48.03	62.83	48.03	45.25	−0.0278
KNN	42.83	59.62	42.83	37.01	48.63	65.88	48.63	45.65	0.058
XGBoost	39.49	69.16	39.49	29.14	39.49	68.70	39.49	29.15	0.0

Fig 12 delineates the performance of six hyperparameter-tuned classifiers employing TF-IDF vectorization, illustrating the impact of statistical term weighting on Bengali sentiment classification. The Support Vector Machine (SVM) emerges as a robust contender, securing the highest accuracy for negative (619) and neutral (686) sentiments, thereby demonstrating superior class-wise discrimination. Conversely, while the KNN model excels at identifying neutral instances (693), it shows a pronounced deficiency in negative class sensitivity, with only 310 correct labels. Random Forest and XGBoost also display competitive performance, though the former achieves a more balanced diagonal distribution across all the polarities.

**Fig 12:**
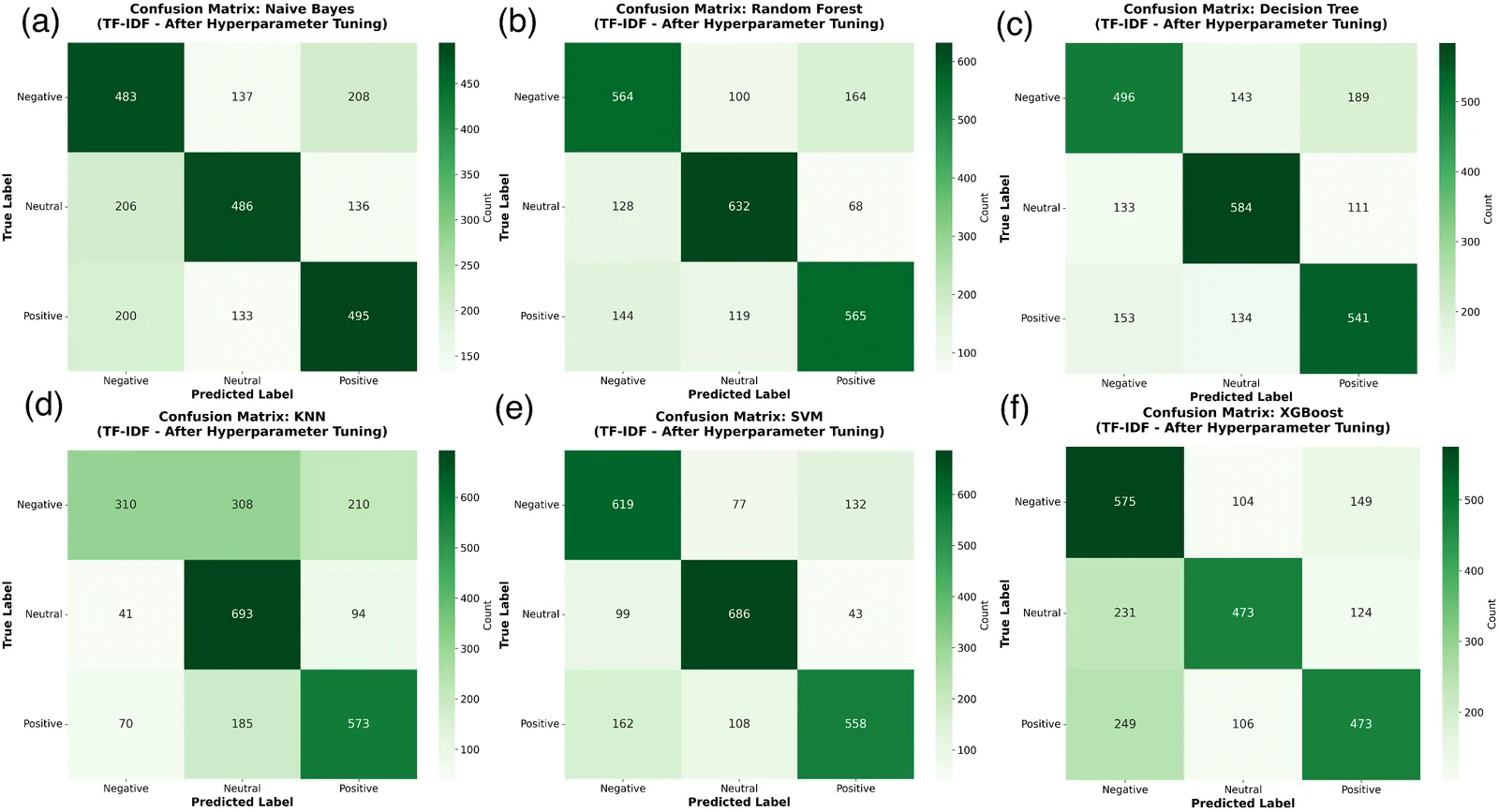
Confusion matrix of applied ML model (Applying TF_IDF).

The evaluative outcomes for hyperparameter-tuned classifiers integrated with Part-of-Speech (POS) tagging are visualized in Fig 13, illustrating a refined capacity for sentiment discrimination in the Bengali corpus. Both the Support Vector Machine (d) and Random Forest (b) demonstrated exceptional robustness, with SVM achieving a high frequency of 661 and 629 correct predictions for negative and positive classes, respectively. Conversely, the KNN model (e) displayed a significant class imbalance, accurately identifying 770 positive instances but failing to capture the negative spectrum effectively, with only 214 successful classifications.

**Fig 13:**
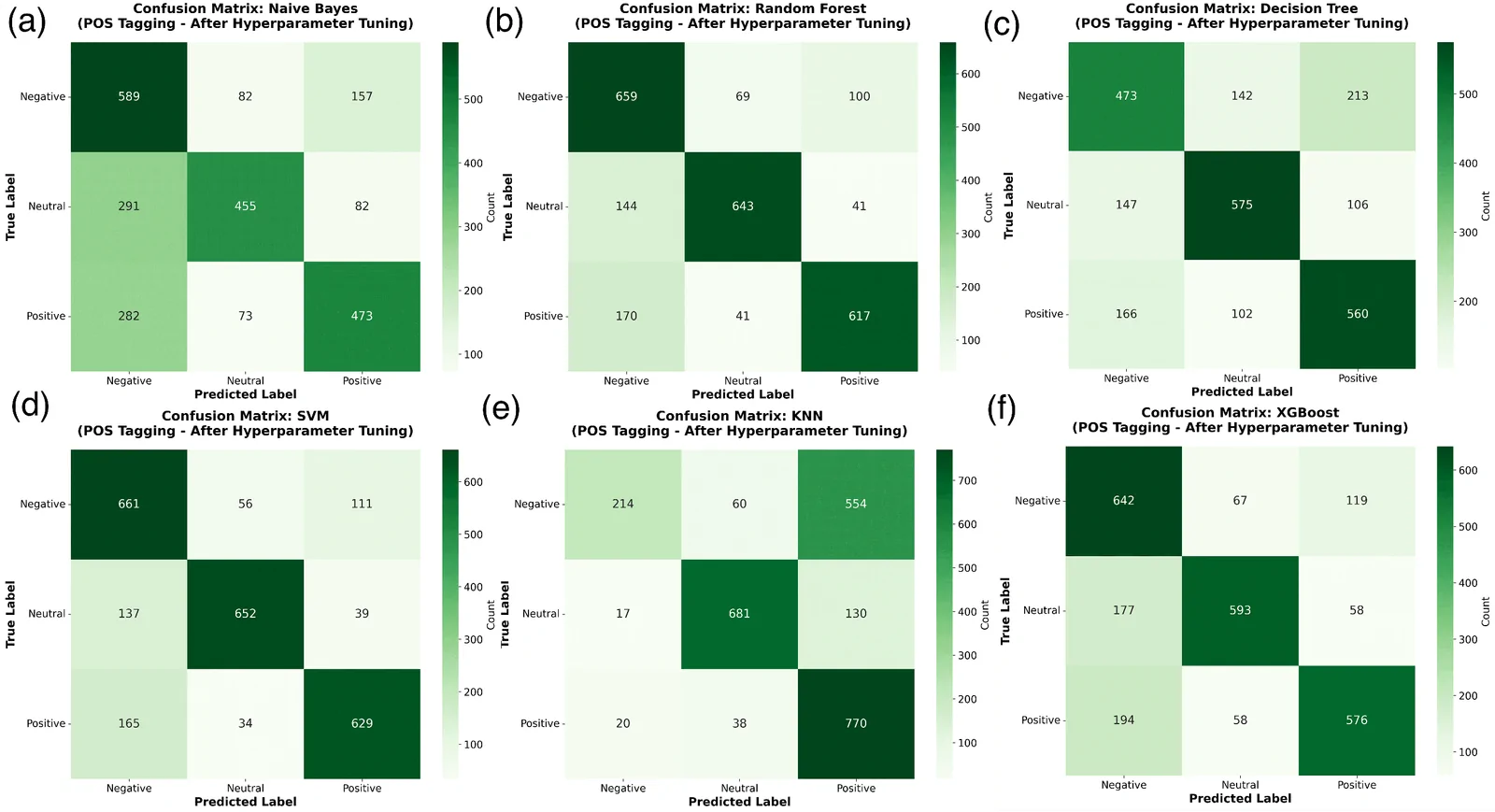
Confusion matrix of applied ML model (Applying POS tagging).

The classification efficacy of the unigram-based models is detailed in Fig 14, where the confusion matrices reveal distinct behavioral trends across the six hyperparameter-tuned architectures. The Support Vector Machine (d) and Decision Tree (c) emerge as the most robust classifiers within this feature set, with the SVM accurately labeling 566 negative instances and the Decision Tree achieving a peak of 612 correct neutral identifications. While Naive Bayes (a) and Random Forest (b) maintain relatively balanced diagonal distributions, they both exhibit significant overlapping errors between polarized sentiments and the neutral class. Conversely, KNN (e) demonstrates a marked deficiency in negative class sensitivity, capturing only 334 instances, whereas XGBoost (f) exhibits a near-total predictive collapse, disproportionately assigning the majority of samples to the positive class.

**Fig 14:**
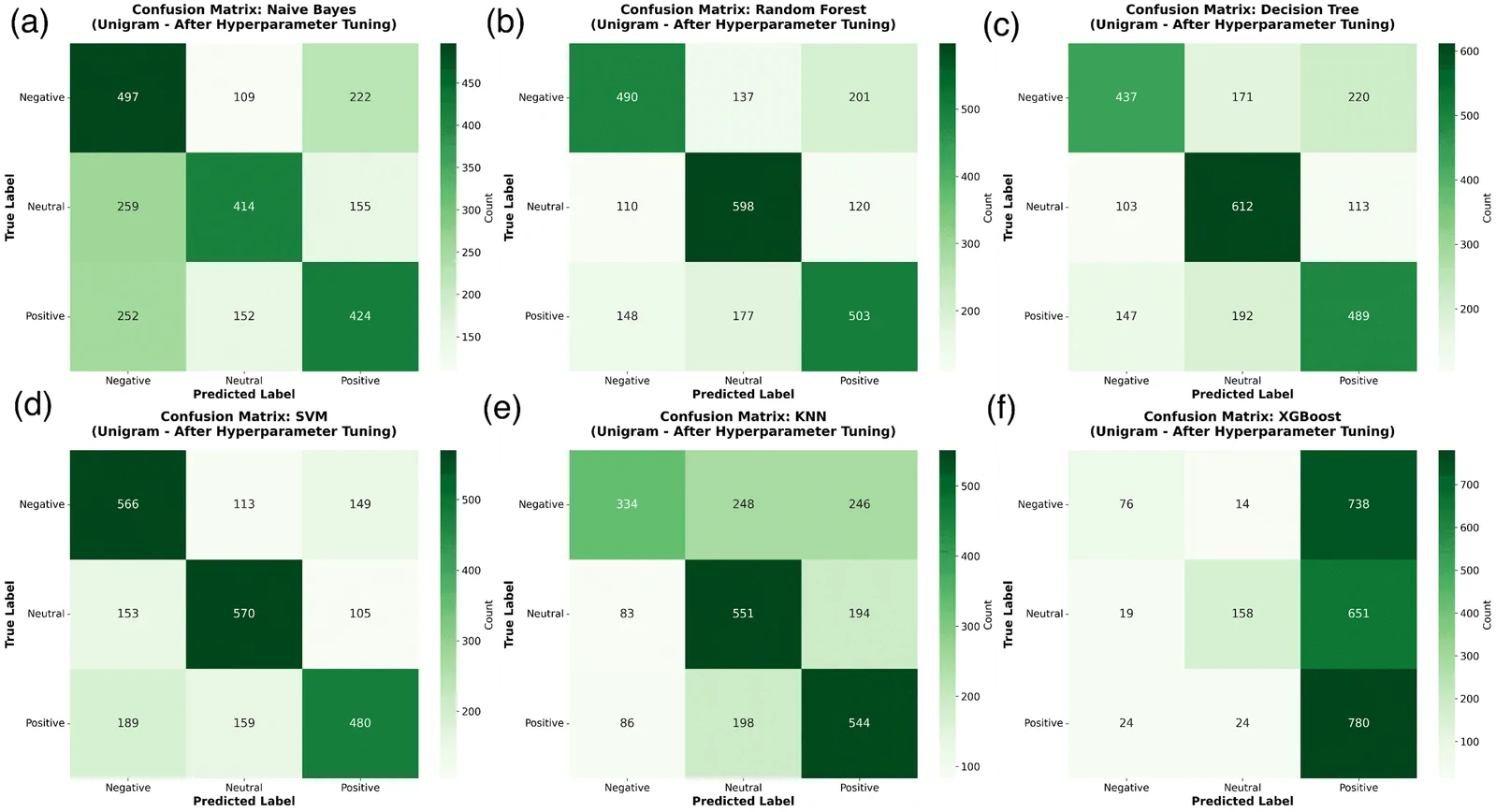
Confusion matrix of applied ML model (Applying unigram).

To estimate the predictive reliability of the optimized machine learning classifiers, Fig 15 demonstrates a comparative analysis using confusion matrices for six distinct models that utilize bigram feature sets. The results reveal varied class-wise discrimination capabilities; for instance, NB (a) and KNN (e) demonstrate the highest sensitivity toward negative sentiments, accurately recognizing 565 and 529 cases, respectively. In contrast, the tree-based architectures, specifically RF (b) and DT (c), show outstanding performance in classifying neutral sentiment, correctly labeling over 660 samples. While the Support Vector Machine (d) maintains a more equitable distribution of predictions across the three classes, the XGBoost model (f) shows a significant classification bias toward the positive class, failing to differentiate between negative and neutral polarities under this specific bigram configuration.

**Fig 15:**
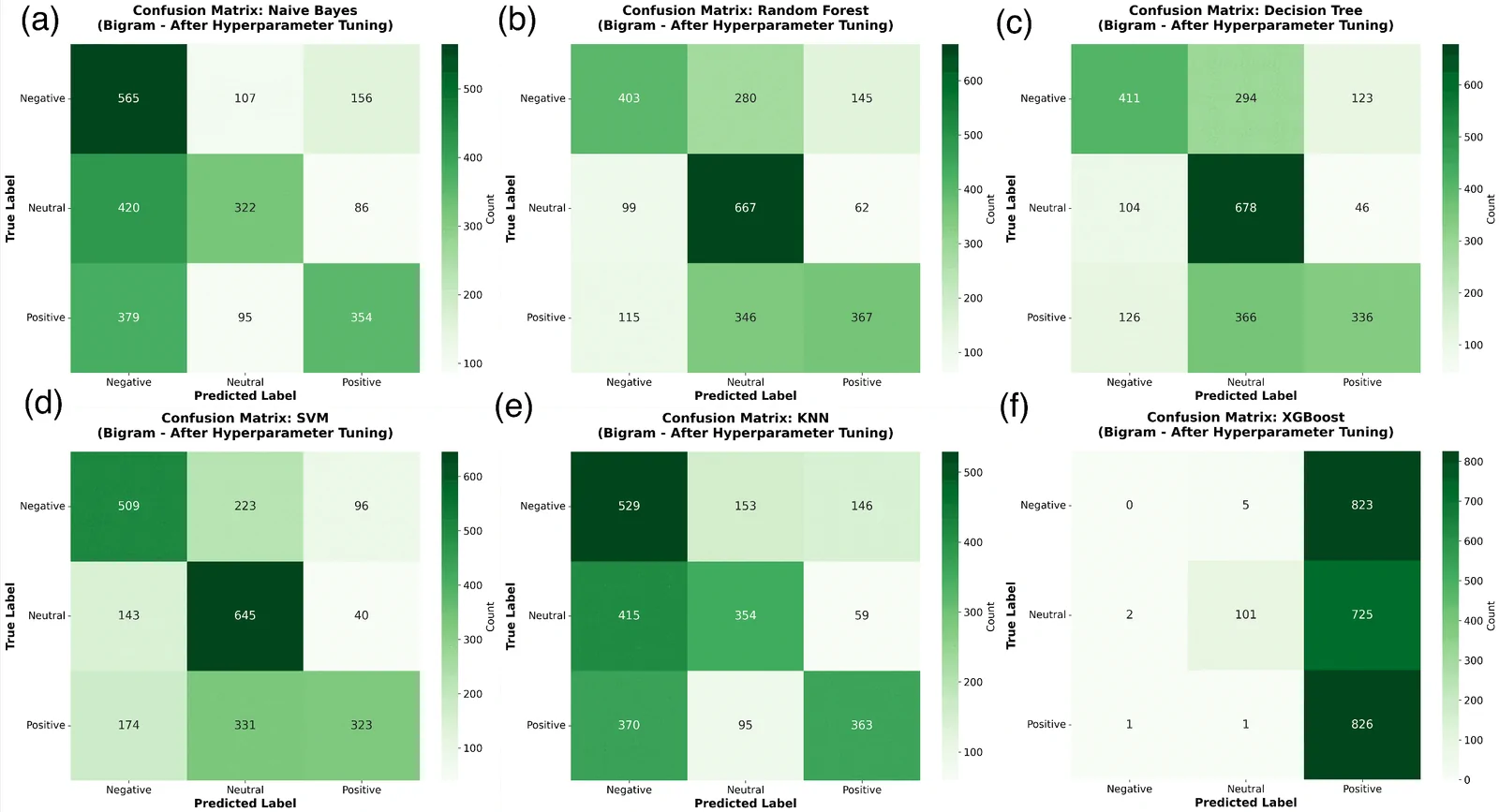
Confusion matrix of applied ML model (Applying bigram).

The performance of the six tuned classifiers utilizing trigram feature extraction is detailed across Fig 16, revealing a systemic difficulty in maintaining class-wise equilibrium. Models such as Naive Bayes (a), SVM (d), and KNN (e) demonstrate high sensitivity for the negative class–-correctly identifying 688, 728, and 749 instances respectively–-but they suffer from a severe tendency to misclassify neutral and positive sentiments as negative. In contrast, the tree-based architectures, Random Forest (b) and Decision Tree (c), shift this predictive bias toward the neutral class, yielding high correct labels for neutral samples (over 750) while failing to capture polarized expressions. The most pronounced imbalance is evident in XGBoost (f), which exhibits a near-total collapse in discrimination, defaulting almost exclusively to pessimistic predictions.

**Fig 16:**
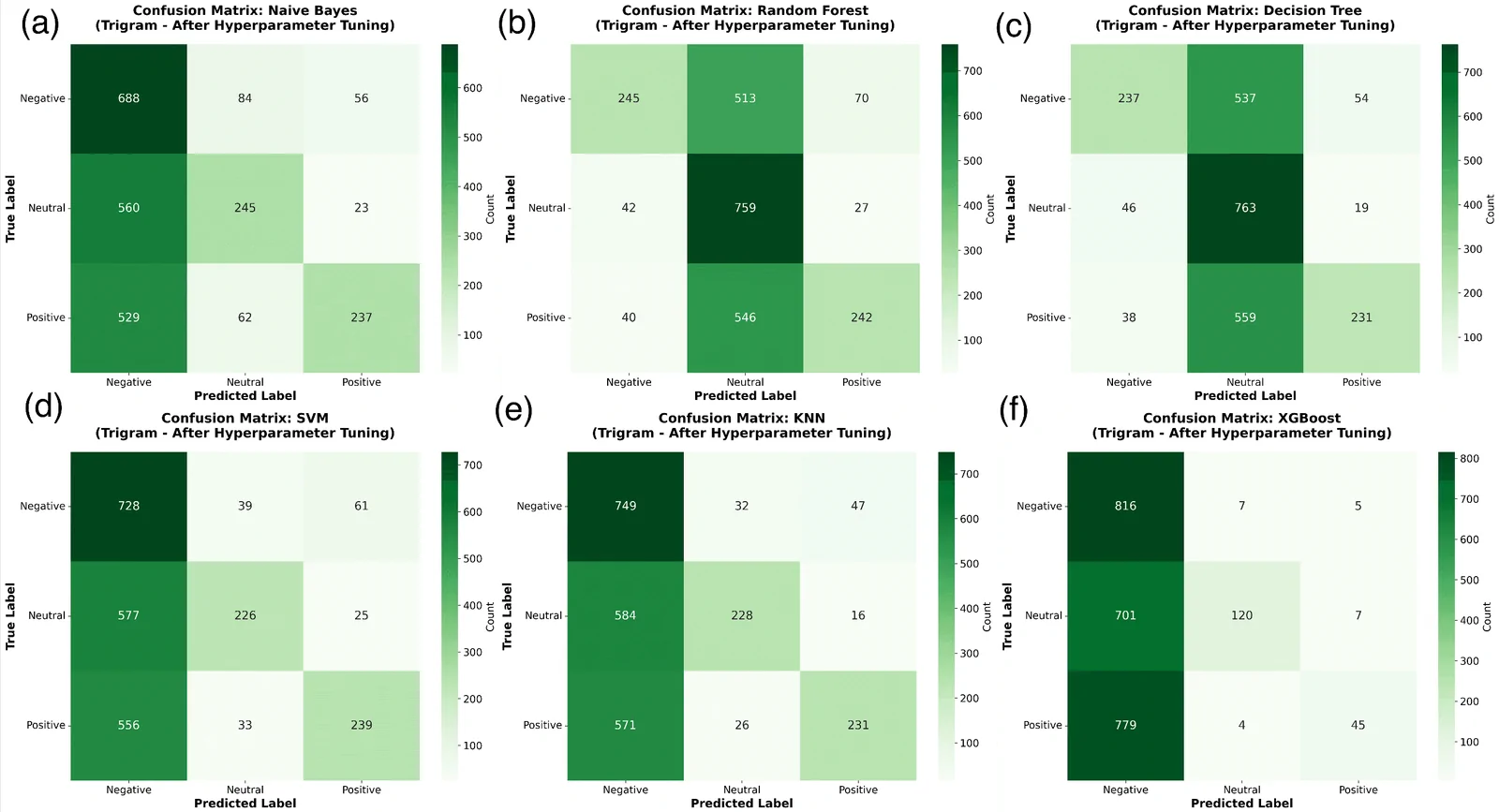
Confusion matrix of applied ML model (Applying trigram).

### Performance of deep learning models

In this study, we introduced several advanced technologies, such as deep learning models, to achieve much better outcomes. The deep GRU and LSTM models were employed for further investigation, where the performance evaluation is shown in Fig 17. The confusion matrix of the deep GRU model depicted in Fig 17(a), yields significant classification results on the test dataset. This model perfectly identifies 519 negative, 603 positive, and 546 neutral sentiments, with comparatively low misclassification among classes. For example, most confusions occurs between neutral and negative sentiments, however, overall class categorization is carefully maintained. Fig 17(c) shows the accuracy growth over 120 epochs, where the training accuracy significantly increases and approaches 98%, while the test accuracy becomes steady approximately 70%. This pattern suggests effective learning, although the growing gap between the training and test curves suggests some degree of overfitting. This model achieved an accuracy of 67.63% as shown in Table 15, which is suitable for a sustainable approach. In comparison, the LSTM model demonstrates slightly lower performance, as illustrated in the confusion matrix shown in Fig 17(b). This model precisely recognizes 479 negative, 501 positive, and 422 neutral classes, but portrays more misclassifications, especially in terms of neutral sentiments, where 295 samples are incorrectly classified as negative. Fig 17(d) shows a slower increase in accuracy across 140 epochs, with the training accuracy maximizing approximately 74% and the test accuracy stabilizing approximately 57%. The more comprehensive gap between training and test performance indicates limited generalization ability and presents a greater susceptibility to overfitting. Moreover, this model achieved a notably lower accuracy of 56.28%, as shown in Table 15. Overall, the GRU model achieved better recognition among the classes and achieved higher test accuracy more quickly. Although both models exhibit signs of overfitting, the GRU maintains a better balance between training and test performance, indicating its superior effectiveness in capturing and generalizing sentiment patterns from the data. In contrast, when deep learning models were employed in the study, consistent performance was not observed.

**Table 15:** Performance evaluation of the GRU, LSTM, BERT, GPT2_BENGALI and ensemble models.

Model	Accuracy%	Precision%	Recall%	F1-Score%
Ensemble	78.26	79	77	78
GRU + word2vec	67.63	67	67	67
LSTM + word2vec	56.28	57	56	57
GRU + fasttext	58.13	59	58	58
LSTM + fasttext	57.25	57	57	57
GPT2_BENGALI	66.10	66	66	65
BERT	80.11	80	80	80

**Fig 17:**
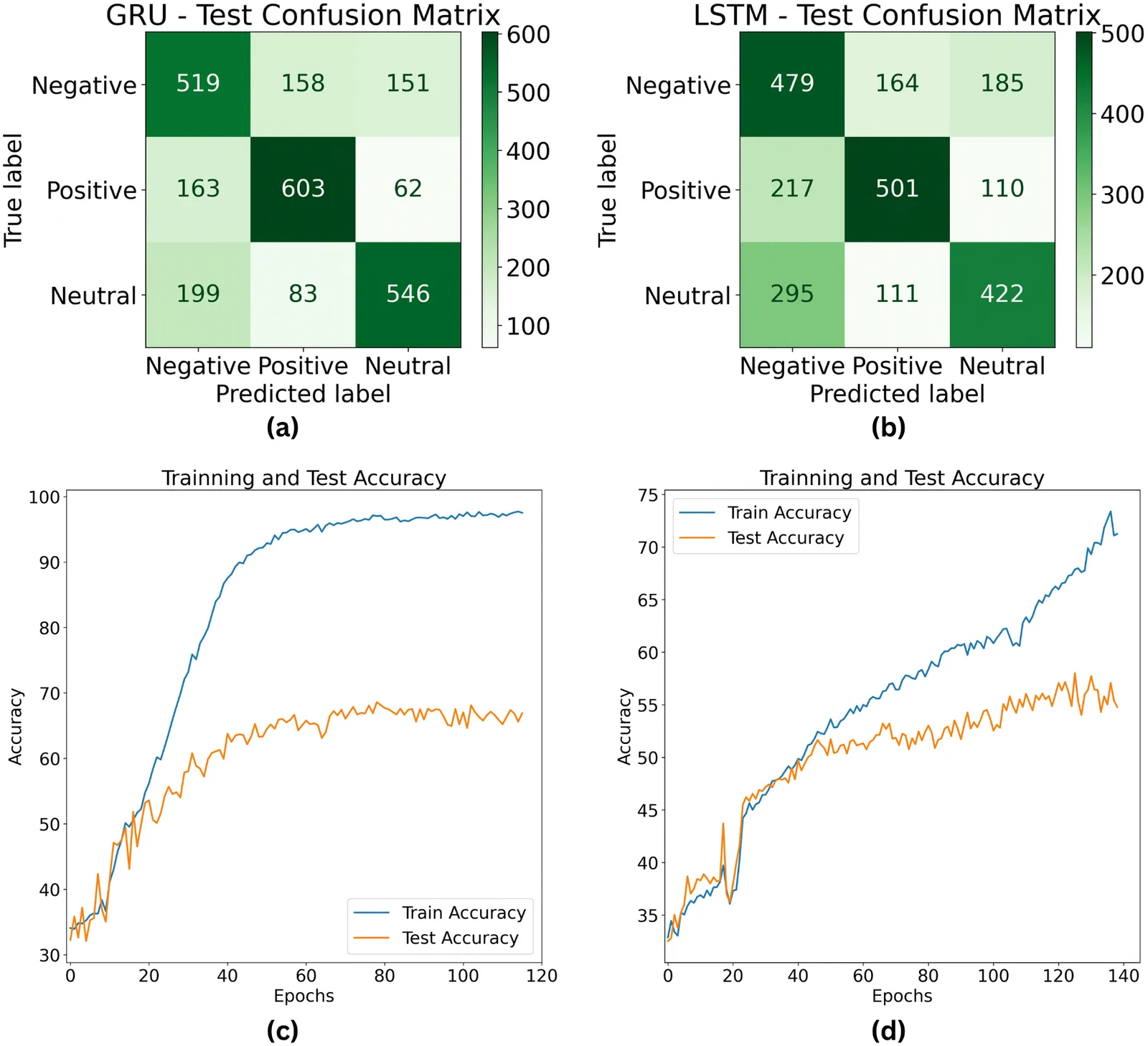
Performance evaluation of deep learning models.

The performance metrics for the Gated Recurrent Unit (GRU) and Long Short-Term Memory (LSTM) architectures, both using FastText word embeddings, are summarized in Fig 18, highlighting the models’ varying capacities for Bengali sentiment classification. The confusion matrices reveal that while the LSTM achieves higher sensitivity for the negative class (543 correct predictions), the GRU shows a more equitable distribution of accuracy, but a significant misclassification rate of 253 negative instances as positive. Notably, the accuracy trajectories (Fig 18c and 18d) display a noticeable generalization gap: as training accuracy converges toward 80%, validation performance flattens between 55% and 60%. This consistent divergence across both recurrent frameworks suggests that while deep learning captures local dependencies, the linguistic complexity of Bangla necessitates more advanced regularization or attention mechanisms to bridge the gap between training and real-world inference.

**Fig 18:**
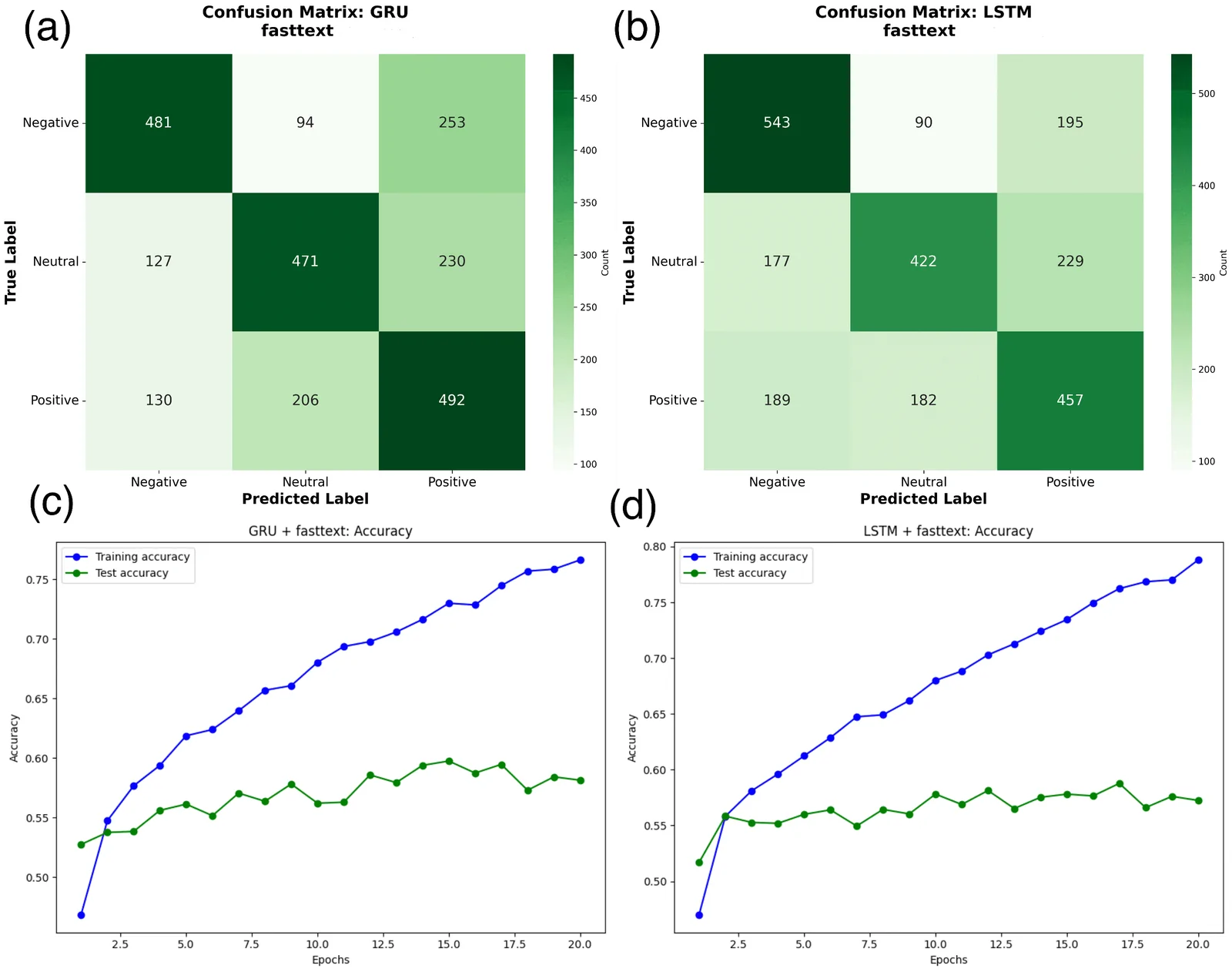
Performance of GRU + fasttext and LSTM + fasttext.

### Performance of the ensemble method

To enhance overall performance, the ensemble technique depicted in Fig 19 was introduced. The ensemble model utilizing a stacking technique demonstrated robust performance across numerous evaluation metrics. As depicted in Fig 19(a), the confusion matrix indicates better classification accuracy for all sentiment classes, with the method accurately recognizing 665 negative, 305 positive, and 463 neutral instances. There were some misclassifications that were more prevalent between the negative and neutral sentiments, which is anticipated due to the lexical proximity in some circumstances. The ROC curves in Fig 19(d) further validate the model’s performance. The AUC scores are 0.86 for the negative class, 0.89 for the positive class, and 0.91 for the neutral class, indicating high recognition power across all categories. Among these, the model performs best in determining the neutral class. In addition, the training and test loss directions (Fig 19(b)) reveal that the method converges promptly during the early epochs, with the training loss declining consistently to below 0.4. Nevertheless, the test loss achieves a stability of approximately 0.65. Nevertheless, the accuracy plot (Fig 19(c)) illustrates that the test accuracy maintains a relatively constant state at approximately 75%, whereas the training accuracy surpasses 85%, suggesting that the model exhibits satisfactory generalizability to unseen data. Furthermore, as demonstrated in Table 15, the method obtained a high accuracy of 78.26%, where the precision, recall, and F1-score were 79%, 77%, and 78%, respectively. Overall, the stacking ensemble method delivers a trustworthy and strong classification framework, with high sensitivity and generalizability across sentiment categories.

**Fig 19:**
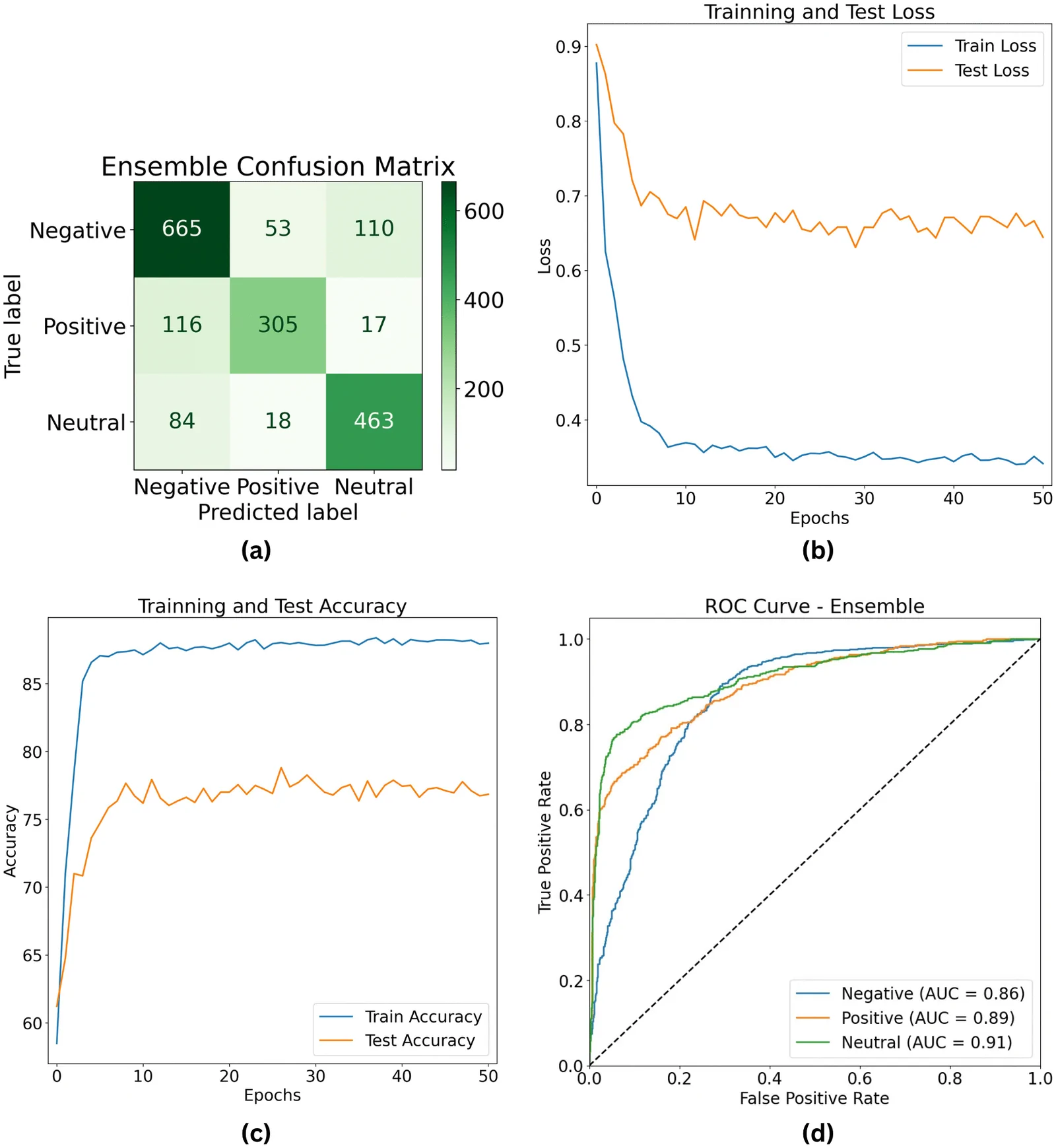
Performance evaluation for ensemble method (stacking).

### Performance of the transformer-based approach

In this subsection, a transformer-based deep learning approach, specifically BanglaBERT–-a pre-trained model that plays a significant role in Bangla Natural Language Processing–-was applied to enhance the performance of Bangla sentiment analysis. Fig 20 presents a detailed evaluation of the Bangla-BERT model across numerous performance metrics. In Fig 20(a), the confusion matrix emphasizes the model’s powerful classification capability across three sentiment classes–-negative, positive, and neutral. Remarkably, the model correctly identified 641 negative, 701 positive, and 648 neutral sentiments, indicating a well-balanced performance with comparatively fewer misclassifications. Fig 20(d) shows the ROC curves for the three sentiment categories. The model achieved significant AUC scores of 0.90 for negative, 0.95 for positive, and 0.91 for neutral, which supports its strong categorical strength across all sentiment classes.

**Fig 20:**
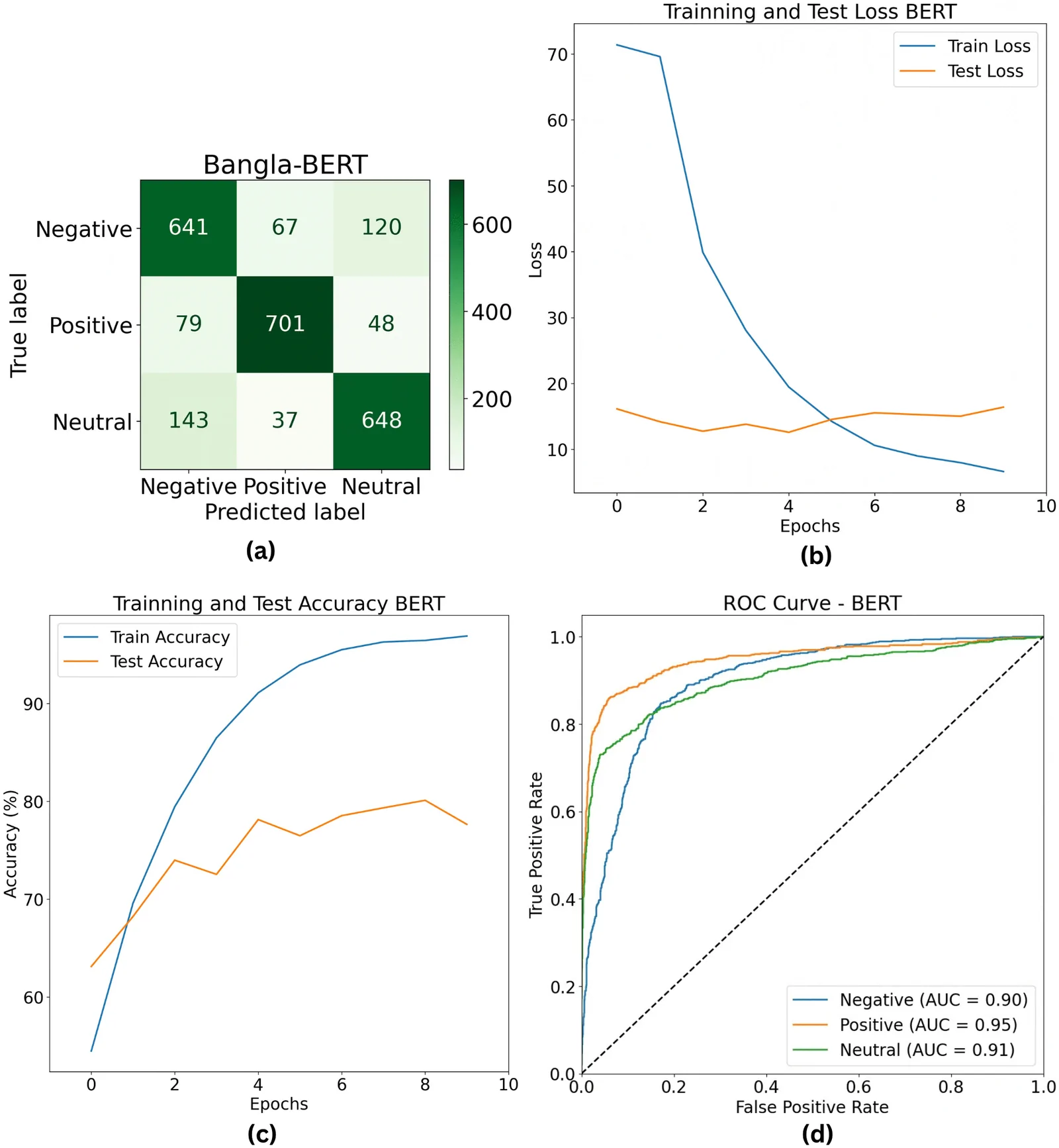
Performance evaluation of the BanglaBERT model.

In addition, to validate the best-performing model, training and testing loss and accuracy curves were generated. The training and testing loss curves over 10 epochs of this model are illustrated in Fig 20(b). A significant reduction in training loss is recorded, revealing well-generalized learning during training. Concurrently, the test loss remains consistently low, showing limited fluctuation, which suggests that the model does not overfit and maintains strong generalizability. Finally, Fig 20(c) depicts the training and test accuracy of this model. The training accuracy greatly increased, exceeding 95%, while the test accuracy also increased, reaching a maximum near 80%. This finding suggests that the model exhibits both effective learning and strong generalization capabilities on unobserved data. Table 15 shows that the BanglaBERT model achieves the highest accuracy of 80.11%, with precision, recall, and F1-score all at 80%, indicating the strong overall performance of the proposed approach.

Among all the employed models, BanglaBERT achieves high accuracy with strong generalizability and balanced class prediction. The stacking ensemble method also delivers trustworthy performance, notably exhibiting a strong ability to detect neutral sentiment. The GRU outperformed the LSTM among the deep learning models, although both demonstrated overfitting behavior. Conventional ML methods such as RF and XGBoost delivered positive outcomes post-tuning but demonstrated comparatively lower performance than the transformer-based model did. Overall, BanglaBERT has emerged as the most robust approach for Bangla sentiment classification.

The performance evaluation of the fine-tuned GPT2_BENGALI model is detailed in Fig 21, which illustrates the model’s classification performance and learning behavior across several diagnostic metrics. According to the confusion matrix (a), the architecture demonstrates a robust proficiency in identifying negative sentiments, correctly classifying 673 instances. However, it encounters greater difficulty with the positive class, where a significant portion of the data is misclassified into the neutral category. This struggle with positive sentiment is further evident in the ROC curves in panel (d), where Class Positive yields the lowest area under the curve (AUC) of 0.77, compared to the superior 0.88 for the negative class. Furthermore, the training curve in the accuracy (b) and loss (c) plots reveals a widening generalization gap after the initial epochs. In contrast, training performance continues to improve towards 90% accuracy, the test metrics plateau near 65%, suggesting that while the transformer-based model captures complex linguistic patterns in Bangla, it remains susceptible to overfitting. Collectively, these results, underscored by a micro-average ROC area of 0.84, indicate that GPT2 provides a sophisticated baseline for Bengali NLP.

**Fig 21:**
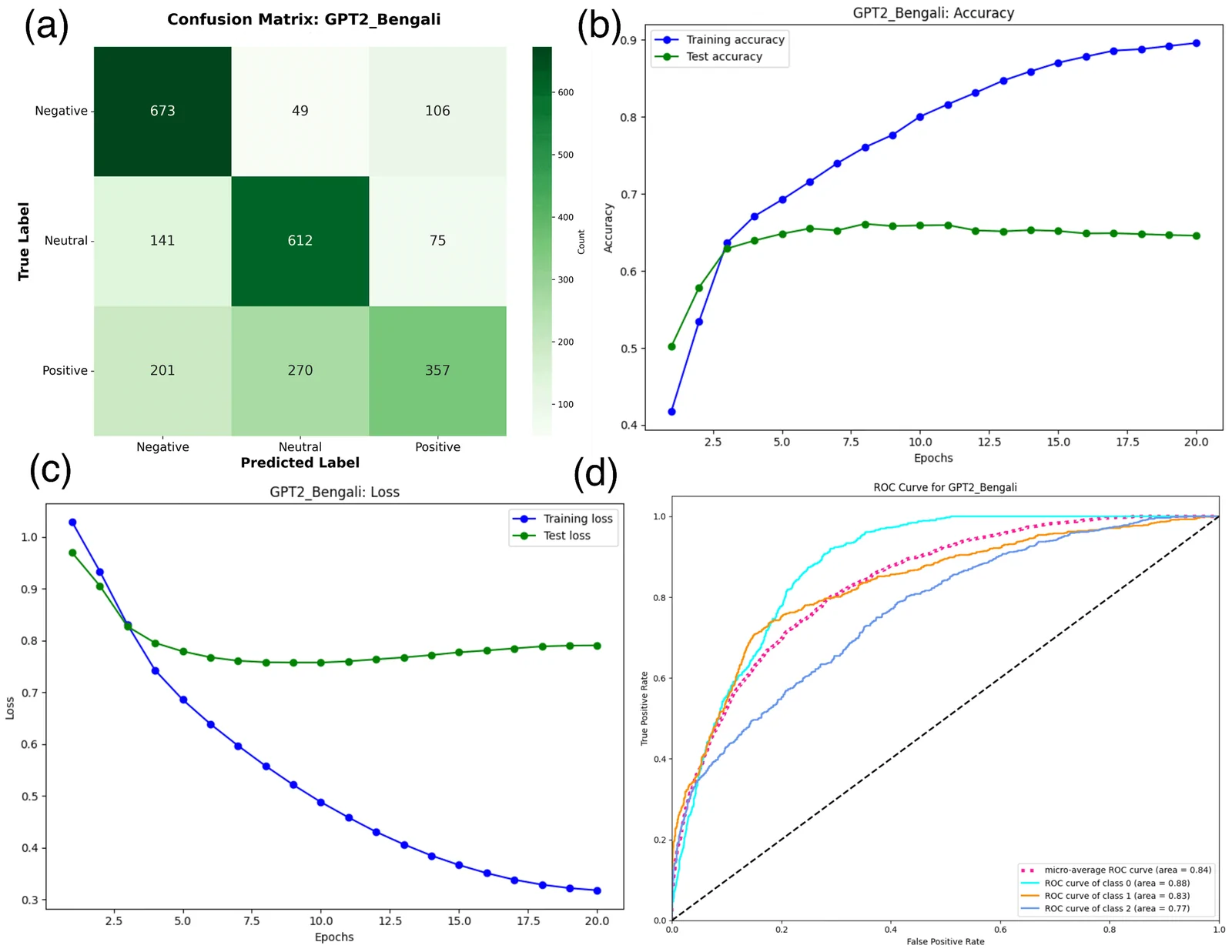
Performance of GPT2_BENGALI.

### Comparative overview with existing research

This subsection presents an overview of existing research efforts in Bangla sentiment analysis alongside the proposed system, as summarized in Table 16. It is important to note that the reported results should not be interpreted as a direct or fair quantitative comparison, since each study employs different datasets, domains, class distributions, and evaluation settings. Unlike many earlier studies that utilized relatively smaller datasets–-such as Islam et al. [38] with 1,002 samples and Hassan et al. [12] with 1,141 instances—this study incorporates a significantly larger and more diverse dataset comprising 9,163 user comments collected from multiple online sources. This enhanced dataset permits an improved and broadly applicable sentiment analysis model. Furthermore, while several previous studies utilized either machine learning or deep learning methods separately, the proposed approach is distinguished by integrating traditional ML algorithms (SVM, DT, RF, KNN, MNB) with deep learning models (GRU, LSTM) through an ensemble stacking technique. Additionally, BanglaBERT, a transformer-based model fine-tuned for the Bangla language, was incorporated and achieved the highest performance with an accuracy of 80.11%. Although some domain-specific systems, such as Sunny et al. [11] and Biswas et al. [35], achieved higher accuracies (96.42% and 92.86%, respectively), their models satisfied domain consistency and smaller, concise datasets. The primary contribution of this work lies in its multi-domain adaptability, hybrid modeling strategy, and applicability to real-world Bangla user-generated content rather than achieving the highest accuracy on controlled datasets.

**Table 16:** Comparison with existing methods.

Reference	Domain	Size of the Dataset	Methods	Accuracy / F1-score
Rahman et al. [10]	E-learning reviews from YouTube	6268 (Bangla+Romanized)	NB, SVM, RF, ANN, CNN, BiLSTM, BERT variants, Ensembles	85.81%
Sunny et al. [11]	Product Reviews	5,016	**MLP, SVM, RF, NB, KNN, ANN**	96.42%
Hassan et al. [12]	Conversation Reviews (Film/Script)	1,141	**SVM, NB, LR, KNN, DT, RF, SGD**	85.59%
Mia et al. [16]	Music Lyrics	2,152	NB, SVM, SGD, XGBoost, MLP, BLSTM, BERT	65%
Alvi et al. [20]	General Sentiment	7,000	**GRU**	78.41%
Rahman et al. [22]	Sports News	2,492	**CNN, MLP, LSTM**	75.57% (2-class), 48.19% (5-class)
Sharmin et al. [26]	News Comments	2,979	Attention-based CNN	72.06%
Uddin et al. [30]	Depression Analysis	5,000	**GRU**	75.7%
Biswas et al. [35]	Food Delivery Services	5,680	**BERT, Char-CNN, Graph-CNN, LSTM, BiLSTM**	92.86%
Islam et al. [38]	Newspaper Comments	1,002	BERT, GRU	71%
**Our Proposed System**	**User Comments from Online Platforms**	**9163**	SVM, DT, XGBoost, KNN, MNB, RF, GRU, LSTM, Stacking (ML + DL), BanglaBERT	**80.11%**

## Conclusions

This study addresses the significant gap in Bangla sentiment analysis by introducing a balanced, three-class sentiment dataset curated from authentic social media content. For development and experimentation, an NLP classification system capable of handling the linguistic complexities of Bangla, a low-resource language that has been largely neglected in the NLP literature, was targeted. Several machine learning and deep learning models for sentiment classification were systematically investigated, together with preprocessing, feature extraction, and hyperparameter tuning. Classical models such as Random Forest and XGBoost perform well after tuning, but deep learning models (GRUs in particular) outperform long short-term memory (LSTM) in capturing sentiment nuances. However, the most interesting findings were obtained via the BanglaBERT model, which achieved the best performance (approximately 80%) and the best generalizability and class distinction. This demonstrates the promise of transformer-based models for adapting monolingual, domain-specific data. Importantly, a scalable approach and benchmark dataset are presented to support future sentiment analysis research in Bangla and other low-resource languages. However, some issues still remain. Although the ensemble model has been tuned and set up carefully, a certain degree of misclassification is inevitable (in particular, those between negatives and neutrals) because feature representations and class separations may be further elaborated. Future directions can consider extending this framework to multimodal sentiment signals (images and audio), more topics and dialects with added diversity in dataset collection and transfer learning approaches between linguistically close languages. The extension to multimodal emotion analysis is particularly promising and can serve as a future perspective to enhance the novelty and applicability of Bangla sentiment research. Additionally, cross-domain validation on external Bangla datasets is included as future work, along with the incorporation of Romanized Bangla and Bangla–English code-mixed content to enhance the robustness and real-world applicability of the proposed models.
