# Supplementary material for: Sentiment analysis in Bangla: A three-class dataset and comparative evaluation of machine learning, deep learning, and transformer approaches
Source: PLoS One. 2026 Oct 27;21(10):e0360952. doi: 10.1371/journal.pone.0360952 (PMC13712687; doi:10.1371/journal.pone.0360952)
Supplement: Supplementary file 1 [file pone.0360952.s002.docx]

# **Authors' Response to Editor and Reviewers**

**Title:** “Sentiment Analysis in Bangla: A Three-Class Dataset and Comparative Evaluation of Machine Learning, Deep Learning, and Transformer Approaches”

Submission ID: PONE-D-25-50471

# **Response to Reviewer 1: (Color code = Red)**

### **Point 1:** claim that the main reason of less work of sentiment analysis in Bangla language is due to class imbalance issue. This is not true for sentiment analysis. Sentiment analysis Datasets are always balanced.

**Author Response:** Thank you for your valuable comment. We agree that many sentiment analysis datasets are designed to be balanced, and our previous wording may have created a broader impression than intended. Our intention was not to claim that all sentiment datasets suffer from imbalance. Rather, we were referring to a common situation observed in several existing Bangla resources, where available data are often limited in size, collected from narrow domains, and may show uneven distribution across sentiment classes. Such imbalance can make it more difficult for models to accurately learn and predict the minority classes, which is why we highlighted it as one of the practical challenges in the Bangla context.

**Author Action:** We have revised the sentence to avoid overgeneralization and to reflect the reviewer’s concern. The updated text in the Introduction section has been modified accordingly. (changes highlighted in red)

### **Point 2:**

### another clean mentioned in the Abstract is that sentiment analysis is mainly carried out using boundary classification and the authors have worked on three classes. This is again wrong claim because sentiment analysis is always considered as positive negative and neutral.

**Author Response:** Thank you for your valuable comment. We agree that sentiment analysis is typically carried out using three fixed categories—positive, negative, and neutral. In the earlier draft, the wording “boundary classification” and “free classes” may have caused misunderstanding. Our intention was not to suggest unconventional or undefined classes. To clarify, we have revised the abstract to remove ambiguous terms and now explicitly highlight that our work focuses on multi-domain Bangla sentiment resources, covering diverse real-world usage.

**Author Action:** We have revised the corresponding sentence in the Abstract to avoid ambiguity and clearly indicate that prior studies mostly focused on limited or in-domain corpora. The updated text in the Abstract has been modified accordingly. (changes highlighted in red)

### **Point 3:**

word2vec is a deep feature and it is used with the machine learning is not proper. Conventional features of tfidf , part of speech tagging and and n gram and their variation such as Uni-gram bi-gram and trigram should be used with the machine learning models.

**Author Response:**

We appreciate the reviewer’s comment. We clarify that Word2Vec was used only for deep learning models, while Count Vectorizer has been used for conventional machine learning models. Additionally, traditional features such as TF-IDF, Part-of-Speech tagging, and n-grams (uni-gram, bi-gram, tri-gram) and their variations are now applied consistently across the experiments.

**Author Action:**

All these updates and clarifications have been incorporated into the Results section and marked as revised. All feature encodings for both machine learning (Count Vectorizer and conventional features) and deep learning (Word2Vec) models are now applied and documented.

### **Point 4:** for deep learning models the features of GloVe and fastText should also be used. As it will be also be interesting that the sentence embedding to the also be applied with deep learning models.

**Author Response:**

We thank the reviewer for the suggestion. As GloVe does not provide Bengali embeddings, we have applied FastText embeddings for our deep learning models. Additionally, sentence-level embeddings have also been incorporated.

**Author Action:**

FastText embeddings and sentence-level embeddings have been applied and the updates have been included in both the Results and Methodology sections. All changes have been marked in red.

### **Point 5:** Bert is used for compassion analysis GPT also be applied.

**Author Response:**

We thank the reviewer for the suggestion. While BERT has been applied for compassion analysis, we have also utilized GPT2_Bengali embeddings.

**Author Action:**

Details regarding the use of GPT2_Bengali embeddings, in addition to BERT, have been added to both the Methodology and Results sections. All changes have been marked in red.

### **Point 6:** data labelling seems to be main cancern. Mentioned in table 2 and 3 for the sample comment in table 4 to be labelled as negative (by 4 annotator) or neutral (6 annotators) while it is very positive seems to be very strange.

**Author Response:**

First of all, thank you very much for your valuable comments. We placed significant importance on data annotation in our work. The selection rule based on which we presented the comments in Table 4 is somewhat complicated. The word “oiling” can carry a negative connotation in Bengali in certain contexts. As a result, some annotators considered the comment neutral, while others labeled it as negative. We highlighted this complex situation to demonstrate the challenges involved in the annotation process. The final annotation was determined through a voting process, where the majority opinion was given priority.

### **Point 7:** table 9. There is no need to put a percentage with each value instead only the percentage symbol can be mentioned in the caption or in the column heading once.

**Author Response:** Thank you for the constructive suggestion. We agree that repeating the percentage symbol with each value in Table 9 is unnecessary and affects readability.

**Author Action:** Table 9 has been revised by removing the percentage symbol from individual values. The percentage symbol (%) is now mentioned once in the column heading/caption to improve clarity and presentation.

### **Point 8:**

table 10. The comparison of results is not proper. Because the purpose model shows 80% result while the existing studies have more than 92% results. Sweet is expected that the data set should be same for results comparison the prepared data set should be used for application of all diverts models in the existing studies.

**Author Response:** Thank you for the insightful comment. We agree that a direct quantitative comparison of performance across different studies can be misleading when the datasets, domains, and experimental settings are not the same. The higher accuracy reported in some existing works is primarily due to domain-specific datasets and controlled experimental conditions. Our intention was not to claim superiority in terms of accuracy, but to position our work within the broader landscape of Bangla sentiment analysis research.

**Author Action:** We have revised the subsection text and the title of Table 10 to clearly state that the results represent a contextual and qualitative overview rather than a direct performance comparison. An explicit clarification has been added explaining that all studies used different datasets, and that applying all existing models to a single unified dataset is beyond the scope of this work. (changes highlighted in red)

### **Point 9:**

### table of comparison of related work in Literature review section also show old references. It is recommended that for improvement in the Manuscript, the authors should learn and cite their research contributions from recent relevant publications from 2024-25 in esteemed journals..few suggestions are as follows:

### (2024). Bdsentillm: A novel llm approach to sentiment analysis of product reviews. IEEE Access.

### (2025). MDKAT: Multimodal Decoupling with Knowledge Aggregation and Transfer for Video Emotion Recognition. IEEE Transactions on Circuits and Systems for Video Technology. doi: 10.1109/TCSVT.2025.3571534

### (2024). Learning to compose diversified prompts for image emotion classification. Computational Visual Media, 10(6), 1169-1183. doi:<https://doi.org/10.1007/s41095-023-0389-6>

### (2025). Intervening in Negative Emotion Contagion on Social Networks Using Reinforcement Learning. IEEE Transactions on Computational Social Systems, 1-12. doi: 10.1109/TCSS.2025.3555607

### (2024, June). Performance Analysis of LSTM and Bi-LSTM Model with Different Optimizers in Bangla Sentiment Analysis. In 2024 15th International Conference on Computing Communication and Networking Technologies (ICCCNT) (pp. 1-7). IEEE.

**Author Response:** We sincerely thank the reviewer for this insightful suggestion. In response:

1. The Literature Review section has been updated to include recent relevant studies (2024–25), covering Bangla sentiment analysis, multimodal emotion recognition, image emotion classification, and negative emotion interventions.
2. Key Bangla sentiment analysis works are now highlighted, including:
   - Shahnaz Ipa et al. (2024) – BdSentiLLM for multilingual product reviews (F1 = 0.89).
   - Khan et al. (2024) – LSTM and Bi-LSTM for Bangla sentiment analysis (accuracy = 99%, F1 = 98%).
   - Bhowmik et al. (2025) – BN-GRAPH combining data augmentation, transformers, and GNNs (accuracy = 89.57%, F1 = 0.8635).
3. Additional relevant studies on multimodal emotion recognition and social network emotion interventions were incorporated, e.g., MDKAT (Jian Wang, 2025), NECM-RLDB (Deng et al., 2025), and prompt-based image emotion classification (Sinuo Deng et al., 2024).
4. A comprehensive comparison table has been prepared for Bangla sentiment analysis, showing dataset sizes, methods, and accuracy/F1-score.

**Author Action:** We have revised the Literature Review to include recent and relevant works from 2024–25. Additionally, a comparison table for Bangla sentiment analysis has been added, integrating both new and older references to provide a comprehensive overview. (changes highlighted in red)

### **Point 10:** It will be interesting to add emotion analysis as well as multimodal analysis. This will bring more novelty or at least be discussed in related work and may be considered and mentioned as future perspective in conclusion section.

**Author Response:** We sincerely thank the reviewer for this valuable suggestion. We acknowledge that incorporating emotion analysis and multimodal approaches can enhance the scope and novelty of the study. Accordingly, we have discussed relevant works on multimodal emotion recognition, image emotion classification, and social network emotion interventions in the updated Literature Review section to facilitate current research trends. In addition, we have highlighted the potential extension to multimodal emotion analysis as a future perspective in the Conclusion section.

**Author Action:** We have revised the Literature Review to incorporate multimodal sentiment and emotion analysis works and updated the Conclusion to emphasize future directions for extending Bangla sentiment research to multimodal data, following the reviewer’s suggestion. (changes highlighted in red)

# **Response to Reviewer 2: (Color code = Green)**

### **Point 1:**

Could you please add some papers published in this year so 2025? I have found no one reference in 2025. Therefore, the manuscript includes limited references from 2025. Please update the literature review with more recent and relevant studies to ensure the paper reflects the current state of research.

**Author Response:** We sincerely thank the reviewer for the suggestion. We have updated the Literature Review to include recent and relevant studies from 2025 and 2024, while retaining older references for context. These additions cover multilingual sentiment analysis, Bangla sentiment modeling, multimodal emotion recognition, prompt-based image emotion classification, and reinforcement learning-based negative emotion intervention, providing a comprehensive overview of current research trends.

**Author Action:** The Literature Review has been revised to incorporate seven recent publications:

**2025 References:**

- Jian Wang (2025) — MDKAT for multimodal emotion recognition
- Deng et al. (2025) — NECM-RLDB for negative emotion intervention on social networks
- Bhowmik et al. (2025) — BN-GRAPH, Bangla sentiment analysis with data augmentation and graph neural networks
- Ullah (2025) — BCLSA, a concept-level sentiment analysis framework for Bangla

**2024 References:**

- Shahnaz Ipa et al. (2024) — BdSentiLLM for multilingual product review sentiment analysis
- Khan et al. (2024) — LSTM and Bi-LSTM for Bangla sentiment analysis
- Sinuo Deng et al. (2024) — Learning to compose diversified prompts for image emotion classification

### **Point 2:**

Paper organization is missing. Could you please add it to the paper? This would make it more clear to the reader.

**Author Response:** We sincerely thank the reviewer for this suggestion. We have added a “Paper Organization” paragraph at the end of the Introduction section to clearly outline the structure of the manuscript, making it easier for the reader to follow.

**Author Action:** Added a paragraph in the Introduction section describing the paper organization. (changes highlighted in green)

### **Point 3:**

The dataset is collected mainly from Facebook, BBC Bangla, Prothom Alo, YouTube, and a few news-related platforms, so the results may not generalize well to other domains such as product reviews, healthcare, or education. Could you please justify this issue?

**Author Response:** We sincerely thank the reviewer for raising this important point. Although the dataset was primarily collected from platforms such as Facebook, BBC Bangla, Prothom Alo, YouTube, and other news-related sources, the collected content broadly spans multiple domains rather than being confined to a single type. Specifically, in addition to general news and social discussions, we included content related to product reviews, healthcare, education, and various social topics, ensuring diversity in context and language usage. While it is challenging to cover every possible domain, we made a concerted effort to include as many domain-centric contents as feasible, so the dataset provides a representative and comprehensive coverage for Bangla sentiment analysis.

**Author Action:**

### **Point 4:**

The authors exclude Romanized Bangla and many English or mixed-language comments, focusing only on Bangla in Bangla script, which limits the applicability of the models to real-world Bangla–English code-mixed environments. Could you please justify this issue?

**Author Response:** We sincerely thank the reviewer for this valuable observation. In the current study, we focused on Bangla in Bangla script to ensure consistency and robust evaluation on a monolingual dataset. We acknowledge that Romanized Bangla and code-mixed Bangla–English text are prevalent in real-world social media, and their inclusion would enhance model applicability. As part of future work, we plan to extend our framework to handle Romanized and code-mixed content, thereby improving its relevance across multilingual and real-world environments.

**Author Action:** We have updated the Conclusion section to explicitly include potential future work. (changes highlighted in green)

### **Point 5:**

The dataset is made balanced using SMOTE, which creates synthetic samples; this may introduce artificial patterns and may not fully reflect natural sentiment distributions in real social media. Could you please justify this issue to make it further clear to the reader?

**Author Response:** We thank the reviewer for raising this important concern. Class imbalance is a common challenge in sentiment analysis, particularly for low-resource languages such as Bangla. SMOTE has been extensively adopted in prior sentiment analysis research to mitigate bias toward majority classes and to enhance model learning. Following established practice in the literature, we employed SMOTE as a preprocessing strategy to improve class balance while maintaining the integrity of the original dataset. Relevant studies using SMOTE for sentiment analysis have been cited to justify this methodological choice.

1. Obiedat R, Qaddoura R, Ala’M AZ, Al-Qaisi L, Harfoushi O, Alrefai M, et al. Sentiment analysis of customers’ reviews using a hybrid evolutionary SVM-based approach in an imbalanced data distribution. Ieee Access. 2022;10:22260-73.
2. Flores AC, Icoy RI, Peña CF, Gorro KD. An evaluation of svm and naive bayes with smote on sentiment analysis data set. In: 2018 International conference on engineering, applied sciences, and technology (ICEAST). IEEE; 2018. p. 1-4
3. Mujahid M, Lee E, Rustam F, Washington PB, Ullah S, Reshi AA, et al. Sentiment analysis and topic modeling on tweets about online education during COVID-19. Applied Sciences. 2021;11(18):8438.

### **Point 6:**

The models are evaluated only on accuracy (and on one dataset split), with no reported external test set or cross-domain validation, so the robustness and generalization of BanglaBERT and the ensemble across other Bangla datasets remain uncertain.

**Author Response:**

We thank the reviewer for the valuable comment. In our study, the dataset was divided into training and test sets, where the test set was completely unseen during training. The reported accuracy corresponds exclusively to the performance on this unseen test set.

We acknowledge that evaluation on a single dataset split and reporting only accuracy does not fully demonstrate robustness across different domains. Accordingly, cross-domain validation on external Bangla datasets has been explicitly included as future work, and we plan to extend the evaluation with additional datasets and metrics to further assess generalization.

### **Point 7:**

Main grammar and style issues.

**Author Response:** We thank the reviewer for carefully identifying the grammar, spacing, repetition, and consistency issues throughout the manuscript. All the highlighted concerns have been addressed to improve clarity, readability, and stylistic consistency.

**Author Action:** The manuscript has been thoroughly revised, and all suggested grammatical corrections and stylistic refinements have been implemented.

### **Point 8:**

“Banglais largely unexplored” → “Bangla is largely unexplored”.

**Author Response:** We thank the reviewer for this helpful observation.

**Author Action:** The sentence has been corrected by adding the missing space and verb.

### **Point 9:**

“binary classification orindomain corpora” → add space: “binary classification or in-domain corpora”.

**Author Response:** We thank the reviewer for this helpful observation.

**Author Action:** The spacing and hyphenation have been corrected accordingly.

### **Point 10:**

“such abalanced three-class sentiment dataset” → “such a balanced three-class sentiment dataset”.

**Author Response:** We thank the reviewer for this helpful observation.

**Author Action:** The sentence has been revised to correct the spacing and grammatical structure.

### **Point 11:**

“BanglaBERT outperforms all other pretrained and other architectures” → avoid repetition: “BanglaBERT outperforms all other pretrained architectures”.

**Author Response:** We appreciate the reviewer’s careful review and suggestion.

**Author Action:** The sentence has been revised to: “BanglaBERT outperforms all other pretrained architectures.”

### **Point 12:**

Check consistency of terms: sometimes “Bangla Language” is capitalized oddly; better to use “Bangla language” consistently.

**Author Response:** We acknowledge the reviewer’s valuable suggestion.

**Author Action:** The term has been standardized to *“Bangla language”* throughout the manuscript for consistency.

### **Point 13:**

Be consistent with hyphenation such as “three-class”, “in-domain”, and “code-mixed”.

**Author Response:** We thank the reviewer for pointing out this issue.

**Author Action:** Hyphenation has been made consistent across the entire manuscript following standard academic writing conventions.

### **Point 14:**

Comma after equation 1 should be placed please.

**Author Response:** We thank the reviewer for pointing out this punctuation issue.

**Author Action:** A comma has been added after Equation (1) in the text following the equation to ensure grammatical correctness.

### **Point 15:**

Dot after equation 2, 3, 4, 5, 6, 7 should be placed please.

**Author Response:** We thank the reviewer for this helpful suggestion regarding punctuation.

**Author Action:** Dot have been added at the end of Equations 2–7 to ensure proper punctuation and consistency throughout the manuscript.

### **Point 16:**

Comma after equation 8 should be placed please.

**Author Response:** We appreciate the reviewer’s comment.

**Author Action:** A comma has been added after Equation 8, ensuring correct punctuation in both the equation display and in-text references.
