# Supplementary material for: Sentiment analysis in Bangla: A three-class dataset and comparative evaluation of machine learning, deep learning, and transformer approaches
Source: PLoS One. 2026 Oct 27;21(10):e0360952. doi: 10.1371/journal.pone.0360952 (PMC13712687; doi:10.1371/journal.pone.0360952)
Supplement: Supplementary file 2 [file pone.0360952.s003.docx]

# **Authors' Response to Editor and Reviewers**

**Title:** “Sentiment Analysis in Bangla: A Three-Class Dataset and Comparative Evaluation of Machine Learning, Deep Learning, and Transformer Approaches”

Submission ID: PONE-D-25-50471

**Response to Reviewer 2: (Color code = Red)**

**Point 1:**
The author stated that *(Finally, we hope that the).* This is not academic writing. Could you please justify this to make it clear to the reader.

**Author Response:**
Thank you very much for your valuable comment. We agree that the phrase *“we hope”* is subjective and not fully aligned with formal academic writing style. Our intention was to indicate the expected contribution and future usefulness of the released dataset. To improve the academic tone and make the statement clearer for the reader, the sentence has been revised using objective and formal language.

**Author Action:**
The sentence in the Abstract has been revised from *“Finally, we hope that the…”* to a more formal academic expression: *“The release of this dataset is expected to facilitate further advances in Bangla sentiment analysis and provide a scalable foundation for multilingual and cross-lingual NLP research.”*
The revised text has been highlighted in red.

**Point 2:**
I have discovered some punctuation errors. Could you please proofread the paper to make sure that there is no grammar mistake.

**Author Response:**
Thank you for your constructive comment. We appreciate the reviewer’s careful observation. A thorough proofreading of the entire manuscript has been conducted to identify and correct punctuation, grammatical, and typographical errors. Several sentences have been revised to improve clarity, academic tone, and grammatical correctness.

**Author Action:**
The full manuscript has been carefully proofread, and punctuation and grammatical issues throughout the paper have been corrected. All revised text has been highlighted in red.

**Point 3:**
The author has used many times *(we, our)*. This is not preferred in academic writing.

**Author Response:**
Thank you for this important comment. We fully agree that excessive use of first-person pronouns such as *“we”* and *“our”* is not preferred in formal academic writing. To address this concern, the manuscript has been thoroughly revised to replace first-person expressions with objective and impersonal academic phrasing.

**Author Action:**
A full manuscript-wide revision has been performed using search-based correction (CTRL+F), and expressions such as *“we”*, *“our”*, and similar first-person forms have been replaced with formal academic alternatives such as *“this study”*, *“the proposed system”*, *“the dataset”*, and passive constructions.
All updated sections have been highlighted in red.

**Point 4:**
Commas after the symbol in equation 1 not after (1), this is not correct, the correct should , (1)

**Author Response:**
Thank you for pointing out this formatting issue. We agree that the punctuation placement around Equation (1) was not correctly formatted in the earlier version of the manuscript.

**Author Action:**
The equation reference formatting has been corrected throughout the manuscript. Specifically, the incorrect form *“Equation, 1”* has been revised to *“Equation (1)”*, and punctuation placement around equations has been updated according to academic formatting standards. Similar corrections have also been applied consistently to other equations in the manuscript.
All changes have been highlighted in red.

**Point 5:**
The evaluation focuses primarily on accuracy (and limited metrics). Accuracy alone may be misleading, especially under class imbalance. I want you to just give an expectation what would be the results if you considered precision, recall, F1-score. Is everything will be fine just in one sentence if possible.

**Author Response:**
Thank you very much for this valuable suggestion. We fully agree that accuracy alone may be insufficient for evaluating classification performance, especially in the presence of class imbalance. To address this concern, additional evaluation metrics including precision, recall, and F1-score have been explicitly emphasized in the Results and Discussion section.

**Author Action:**
A clarifying sentence has been added in the Results section to indicate that the comparable precision, recall, and F1-score values confirm balanced performance across all sentiment classes and support the reliability of the proposed models under class imbalance conditions.
The newly added sentence is highlighted in red.
