# Supplementary material for: Sentiment analysis in Bangla: A three-class dataset and comparative evaluation of machine learning, deep learning, and transformer approaches
Source: PLoS One. 2026 Oct 27;21(10):e0360952. doi: 10.1371/journal.pone.0360952 (PMC13712687; doi:10.1371/journal.pone.0360952)
Supplement: Supplementary file 3 [file pone.0360952.s004.docx]

**Authors' Response to Editor and Reviewers**

**Title:** “Sentiment Analysis in Bangla: A Three-Class Dataset and Comparative

Evaluation of Machine Learning, Deep Learning, and Transformer Approaches”

Submission ID: PONE-D-25-50471

**Response to Reviewer 3: (Color code = Red)**

**Point 1:**

The class counts in the Data Balancing subsection (lines 393-394: negative 4,139; neutral ~2,826; positive 2,189) do not match those in Table 6 (negative 4,142; positive 2,829; neutral 2,192) and the neutral/positive numbers appear to be reversed. Make these numbers consistent with Table 6.

**Author Response:**

Thank you for identifying this inconsistency. We have corrected the class counts in the Data Balancing subsection and ensured that the class labels and corresponding values are consistent with those reported in Table 6.

**Author Action:**

The Table 6 has been revised to: negative = 4,139, positive = 2,189, and neutral = 2,826. The neutral and positive class values have also been placed under the correct labels.

The revised text has been highlighted in red.

**Point 2:**

Line 371 says that the expanded short-form expressions are "shown in Figure 3", but Figure 3 is the Zipf's Law word-frequency plot. Please fix this cross-reference.

**Author Response:**

Thank you for identifying this incorrect cross-reference. We have revised the statement to remove the erroneous reference to Figure 3 and accurately describe the expansion of short-form expressions in Figure 4 during the text-preprocessing stage.

**Author Action:**

The incorrect cross-reference in line 371 has been updated as Figure 4. The revised text now states that the short-form expressions were expanded during data preprocessing, while Figure 3 remains correctly identified as the Zipf’s Law word-frequency plot.

**Point 3:**

Some sentences lost their subjects in the revision to impersonal style, e.g. line 371 ("In this study, have expanded...") and line 772 ("In this study, introduced several advanced technologies..."). Please put these back into grammatical shape.

**Author Response:**

Thank you for highlighting these grammatical errors. We have restored the missing subjects in the identified sentences and reviewed the revised manuscript for similar issues to ensure grammatical clarity and consistency.

**Author Action:**

The sentences have been corrected as follows:

Line 371: “In this study, we have expanded the short-form expressions...”

Line 772: “In this study, we introduced several advanced technologies...”

Similar subject-related grammatical errors identified elsewhere in the manuscript were also corrected.

**Point 4:**

In the caption of Table 15, it is "GPT2_BERT" whereas in the text and table rows, it is "GPT2_Bengali". Standardise nomenclature.

**Author Response:**

We appreciate the reviewer for drawing our attention to this discrepancy. We have standardized the model nomenclature throughout the manuscript to ensure that the same term is used consistently.

**Author Action:**

The term “GPT2_BENGALI” in the caption of Table 15 has been replaced with “GPT2_Bengali,” consistent with the terminology used in the main text and table rows. The manuscript was also checked for other inconsistent references to this model.

**Point 5:**

Line 878 says "Class 2 (Positive)" but Table 5 shows the label scheme as 0 = Negative, 1 = Positive, 2 = Neutral. Please correct the class label in the GPT2-Bengali discussion.

**Author Response:**

We appreciate the reviewer’s careful observation regarding the class-label assignment. According to the classification scheme presented in Table 5, Class 2 corresponds to the Neutral sentiment. The incorrect label has therefore been corrected.

**Author Action:**

The typographical error has been corrected. In the GPT2-Bengali discussion (Line 878), "Class 2 (Positive)" has been revised to "Class Positive" to maintain consistency with the class-label scheme presented in Table 5.
