# Supplementary material for: Sentiment analysis in Bangla: A three-class dataset and comparative evaluation of machine learning, deep learning, and transformer approaches
Source: PLoS One. 2026 Oct 27;21(10):e0360952. doi: 10.1371/journal.pone.0360952 (PMC13712687; doi:10.1371/journal.pone.0360952)
Supplement: Supplementary file 4 [file pone.0360952.s005.docx]

# **Authors' Response to Editor and Reviewers**

**Title:** “Sentiment Analysis in Bangla: A Three-Class Dataset and Comparative Evaluation of Machine Learning, Deep Learning, and Transformer Approaches”

Submission ID: PONE-D-25-50471R3

**Response to Reviewer 3: (Color code = Red)**

**Point 1: (The Reviewer 3 and Editor provided the same comment)**

Four out of five previous comments have been adequately addressed (the Figure 4 cross-reference, restored sentence subjects at lines 371 and 772, standardized GPT2_BENGALI nomenclature, and the corrected class label in the GPT2 discussion).

Comment 1 is still open. Table 6 values have been changed to Negative 4139, Positive 2189, Neutral 2826, Total 9154, but values in the rest of the manuscript have not been changed accordingly: line 263 and lines 333–336 still have a total of 9163, lines 335–336 still have 2829 and 2192 for the positive and neutral classes (which also still appear in the reversed order), Table 16 has the size of the dataset for the proposed system as 9163, and line 897 has 9,163. The result is that the paragraph introducing Table 6 no longer corresponds to the contents of Table 6. The authors should check the class counts against the deposited Mendeley dataset (doi: 10.17632/rh67mckhbh.2) and propagate the confirmed values consistently to the Data Description, the paragraph before Table 6, Table 16, and the Discussion.

**Author Response:**

We sincerely thank the Reviewer and Editor. We carefully rechecked the dataset statistics and clarified the distinction between the initially collected data and the final processed dataset. Initially, 9,162 comments were collected, including 8 unlabeled comments. During preprocessing, these 8 unlabeled comments were removed, resulting in a final labeled dataset of 9,154 comments. The final dataset consists of 4,139 Negative, 2,189 Positive, and 2,826 Neutral comments. We have ensured that these final dataset counts are now reported consistently throughout the manuscript.

**Author Action:**

We have revised the Data Description, the paragraph preceding Table 6, Table 16, and the relevant statements in the Discussion section. The paragraph introducing Table 6 has also been updated to clearly distinguish the initially collected 9,162 comments, including 8 unlabeled comments, from the final processed dataset of 9,154 labeled comments. Table 6 now consistently reports Negative = 4,139, Positive = 2,189, Neutral = 2,826, and Total = 9,154. The corresponding dataset size and class counts have also been corrected consistently throughout the manuscript.

The revised text has been highlighted in red.

On behalf of the all Authors,

**Jahanur Biswas**

Corresponding Author

jahanurbiswas.jucse@gmail.com
