# Supplementary material for: Sentiment analysis in Bangla: A three-class dataset and comparative evaluation of machine learning, deep learning, and transformer approaches
Source: PLoS One. 2026 Oct 27;21(10):e0360952. doi: 10.1371/journal.pone.0360952 (PMC13712687; doi:10.1371/journal.pone.0360952)
Supplement: Peer review history

# Peer review: Decision Letter 0

**DOI:** 10.1371/journal.pone.0360952.r001

**Authors:** Sadiq Abdulhussain

30 Nov 2025

Dear Dr. Biswas,

Thank you for submitting your manuscript to PLOS ONE. After careful consideration, we feel that it has merit but does not fully meet PLOS ONE’s publication criteria as it currently stands. Therefore, we invite you to submit a revised version of the manuscript that addresses the points raised during the review process.

Please submit your revised manuscript by Jan 14 2026 11:59PM. If you will need more time than this to complete your revisions, please reply to this message or contact the journal office at plosone@plos.org. When you're ready to submit your revision, log on to https://www.editorialmanager.com/pone/ and select the 'Submissions Needing Revision' folder to locate your manuscript file.

- A rebuttal letter that responds to each point raised by the academic editor and reviewer(s). You should upload this letter as a separate file labeled 'Response to Reviewers'.
- A marked-up copy of your manuscript that highlights changes made to the original version. You should upload this as a separate file labeled 'Revised Manuscript with Track Changes'.
- An unmarked version of your revised paper without tracked changes. You should upload this as a separate file labeled 'Manuscript'.

If you would like to make changes to your financial disclosure, please include your updated statement in your cover letter. Guidelines for resubmitting your figure files are available below the reviewer comments at the end of this letter.

If applicable, we recommend that you deposit your laboratory protocols in protocols.io to enhance the reproducibility of your results. Protocols.io assigns your protocol its own identifier (DOI) so that it can be cited independently in the future. For instructions see: https://journals.plos.org/plosone/s/submission-guidelines#loc-laboratory-protocols. Additionally, PLOS ONE offers an option for publishing peer-reviewed Lab Protocol articles, which describe protocols hosted on protocols.io. Read more information on sharing protocols at https://plos.org/protocols?utm_medium=editorial-email&utm_source=authorletters&utm_campaign=protocols.

We look forward to receiving your revised manuscript.

Kind regards,

Sadiq H. Abdulhussain, Ph.D.

Academic Editor

PLOS ONE

Journal Requirements:

When submitting your revision, we need you to address these additional requirements.

1. Please ensure that your manuscript meets PLOS ONE's style requirements, including those for file naming. The PLOS ONE style templates can be found at

https://journals.plos.org/plosone/s/file?id=wjVg/PLOSOne_formatting_sample_main_body.pdf and

https://journals.plos.org/plosone/s/file?id=ba62/PLOSOne_formatting_sample_title_authors_affiliations.pdf

2. In your Methods section, please include additional information about your dataset and ensure that you have included a statement specifying whether the collection and analysis method complied with the terms and conditions for the source of the data.

3. Please provide additional details regarding participant consent. In the ethics statement in the Methods and online submission information, please ensure that you have specified (1) whether consent was informed and (2) what type you obtained (for instance, written or verbal, and if verbal, how it was documented and witnessed). If your study included minors, state whether you obtained consent from parents or guardians. If the need for consent was waived by the ethics committee, please include this information.

If you are reporting a retrospective study of medical records or archived samples, please ensure that you have discussed whether all data were fully anonymized before you accessed them and/or whether the IRB or ethics committee waived the requirement for informed consent. If patients provided informed written consent to have data from their medical records used in research, please include this information.

4. Please note that PLOS One has specific guidelines on code sharing for submissions in which author-generated code underpins the findings in the manuscript. In these cases, we expect all author-generated code to be made available without restrictions upon publication of the work. Please review our guidelines at https://journals.plos.org/plosone/s/materials-and-software-sharing#loc-sharing-code and ensure that your code is shared in a way that follows best practice and facilitates reproducibility and reuse.

5. We note that your Data Availability Statement is currently as follows: “All relevant data are within the manuscript and its Supporting Information files.”

Please confirm at this time whether or not your submission contains all raw data required to replicate the results of your study. Authors must share the “minimal data set” for their submission. PLOS defines the minimal data set to consist of the data required to replicate all study findings reported in the article, as well as related metadata and methods (https://journals.plos.org/plosone/s/data-availability#loc-minimal-data-set-definition).

For example, authors should submit the following data:

- The values behind the means, standard deviations and other measures reported;

- The values used to build graphs;

- The points extracted from images for analysis.

Authors do not need to submit their entire data set if only a portion of the data was used in the reported study.

If your submission does not contain these data, please either upload them as Supporting Information files or deposit them to a stable, public repository and provide us with the relevant URLs, DOIs, or accession numbers. For a list of recommended repositories, please see https://journals.plos.org/plosone/s/recommended-repositories.

If there are ethical or legal restrictions on sharing a de-identified data set, please explain them in detail (e.g., data contain potentially sensitive information, data are owned by a third-party organization, etc.) and who has imposed them (e.g., an ethics committee). Please also provide contact information for a data access committee, ethics committee, or other institutional body to which data requests may be sent. If data are owned by a third party, please indicate how others may request data access.

6. PLOS requires an ORCID iD for the corresponding author in Editorial Manager on papers submitted after December 6th, 2016. Please ensure that you have an ORCID iD and that it is validated in Editorial Manager. To do this, go to ‘Update my Information’ (in the upper left-hand corner of the main menu), and click on the Fetch/Validate link next to the ORCID field. This will take you to the ORCID site and allow you to create a new iD or authenticate a pre-existing iD in Editorial Manager.

7. If the reviewer comments include a recommendation to cite specific previously published works, please review and evaluate these publications to determine whether they are relevant and should be cited. There is no requirement to cite these works unless the editor has indicated otherwise.

[Note: HTML markup is below. Please do not edit.]

Reviewers' comments:

Reviewer's Responses to Questions

**Comments to the Author**

1. Is the manuscript technically sound, and do the data support the conclusions?

Reviewer #1: Yes

Reviewer #2: Yes

**********

2. Has the statistical analysis been performed appropriately and rigorously?

Reviewer #1: Yes

Reviewer #2: Yes

**********

3. Have the authors made all data underlying the findings in their manuscript fully available?

The PLOS Data policy

Reviewer #1: Yes

Reviewer #2: No

**********

4. Is the manuscript presented in an intelligible fashion and written in standard English?

Reviewer #1: Yes

Reviewer #2: Yes

**********

Reviewer #1: In this man script, the authors have worked on sentiment analysis for Bangla content. Proposed the deep learning based Transformer model for the implementation.

Following are the main concern:

1) claim that the main reason of less work of sentiment analysis in Bangla language is due to class imbalance issue. This is not true for sentiment analysis. Sentiment analysis Datasets are always balanced.

2) another clean mentioned in the Abstract is that sentiment analysis is mainly carried out using boundary classification and the authors have worked on free classes. This is again wrong claim because sentiment analysis is always considered as positive negative and neutral.

3) word2vec is a deep feature and it is used with the machine learning is not proper. Conventional features of tfidf , part of speech tagging and and n gram and their variation such as Uni-gram bi-gram and trigram should be used with the machine learning models.

4) for deep learning models the features of GloVe and fastTextshould also be used. As it will be also be interesting that the sentence I'm waiting to the also be applied with deep learning models.

5) Bert is used for compassion analysis GPT also be applied

6) data labelling seems to be main cancern . Mentioned in table 2 and 3 for the sample comment in table 4 to be labelled as negative (by 4 annotator ) or neutral (6 annotators) while it is very positive seems to be very strange.

7) table 9. There is no need to put a percentage with each value instead only the percentage symbol can be mentioned in the cap tion or in the colum heading once.

8) table 10. The comparison of results is not proper. Because the purpose model shows 80% result while the existing studies have more than 92% results. Sweet is expected that the data set should be same for results comparison the prepared data set should be used for application of all diverts models in the existing studies.

9) table of comparison of related work in Literature review section also show old references. It is recommended that for improvement in the Manuscript, the authors should learn and cite their research contributions from recent relevant publications from 2024-25 in esteemed journals..few suggestions are as follows:

(2024). Bdsentillm: A novel llm approach to sentiment analysis of product reviews. IEEE Access.

(2025). MDKAT: Multimodal Decoupling with Knowledge Aggregation and Transfer for Video Emotion Recognition. IEEE Transactions on Circuits and Systems for Video Technology. doi: 10.1109/TCSVT.2025.3571534

(2024). Learning to compose diversified prompts for image emotion classification. Computational Visual Media, 10(6), 1169-1183. doi: https://doi.org/10.1007/s41095-023-0389-6

(2025). Intervening in Negative Emotion Contagion on Social Networks Using Reinforcement Learning. IEEE Transactions on Computational Social Systems, 1-12. doi: 10.1109/TCSS.2025.3555607

(2024, June). Performance Analysis of LSTM and Bi-LSTM Model with Different Optimizers in Bangla Sentiment Analysis. In 2024 15th International Conference on Computing Communication and Networking Technologies (ICCCNT) (pp. 1-7). IEEE.

10) It will be interesting to add emotion analysis as well as multimodal analysis. This will bring more novelty or at least be discussed in related work and may be considered and mentioned as future perspective in conclusion section.

Reviewer #2: This paper addresses the lack of high-quality sentiment analysis resources for the Bangla language. To overcome this problem, the authors created a new balanced three-class sentiment dataset (positive, negative, neutral) built from real Bangla social media posts, each annotated by native speakers. They then applied a comprehensive preprocessing pipeline to clean the text, including denoising, expanding abbreviations, and tokenization. To evaluate the dataset, the authors extracted features using Count Vectorizer and Word2Vec, and trained multiple machine learning models (SVM, Random Forest, XGBoost) as well as deep learning models (GRU, LSTM). They also fine-tuned the BanglaBERT transformer model to compare its performance with the traditional approaches. The experiments showed that BanglaBERT achieved the highest accuracy (80%), outperforming all other models. This demonstrates the strong capability of transformer-based models even for low-resource languages like Bangla. Finally, the authors state that the release of their dataset will support future research in Bangla sentiment analysis and help advance multilingual and cross-lingual NLP studies.

The paper is well organized but not very well written. However, I have discovered some minor issues that need to be justified and addressed.

1. Could you please add some papers purplish in this year so 2025? I have found no one reference in 2025. Therefore, the manuscript includes limited references from 2025. Please update the literature review with more recent and relevant studies to ensure the paper reflects the current state of research.

2. Paper organization is missing could you please added to the paper. This would make it more clear to the reader.

3. The dataset is collected mainly from Facebook, BBC Bangla, Prothom Alo, YouTube, and a few news-related platforms, so the results may not generalize well to other domains such as product reviews, healthcare, or education. Could you please justify this issue please.

4. The authors exclude Romanized Bangla and many English or mixed-language comments, focusing only on Bangla in Bangla script, which limits the applicability of the models to real-world Bangla–English code-mixed environments. Could you please justify this issue please.

5. The dataset is made balanced using SMOTE, which creates synthetic samples; this may introduce artificial patterns and may not fully reflect natural sentiment distributions in real social media. Could you please justify this issue to make it further clear to the reader.

6. The models are evaluated only on accuracy (and on one dataset split), with no reported external test set or cross-domain validation, so the robustness and generalization of BanglaBERT and the ensemble across other Bangla datasets remain uncertain.

7. Main grammar and style issues

8. “Banglais largely unexplored” → “Bangla is largely unexplored”.

9. “binary classification orindomain corpora” → add space: “binary classification or in-domain corpora”.

10. “such abalanced three-class sentiment dataset” → “such a balanced three-class sentiment dataset”.

11. “BanglaBERT outperforms all other pretrained and other architectures” → avoid repetition: “BanglaBERT outperforms all other pretrained architectures”.

12. Check consistency of terms: sometimes “Bangla Language” is capitalized oddly; better to use “Bangla language” consistently.

13. Be consistent with hyphenation such as “three-class”, “in-domain”, and “code-mixed”.

14. Comma after equation 1 should be placed please.

15. Dot after equation 2 and 3, 4,5,6 7 should be placed please

16. Comma after equation 8 should be placed please

**********

what does this mean?). If published, this will include your full peer review and any attached files.

If you choose “no”, your identity will remain anonymous but your review may still be made public.

**Do you want your identity to be public for this peer review?**  For information about this choice, including consent withdrawal, please see our Privacy Policy

Reviewer #1: No

Reviewer #2: No

**********

[NOTE: If reviewer comments were submitted as an attachment file, they will be attached to this email and accessible via the submission site. Please log into your account, locate the manuscript record, and check for the action link "View Attachments". If this link does not appear, there are no attachment files.]

To ensure your figures meet our technical requirements, please review our figure guidelines: https://journals.plos.org/plosone/s/figures

You may also use PLOS’s free figure tool, NAAS, to help you prepare publication quality figures: https://journals.plos.org/plosone/s/figures#loc-tools-for-figure-preparation.

NAAS will assess whether your figures meet our technical requirements by comparing each figure against our figure specifications.

# Author comment: Author response to Decision Letter 1

**DOI:** 10.1371/journal.pone.0360952.r002

6 Jan 2026

Authors' Response to Editor and Reviewers

Title: “Sentiment Analysis in Bangla: A Three-Class Dataset and Comparative Evaluation of Machine Learning, Deep Learning, and Transformer Approaches”

Submission ID: PONE-D-25-50471

Response to Reviewer 1: (Color code = Red)

Point 1:

claim that the main reason of less work of sentiment analysis in Bangla language is due to class imbalance issue. This is not true for sentiment analysis. Sentiment analysis Datasets are always balanced.

Author Response: Thank you for your valuable comment. We agree that many sentiment analysis datasets are designed to be balanced, and our previous wording may have created a broader impression than intended. Our intention was not to claim that all sentiment datasets suffer from imbalance. Rather, we were referring to a common situation observed in several existing Bangla resources, where available data are often limited in size, collected from narrow domains, and may show uneven distribution across sentiment classes. Such imbalance can make it more difficult for models to accurately learn and predict the minority classes, which is why we highlighted it as one of the practical challenges in the Bangla context.

Author Action: We have revised the sentence to avoid overgeneralization and to reflect the reviewer’s concern. The updated text in the Introduction section has been modified accordingly. (changes highlighted in red)

________________________________________

Point 2:

another clean mentioned in the Abstract is that sentiment analysis is mainly carried out using boundary classification and the authors have worked on three classes. This is again wrong claim because sentiment analysis is always considered as positive negative and neutral.

Author Response: Thank you for your valuable comment. We agree that sentiment analysis is typically carried out using three fixed categories—positive, negative, and neutral. In the earlier draft, the wording “boundary classification” and “free classes” may have caused misunderstanding. Our intention was not to suggest unconventional or undefined classes. To clarify, we have revised the abstract to remove ambiguous terms and now explicitly highlight that our work focuses on multi-domain Bangla sentiment resources, covering diverse real-world usage.

Author Action: We have revised the corresponding sentence in the Abstract to avoid ambiguity and clearly indicate that prior studies mostly focused on limited or in-domain corpora. The updated text in the Abstract has been modified accordingly. (changes highlighted in red)

________________________________________

Point 3:

word2vec is a deep feature and it is used with the machine learning is not proper. Conventional features of tfidf , part of speech tagging and and n gram and their variation such as Uni-gram bi-gram and trigram should be used with the machine learning models.

Author Response:

We appreciate the reviewer’s comment. We clarify that Word2Vec was used only for deep learning models, while Count Vectorizer has been used for conventional machine learning models. Additionally, traditional features such as TF-IDF, Part-of-Speech tagging, and n-grams (uni-gram, bi-gram, tri-gram) and their variations are now applied consistently across the experiments.

Author Action:

All these updates and clarifications have been incorporated into the Results section and marked as revised. All feature encodings for both machine learning (Count Vectorizer and conventional features) and deep learning (Word2Vec) models are now applied and documented.

________________________________________

Point 4:

for deep learning models the features of GloVe and fastText should also be used. As it will be also be interesting that the sentence embedding to the also be applied with deep learning models.

Author Response:

We thank the reviewer for the suggestion. As GloVe does not provide Bengali embeddings, we have applied FastText embeddings for our deep learning models. Additionally, sentence-level embeddings have also been incorporated.

Author Action:

FastText embeddings and sentence-level embeddings have been applied and the updates have been included in both the Results and Methodology sections. All changes have been marked in red.

________________________________________

Point 5:

Bert is used for compassion analysis GPT also be applied.

Author Response:

We thank the reviewer for the suggestion. While BERT has been applied for compassion analysis, we have also utilized GPT2_Bengali embeddings.

Author Action:

Details regarding the use of GPT2_Bengali embeddings, in addition to BERT, have been added to both the Methodology and Results sections. All changes have been marked in red.

________________________________________

Point 6:

data labelling seems to be main cancern. Mentioned in table 2 and 3 for the sample comment in table 4 to be labelled as negative (by 4 annotator) or neutral (6 annotators) while it is very positive seems to be very strange.

Author Response:

First of all, thank you very much for your valuable comments. We placed significant importance on data annotation in our work. The selection rule based on which we presented the comments in Table 4 is somewhat complicated. The word “oiling” can carry a negative connotation in Bengali in certain contexts. As a result, some annotators considered the comment neutral, while others labeled it as negative. We highlighted this complex situation to demonstrate the challenges involved in the annotation process. The final annotation was determined through a voting process, where the majority opinion was given priority.

________________________________________

Point 7:

table 9. There is no need to put a percentage with each value instead only the percentage symbol can be mentioned in the caption or in the column heading once.

Author Response: Thank you for the constructive suggestion. We agree that repeating the percentage symbol with each value in Table 9 is unnecessary and affects readability.

Author Action: Table 9 has been revised by removing the percentage symbol from individual values. The percentage symbol (%) is now mentioned once in the column heading/caption to improve clarity and presentation.

________________________________________

Point 8:

table 10. The comparison of results is not proper. Because the purpose model shows 80% result while the existing studies have more than 92% results. Sweet is expected that the data set should be same for results comparison the prepared data set should be used for application of all diverts models in the existing studies.

Author Response: Thank you for the insightful comment. We agree that a direct quantitative comparison of performance across different studies can be misleading when the datasets, domains, and experimental settings are not the same. The higher accuracy reported in some existing works is primarily due to domain-specific datasets and controlled experimental conditions. Our intention was not to claim superiority in terms of accuracy, but to position our work within the broader landscape of Bangla sentiment analysis research.

Author Action: We have revised the subsection text and the title of Table 10 to clearly state that the results represent a contextual and qualitative overview rather than a direct performance comparison. An explicit clarification has been added explaining that all studies used different datasets, and that applying all existing models to a single unified dataset is beyond the scope of this work. (changes highlighted in red)

________________________________________

Point 9:

table of comparison of related work in Literature review section also show old references. It is recommended that for improvement in the Manuscript, the authors should learn and cite their research contributions from recent relevant publications from 2024-25 in esteemed journals..few suggestions are as follows:

(2024). Bdsentillm: A novel llm approach to sentiment analysis of product reviews. IEEE Access.

(2025). MDKAT: Multimodal Decoupling with Knowledge Aggregation and Transfer for Video Emotion Recognition. IEEE Transactions on Circuits and Systems for Video Technology. doi: 10.1109/TCSVT.2025.3571534

(2024). Learning to compose diversified prompts for image emotion classification. Computational Visual Media, 10(6), 1169-1183. doi: https://doi.org/10.1007/s41095-023-0389-6

(2025). Intervening in Negative Emotion Contagion on Social Networks Using Reinforcement Learning. IEEE Transactions on Computational Social Systems, 1-12. doi: 10.1109/TCSS.2025.3555607

(2024, June). Performance Analysis of LSTM and Bi-LSTM Model with Different Optimizers in Bangla Sentiment Analysis. In 2024 15th International Conference on Computing Communication and Networking Technologies (ICCCNT) (pp. 1-7). IEEE.

Author Response: We sincerely thank the reviewer for this insightful suggestion. In response:

1. The Literature Review section has been updated to include recent relevant studies (2024–25), covering Bangla sentiment analysis, multimodal emotion recognition, image emotion classification, and negative emotion interventions.

2. Key Bangla sentiment analysis works are now highlighted, including:

○ Shahnaz Ipa et al. (2024) – BdSentiLLM for multilingual product reviews (F1 = 0.89).

○ Khan et al. (2024) – LSTM and Bi-LSTM for Bangla sentiment analysis (accuracy = 99%, F1 = 98%).

○ Bhowmik et al. (2025) – BN-GRAPH combining data augmentation, transformers, and GNNs (accuracy = 89.57%, F1 = 0.8635).

3. Additional relevant studies on multimodal emotion recognition and social network emotion interventions were incorporated, e.g., MDKAT (Jian Wang, 2025), NECM-RLDB (Deng et al., 2025), and prompt-based image emotion classification (Sinuo Deng et al., 2024).

4. A comprehensive comparison table has been prepared for Bangla sentiment analysis, showing dataset sizes, methods, and accuracy/F1-score.

Author Action: We have revised the Literature Review to include recent and relevant works from 2024–25. Additionally, a comparison table for Bangla sentiment analysis has been added, integrating both new and older references to provide a comprehensive overview. (changes highlighted in red)

________________________________________

Point 10:

It will be interesting to add emotion analysis as well as multimodal analysis. This will bring more novelty or at least be discussed in related work and may be considered and mentioned as future perspective in conclusion section.

Author Response: We sincerely thank the reviewer for this valuable suggestion. We acknowledge that incorporating emotion analysis and multimodal approaches can enhance the scope and novelty of the study. Accordingly, we have discussed relevant works on multimodal emotion recognition, image emotion classification, and social network emotion interventions in the updated Literature Review section to facilitate current research trends. In addition, we have highlighted the potential extension to multimodal emotion analysis as a future perspective in the Conclusion section.

Author Action: We have revised the Literature Review to incorporate multimodal sentiment and emotion analysis works and updated the Conclusion to emphasize future directions for extending Bangla sentiment research to multimodal data, following the reviewer’s suggestion. (changes highlighted in red)

________________________________________

Response to Reviewer 2: (Color code = Green)

Point 1:

Could you please add some papers published in this year so 2025? I have found no one reference in 2025. Therefore, the manuscript includes limited references from 2025. Please update the literature review with more recent and relevant studies to ensure the paper reflects the current state of research.

Author Response: We sincerely thank the reviewer for the suggestion. We have updated the Literature Review to include recent and relevant studies from 2025 and 2024, while retaining older references for context. These additions cover multilingual sentiment analysis, Bangla sentiment modeling, multimodal emotion recognition, prompt-based image emotion classification, and reinforcement learning-based negative emotion intervention, providing a comprehensive overview of current research trends.

Author Action: The Literature Review has been revised to incorporate seven recent publications:

2025 References:

● Jian Wang (2025) — MDKAT for multimodal emotion recognition

● Deng et al. (2025) — NECM-RLDB for negative emotion intervention on social networks

● Bhowmik et al. (2025) — BN-GRAPH, Bangla sentiment analysis with data augmentation and graph neural networks

● Ullah (2025) — BCLSA, a concept-level sentiment analysis framework for Bangla

2024 References:

● Shahnaz Ipa et al. (2024) — BdSentiLLM for multilingual product review sentiment analysis

● Khan et al. (2024) — LSTM and Bi-LSTM for Bangla sentiment analysis

● Sinuo Deng et al. (2024) — Learning to compose diversified prompts for image emotion classification

________________________________________

Point 2:

Paper organization is missing. Could you please add it to the paper? This would make it more clear to the reader.

Author Response: We sincerely thank the reviewer for this suggestion. We have added a “Paper Organization” paragraph at the end of the Introduction section to clearly outline the structure of the manuscript, making it easier for the reader to follow.

Author Action: Added a paragraph in the Introduction section describing the paper organization. (changes highlighted in green)

________________________________________

Point 3:

The dataset is collected mainly from Facebook, BBC Bangla, Prothom Alo, YouTube, and a few news-related platforms, so the results may not generalize well to other domains such as product reviews, healthcare, or education. Could you please justify this issue?

Author Response: We sincerely thank the reviewer for raising this important point. Although the dataset was primarily collected from platforms such as Facebook, BBC Bangla, Prothom Alo, YouTube, and other news-related sources, the collected content broadly spans multiple domains rather than being confined to a single type. Specifically, in addition to general news and social discussions, we included content related to product reviews, healthcare, education, and various social topics, ensuring diversity in context and language usage. While it is challenging to cover every possible domain, we made a concerted effort to include as many domain-centric contents as feasible, so the dataset provides a representative and comprehensive coverage for Bangla sentiment analysis.

Author Action:

________________________________________

Point 4:

The authors exclude Romanized Bangla and many English or mixed-language comments, focusing only on Bangla in Bangla script, which limits the applicability of the models to real-world Bangla–English code-mixed environments. Could you please justify this issue?

Author Response: We sincerely thank the reviewer for this valuable observation. In the current study, we focused on Bangla in Bangla script to ensure consistency and robust evaluation on a monolingual dataset. We acknowledge that Romanized Bangla and code-mixed Bangla–English text are prevalent in real-world social media, and their inclusion would enhance model applicability. As part of future work, we plan to extend our framework to handle Romanized and code-mixed content, thereby improving its relevance across multilingual and real-world environments.

Author Action: We have updated the Conclusion section to explicitly include potential future work. (changes highlighted in green)

________________________________________

Point 5:

The dataset is made balanced using SMOTE, which creates synthetic samples; this may introduce artificial patterns and may not fully reflect natural sentiment distributions in real social media. Could you please justify this issue to make it further clear to the reader?

Author Response: We thank the reviewer for raising this important concern. Class imbalance is a common challenge in sentiment analysis, particularly for low-resource languages such as Bangla. SMOTE has been extensively adopted in prior sentiment analysis research to mitigate bias toward majority classes and to enhance model learning. Following established practice in the literature, we employed SMOTE as a preprocessing strategy to improve class balance while maintaining the integrity of the original dataset. Relevant studies using SMOTE for sentiment analysis have been cited to justify this methodological choice.

1. Obiedat R, Qaddoura R, Ala’M AZ, Al-Qaisi L, Harfoushi O, Alrefai M, et al. Sentiment analysis of customers’ reviews using a hybrid evolutionary SVM-based approach in an imbalanced data distribution. Ieee Access. 2022;10:22260-73.

2. Flores AC, Icoy RI, Peña CF, Gorro KD. An evaluation of svm and naive bayes with smote on sentiment analysis data set. In: 2018 International conference on engineering, applied sciences, and technology (ICEAST). IEEE; 2018. p. 1-4

3. Mujahid M, Lee E, Rustam F, Washington PB, Ullah S, Reshi AA, et al. Sentiment analysis and topic modeling on tweets about online education during COVID-19. Applied Sciences. 2021;11(18):8438.

________________________________________

Point 6:

The models are evaluated only on accuracy (and on one dataset split), with no reported external test set or cross-domain validation, so the robustness and generalization of BanglaBERT and the ensemble across other Bangla datasets remain uncertain.

Author Response:

We thank the reviewer for the valuable comment. In our study, the dataset was divided into training and test sets, where the test set was completely unseen during training. The reported accuracy corresponds exclusively to the performance on this unseen test set.

We acknowledge that evaluation on a single dataset split and reporting only accuracy does not fully demonstrate robustness across different domains. Accordingly, cross-domain validation on external Bangla datasets has been explicitly included as future work, and we plan to extend the evaluation with additional datasets and metrics to further assess generalization.

________________________________________

Point 7:

Main grammar and style issues.

Author Response: We thank the reviewer for carefully identifying the grammar, spacing, repetition, and consistency issues throughout the manuscript. All the highlighted concerns have been addressed to improve clarity, readability, and stylistic consistency.

Author Action: The manuscript has been thoroughly revised, and all suggested grammatical corrections and stylistic refinements have been implemented.

________________________________________

Point 8:

“Banglais largely unexplored” → “Bangla is largely unexplored”.

Author Response: We thank the reviewer for this helpful observation.

Author Action: The sentence has been corrected by adding the missing space and verb.

________________________________________

Point 9:

“binary classification orindomain corpora” → add space: “binary classification or in-domain corpora”.

Author Response: We thank the reviewer for this helpful observation.

Author Action: The spacing and hyphenation have been corrected accordingly.

________________________________________

Point 10:

“such abalanced three-class sentiment dataset” → “such a balanced three-class sentiment dataset”.

Author Response: We thank the reviewer for this helpful observation.

Author Action: The sentence has been revised to correct the spacing and grammatical structure.

________________________________________

Point 11:

“BanglaBERT outperforms all other pretrained and other architectures” → avoid repetition: “BanglaBERT outperforms all other pretrained architectures”.

Author Response: We appreciate the reviewer’s careful review and suggestion.

Author Action: The sentence has been revised to: “BanglaBERT outperforms all other pretrained architectures.”

________________________________________

Point 12:

Check consistency of terms: sometimes “Bangla Language” is capitalized oddly; better to use “Bangla language” consistently.

Author Response: We acknowledge the reviewer’s valuable suggestion.

Author Action: The term has been standardized to “Bangla language” throughout the manuscript for consistency.

________________________________________

Point 13:

Be consistent with hyphenation such as “three-class”, “in-domain”, and “code-mixed”.

Author Response: We thank the reviewer for pointing out this issue.

Author Action: Hyphenation has been made consistent across the entire manuscript following standard academic writing conventions.

________________________________________

Point 14:

Comma after equation 1 should be placed please.

Author Response: We thank the reviewer for pointing out this punctuation issue.

Author Action: A comma has been added after Equation (1) in the text following the equation to ensure grammatical correctness.

________________________________________

Point 15:

Dot after equation 2, 3, 4, 5, 6, 7 should be placed please.

Author Response: We thank the reviewer for this helpful suggestion regarding punctuation.

Author Action: Dot have been added at the end of Equations 2–7 to ensure proper punctuation and consistency throughout the manuscript.

________________________________________

Point 16:

Comma after equation 8 should be placed please.

Author Response: We appreciate the reviewer’s comment.

Author Action: A comma has been added after Equation 8, ensuring correct punctuation in both the equation display and in-text references.

________________________________________

Attachment Submitted filename: Author_Response_ Plos One.docx

# Peer review: Decision Letter 1

**DOI:** 10.1371/journal.pone.0360952.r003

**Authors:** Sadiq Abdulhussain

29 Mar 2026

Dear Dr. Biswas,

Thank you for submitting your manuscript to PLOS ONE. After careful consideration, we feel that it has merit but does not fully meet PLOS ONE’s publication criteria as it currently stands. Therefore, we invite you to submit a revised version of the manuscript that addresses the points raised during the review process.

Please submit your revised manuscript by May 13 2026 11:59PM. If you will need more time than this to complete your revisions, please reply to this message or contact the journal office at plosone@plos.org. When you're ready to submit your revision, log on to https://www.editorialmanager.com/pone/ and select the 'Submissions Needing Revision' folder to locate your manuscript file.

- A letter that responds to each point raised by the academic editor and reviewer(s). You should upload this letter as a separate file labeled 'Response to Reviewers'.
- A marked-up copy of your manuscript that highlights changes made to the original version. You should upload this as a separate file labeled 'Revised Manuscript with Track Changes'.
- An unmarked version of your revised paper without tracked changes. You should upload this as a separate file labeled 'Manuscript'.

If you would like to make changes to your financial disclosure, please include your updated statement in your cover letter. Guidelines for resubmitting your figure files are available below the reviewer comments at the end of this letter.

If applicable, we recommend that you deposit your laboratory protocols in protocols.io to enhance the reproducibility of your results. Protocols.io assigns your protocol its own identifier (DOI) so that it can be cited independently in the future. For instructions see: https://journals.plos.org/plosone/s/submission-guidelines#loc-laboratory-protocols. Additionally, PLOS ONE offers an option for publishing peer-reviewed Lab Protocol articles, which describe protocols hosted on protocols.io. Read more information on sharing protocols at https://plos.org/protocols?utm_medium=editorial-email&utm_source=authorletters&utm_campaign=protocols.

As the corresponding author, your ORCID iD is verified in the submission system and will appear in the published article. PLOS supports the use of ORCID, and we encourage all coauthors to register for an ORCID iD and use it as well. Please encourage your coauthors to verify their ORCID iD within the submission system before final acceptance, as unverified ORCID iDs will not appear in the published article. *Only*  the individual author can complete the verification step; PLOS staff *cannot*  verify ORCID iDs on behalf of authors.

We look forward to receiving your revised manuscript.

Kind regards,

Sadiq H. Abdulhussain, Ph.D.

Academic Editor

PLOS One

Journal Requirements:

1. If the reviewer comments include a recommendation to cite specific previously published works, please review and evaluate these publications to determine whether they are relevant and should be cited. There is no requirement to cite these works unless the editor has indicated otherwise.

2. Please review your reference list to ensure that it is complete and correct. If you have cited papers that have been retracted, please include the rationale for doing so in the manuscript text, or remove these references and replace them with relevant current references. Any changes to the reference list should be mentioned in the rebuttal letter that accompanies your revised manuscript. If you need to cite a retracted article, indicate the article’s retracted status in the References list and also include a citation and full reference for the retraction notice.

[Note: HTML markup is below. Please do not edit.]

Reviewers' comments:

Reviewer's Responses to Questions

**Comments to the Author**

Reviewer #1: All comments have been addressed

Reviewer #2: All comments have been addressed

**********

2. Is the manuscript technically sound, and do the data support the conclusions?

Reviewer #1: Yes

Reviewer #2: Yes

**********

3. Has the statistical analysis been performed appropriately and rigorously?

Reviewer #1: Yes

Reviewer #2: Yes

**********

4. Have the authors made all data underlying the findings in their manuscript fully available?

The PLOS Data policy

Reviewer #1: Yes

Reviewer #2: Yes

**********

5. Is the manuscript presented in an intelligible fashion and written in standard English?

Reviewer #1: Yes

Reviewer #2: Yes

**********

Reviewer #1: The authors have addressed all my comments. The paper is in good enough form to be accepted . Good improved work.

Reviewer #2: This paper presents a balanced three-class Bangla sentiment analysis dataset constructed from real-world Bangla social media texts, where posts are manually annotated by native speakers as positive, negative, or neutral. To enhance data quality, the authors implement a preprocessing pipeline including denoising, abbreviation expansion, and tokenization. The paper has been modified. I have the following minor points

The author stated that (Finally, we hope that the). This is not acadmic writing Could you please justify this to make it clear to the reader.

I have discover some punctuation errors could you please proofread the paper to make sure that there is no grammar mistake.

The author has used many time (we, our) this is nor preferred in the academic writing please. Just try to make CTRL+F and write (we) and see how many (we) you used in this paper. THIS IS NOT CORRECT.

Commas after the symbol in equation 1 not after (1), this is not correct, the correct should , (1)

The evaluation focuses primarily on accuracy (and limited metrics). Accuracy alone maybe misleading, especially under class imbalance.

I want you to just give an expection what would be the reustls if you considered precision, recall, F1-score. Is everything will be fine just in one sentence if possible.

**********

what does this mean?). If published, this will include your full peer review and any attached files.

If you choose “no”, your identity will remain anonymous but your review may still be made public.

**Do you want your identity to be public for this peer review?**  For information about this choice, including consent withdrawal, please see our Privacy Policy

Reviewer #1: No

Reviewer #2: No

**********

[NOTE: If reviewer comments were submitted as an attachment file, they will be attached to this email and accessible via the submission site. Please log into your account, locate the manuscript record, and check for the action link "View Attachments". If this link does not appear, there are no attachment files.]

To ensure your figures meet our technical requirements, please review our figure guidelines: https://journals.plos.org/plosone/s/figures

You may also use PLOS’s free figure tool, NAAS, to help you prepare publication quality figures: https://journals.plos.org/plosone/s/figures#loc-tools-for-figure-preparation.

NAAS will assess whether your figures meet our technical requirements by comparing each figure against our figure specifications.

# Author comment: Author response to Decision Letter 2

**DOI:** 10.1371/journal.pone.0360952.r004

2 Apr 2026

Authors' Response to Editor and Reviewers

Title: “Sentiment Analysis in Bangla: A Three-Class Dataset and Comparative Evaluation of Machine Learning, Deep Learning, and Transformer Approaches”

Submission ID: PONE-D-25-50471

Response to Reviewer 2: (Color code = Red)

Point 1:

The author stated that (Finally, we hope that the). This is not academic writing. Could you please justify this to make it clear to the reader.

Author Response:

Thank you very much for your valuable comment. We agree that the phrase “we hope” is subjective and not fully aligned with formal academic writing style. Our intention was to indicate the expected contribution and future usefulness of the released dataset. To improve the academic tone and make the statement clearer for the reader, the sentence has been revised using objective and formal language.

Author Action:

The sentence in the Abstract has been revised from “Finally, we hope that the…” to a more formal academic expression: “The release of this dataset is expected to facilitate further advances in Bangla sentiment analysis and provide a scalable foundation for multilingual and cross-lingual NLP research.”

The revised text has been highlighted in red.

________________________________________

Point 2:

I have discovered some punctuation errors. Could you please proofread the paper to make sure that there is no grammar mistake.

Author Response:

Thank you for your constructive comment. We appreciate the reviewer’s careful observation. A thorough proofreading of the entire manuscript has been conducted to identify and correct punctuation, grammatical, and typographical errors. Several sentences have been revised to improve clarity, academic tone, and grammatical correctness.

Author Action:

The full manuscript has been carefully proofread, and punctuation and grammatical issues throughout the paper have been corrected. All revised text has been highlighted in red.

________________________________________

Point 3:

The author has used many times (we, our). This is not preferred in academic writing.

Author Response:

Thank you for this important comment. We fully agree that excessive use of first-person pronouns such as “we” and “our” is not preferred in formal academic writing. To address this concern, the manuscript has been thoroughly revised to replace first-person expressions with objective and impersonal academic phrasing.

Author Action:

A full manuscript-wide revision has been performed using search-based correction (CTRL+F), and expressions such as “we”, “our”, and similar first-person forms have been replaced with formal academic alternatives such as “this study”, “the proposed system”, “the dataset”, and passive constructions.

All updated sections have been highlighted in red.

________________________________________

Point 4:

Commas after the symbol in equation 1 not after (1), this is not correct, the correct should , (1)

Author Response:

Thank you for pointing out this formatting issue. We agree that the punctuation placement around Equation (1) was not correctly formatted in the earlier version of the manuscript.

Author Action:

The equation reference formatting has been corrected throughout the manuscript. Specifically, the incorrect form “Equation, 1” has been revised to “Equation (1)”, and punctuation placement around equations has been updated according to academic formatting standards. Similar corrections have also been applied consistently to other equations in the manuscript.

All changes have been highlighted in red.

________________________________________

Point 5:

The evaluation focuses primarily on accuracy (and limited metrics). Accuracy alone may be misleading, especially under class imbalance. I want you to just give an expectation what would be the results if you considered precision, recall, F1-score. Is everything will be fine just in one sentence if possible.

Author Response:

Thank you very much for this valuable suggestion. We fully agree that accuracy alone may be insufficient for evaluating classification performance, especially in the presence of class imbalance. To address this concern, additional evaluation metrics including precision, recall, and F1-score have been explicitly emphasized in the Results and Discussion section.

Author Action:

A clarifying sentence has been added in the Results section to indicate that the comparable precision, recall, and F1-score values confirm balanced performance across all sentiment classes and support the reliability of the proposed models under class imbalance conditions.

The newly added sentence is highlighted in red.

Attachment Submitted filename: Author_Response__Plos_One_auresp_2.docx

# Peer review: Decision Letter 2

**DOI:** 10.1371/journal.pone.0360952.r005

**Authors:** Sadiq Abdulhussain

26 Jul 2026

Dear Dr. Biswas,

Thank you for submitting your manuscript to PLOS ONE. After careful consideration, we feel that it has merit but does not fully meet PLOS ONE’s publication criteria as it currently stands. Therefore, we invite you to submit a revised version of the manuscript that addresses the points raised during the review process.

Please submit your revised manuscript by Sep 09 2026 11:59PM. If you will need more time than this to complete your revisions, please reply to this message or contact the journal office at plosone@plos.org. When you're ready to submit your revision, log on to https://www.editorialmanager.com/pone/ and select the 'Submissions Needing Revision' folder to locate your manuscript file.

- A letter that responds to each point raised by the academic editor and reviewer(s). You should upload this letter as a separate file labeled 'Response to Reviewers'.
- A marked-up copy of your manuscript that highlights changes made to the original version. You should upload this as a separate file labeled 'Revised Manuscript with Track Changes'.
- An unmarked version of your revised paper without tracked changes. You should upload this as a separate file labeled 'Manuscript'.

If you would like to make changes to your financial disclosure, please include your updated statement in your cover letter. Guidelines for resubmitting your figure files are available below the reviewer comments at the end of this letter.

If applicable, we recommend that you deposit your laboratory protocols in protocols.io to enhance the reproducibility of your results. Protocols.io assigns your protocol its own identifier (DOI) so that it can be cited independently in the future. For instructions see: https://journals.plos.org/plosone/s/submission-guidelines#loc-laboratory-protocols. Additionally, PLOS ONE offers an option for publishing peer-reviewed Lab Protocol articles, which describe protocols hosted on protocols.io. Read more information on sharing protocols at https://plos.org/protocols?utm_medium=editorial-email&utm_source=authorletters&utm_campaign=protocols.

As the corresponding author, your ORCID iD is verified in the submission system and will appear in the published article. PLOS supports the use of ORCID, and we encourage all coauthors to register for an ORCID iD and use it as well. Please encourage your coauthors to verify their ORCID iD within the submission system before final acceptance, as unverified ORCID iDs will not appear in the published article. *Only*  the individual author can complete the verification step; PLOS staff *cannot*  verify ORCID iDs on behalf of authors.

We look forward to receiving your revised manuscript.

Kind regards,

Sadiq H. Abdulhussain, Ph.D.

Academic Editor

PLOS One

Journal Requirements:

If the reviewer comments include a recommendation to cite specific previously published works, please review and evaluate these publications to determine whether they are relevant and should be cited. There is no requirement to cite these works unless the editor has indicated otherwise.

Please review your reference list to ensure that it is complete and correct. If you have cited papers that have been retracted, please include the rationale for doing so in the manuscript text, or remove these references and replace them with relevant current references. Any changes to the reference list should be mentioned in the rebuttal letter that accompanies your revised manuscript. If you need to cite a retracted article, indicate the article’s retracted status in the References list and also include a citation and full reference for the retraction notice.

[Note: HTML markup is below. Please do not edit.]

Reviewers' comments:

Reviewer's Responses to Questions

**Comments to the Author**

Reviewer #3: All comments have been addressed

Reviewer #4: All comments have been addressed

**********

2. Is the manuscript technically sound, and do the data support the conclusions?

Reviewer #3: Yes

Reviewer #4: Yes

**********

3. Has the statistical analysis been performed appropriately and rigorously?

Reviewer #3: Yes

Reviewer #4: Yes

**********

4. Have the authors made all data underlying the findings in their manuscript fully available?

The PLOS Data policy

Reviewer #3: Yes

Reviewer #4: Yes

**********

5. Is the manuscript presented in an intelligible fashion and written in standard English?

Reviewer #3: Yes

Reviewer #4: Yes

**********

Reviewer #3: The manuscript is in good shape and the authors have responded carefully to the comments of the previous reviewers. Just a few minor text corrections.

Minor Matters

1- The class counts in the Data Balancing subsection (lines 393-394: negative 4,139; neutral ~2,826; positive 2,189) do not match those in Table 6 (negative 4,142; positive 2,829; neutral 2,192) and the neutral/positive numbers appear to be reversed. Make these numbers consistent with Table 6.

2- Line 371 says that the expanded short-form expressions are "shown in Figure 3", but Figure 3 is the Zipf's Law word-frequency plot. Please fix this cross-reference.

3- Some sentences lost their subjects in the revision to impersonal style, e.g. line 371 ("In this study, have expanded...") and line 772 ("In this study, introduced several advanced technologies..."). Please put these back into grammatical shape.

4- In the caption of Table 15, it is "GPT2_BERT" whereas in the text and table rows, it is "GPT2_Bengali". Standardise nomenclature.

5- Line 878 says "Class 2 (Positive)" but Table 5 shows the label scheme as 0 = Negative, 1 = Positive, 2 = Neutral. Please correct the class label in the GPT2-Bengali discussion.

Reviewer #4: In this paper, the authors present a balanced three-class Bangla sentiment analysis dataset collected from social media and annotated by native speakers. They apply a comprehensive preprocessing pipeline and evaluate multiple machine learning, deep learning, and transformer-based models.

The Authors have addressed the raised comments.

**********

what does this mean?). If published, this will include your full peer review and any attached files.

If you choose “no”, your identity will remain anonymous but your review may still be made public.

**Do you want your identity to be public for this peer review?**  For information about this choice, including consent withdrawal, please see our Privacy Policy

Reviewer #3: No

Reviewer #4: No

**********

[NOTE: If reviewer comments were submitted as an attachment file, they will be attached to this email and accessible via the submission site. Please log into your account, locate the manuscript record, and check for the action link "View Attachments". If this link does not appear, there are no attachment files.]

To ensure your figures meet our technical requirements, please review our figure guidelines: https://journals.plos.org/plosone/s/figures

You may also use PLOS’s free figure tool, NAAS, to help you prepare publication quality figures: https://journals.plos.org/plosone/s/figures#loc-tools-for-figure-preparation.

NAAS will assess whether your figures meet our technical requirements by comparing each figure against our figure specifications.

# Author comment: Author response to Decision Letter 3

**DOI:** 10.1371/journal.pone.0360952.r006

31 Jul 2026

Authors' Response to Editor and Reviewers

Title: “Sentiment Analysis in Bangla: A Three-Class Dataset and Comparative

Evaluation of Machine Learning, Deep Learning, and Transformer Approaches”

Submission ID: PONE-D-25-50471

Response to Reviewer 3: (Color code = Red)

Point 1:

The class counts in the Data Balancing subsection (lines 393-394: negative 4,139; neutral ~2,826; positive 2,189) do not match those in Table 6 (negative 4,142; positive 2,829; neutral 2,192) and the neutral/positive numbers appear to be reversed. Make these numbers consistent with Table 6.

Author Response:

Thank you for identifying this inconsistency. We have corrected the class counts in the Data Balancing subsection and ensured that the class labels and corresponding values are consistent with those reported in Table 6.

Author Action:

The Table 6 has been revised to: negative = 4,139, positive = 2,189, and neutral = 2,826. The neutral and positive class values have also been placed under the correct labels.

The revised text has been highlighted in red.

Point 2:

Line 371 says that the expanded short-form expressions are "shown in Figure 3", but Figure 3 is the Zipf's Law word-frequency plot. Please fix this cross-reference.

Author Response:

Thank you for identifying this incorrect cross-reference. We have revised the statement to remove the erroneous reference to Figure 3 and accurately describe the expansion of short-form expressions in Figure 4 during the text-preprocessing stage.

Author Action:

The incorrect cross-reference in line 371 has been updated as Figure 4. The revised text now states that the short-form expressions were expanded during data preprocessing, while Figure 3 remains correctly identified as the Zipf’s Law word-frequency plot.

Point 3:

Some sentences lost their subjects in the revision to impersonal style, e.g. line 371 ("In this study, have expanded...") and line 772 ("In this study, introduced several advanced technologies..."). Please put these back into grammatical shape.

Author Response:

Thank you for highlighting these grammatical errors. We have restored the missing subjects in the identified sentences and reviewed the revised manuscript for similar issues to ensure grammatical clarity and consistency.

Author Action:

The sentences have been corrected as follows:

Line 371: “In this study, we have expanded the short-form expressions...”

Line 772: “In this study, we introduced several advanced technologies...”

Similar subject-related grammatical errors identified elsewhere in the manuscript were also corrected.

Point 4:

In the caption of Table 15, it is "GPT2_BERT" whereas in the text and table rows, it is "GPT2_Bengali". Standardise nomenclature.

Author Response:

We appreciate the reviewer for drawing our attention to this discrepancy. We have standardized the model nomenclature throughout the manuscript to ensure that the same term is used consistently.

Author Action:

The term “GPT2_BENGALI” in the caption of Table 15 has been replaced with “GPT2_Bengali,” consistent with the terminology used in the main text and table rows. The manuscript was also checked for other inconsistent references to this model.

Point 5:

Line 878 says "Class 2 (Positive)" but Table 5 shows the label scheme as 0 = Negative, 1 = Positive, 2 = Neutral. Please correct the class label in the GPT2-Bengali discussion.

Author Response:

We appreciate the reviewer’s careful observation regarding the class-label assignment. According to the classification scheme presented in Table 5, Class 2 corresponds to the Neutral sentiment. The incorrect label has therefore been corrected.

Author Action:

The typographical error has been corrected. In the GPT2-Bengali discussion (Line 878), "Class 2 (Positive)" has been revised to "Class Positive" to maintain consistency with the class-label scheme presented in Table 5.

Attachment Submitted filename: response.docx

# Peer review: Decision Letter 3

**DOI:** 10.1371/journal.pone.0360952.r007

**Authors:** Sadiq Abdulhussain

24 Sep 2026

PONE-D-25-50471R3

Sentiment Analysis in Bangla: A Three-Class Dataset and Comparative Evaluation of Machine Learning, Deep Learning, and Transformer Approaches

PLOS One

Dear Dr. Biswas,

Thank you for submitting your manuscript to PLOS One. After careful consideration, we feel that it has merit but does not fully meet PLOS One’s publication criteria as it currently stands. Therefore, we invite you to submit a revised version of the manuscript that addresses the points raised during the review process.

Please submit your revised manuscript by Nov 23 2026 11:59PM. If you will need more time than this to complete your revisions, please reply to this message or contact the journal office at plosone@plos.org. When you're ready to submit your revision, log on to https://www.editorialmanager.com/pone/ and select the 'Submissions Needing Revision' folder to locate your manuscript file.

Please include the following items when submitting your revised manuscript:

- A letter that responds to each point raised by the academic editor and reviewer(s). You should upload this letter as a separate file labeled 'Response to Reviewers'.
- A marked-up copy of your manuscript that highlights changes made to the original version. You should upload this as a separate file labeled 'Revised Manuscript with Track Changes'.
- An unmarked version of your revised paper without tracked changes. You should upload this as a separate file labeled 'Manuscript'.

If you would like to make changes to your financial disclosure, please include your updated statement in your cover letter. Guidelines for resubmitting your figure files are available below the reviewer comments at the end of this letter.

If applicable, we recommend that you deposit your laboratory protocols in protocols.io to enhance the reproducibility of your results. Protocols.io assigns your protocol its own identifier (DOI) so that it can be cited independently in the future. For instructions see: https://journals.plos.org/plosone/s/submission-guidelines#loc-laboratory-protocols. Additionally, PLOS One offers an option for publishing peer-reviewed Lab Protocol articles, which describe protocols hosted on protocols.io. Read more information on sharing protocols at https://plos.org/protocols?utm_medium=editorial-email&utm_source=authorletters&utm_campaign=protocols.

As the corresponding author, your ORCID iD is verified in the submission system and will appear in the published article. PLOS supports the use of ORCID, and we encourage all coauthors to register for an ORCID iD and use it as well. Please encourage your coauthors to verify their ORCID iD within the submission system before final acceptance, as unverified ORCID iDs will not appear in the published article. Only the individual author can complete the verification step; PLOS staff cannot verify ORCID iDs on behalf of authors.

We look forward to receiving your revised manuscript.

Kind regards,

Sadiq H. Abdulhussain, Ph.D.

Academic Editor

PLOS One

Journal Requirements:

If the reviewer comments include a recommendation to cite specific previously published works, please review and evaluate these publications to determine whether they are relevant and should be cited. There is no requirement to cite these works unless the editor has indicated otherwise.

Please review your reference list to ensure that it is complete and correct. If you have cited papers that have been retracted, please include the rationale for doing so in the manuscript text, or remove these references and replace them with relevant current references. Any changes to the reference list should be mentioned in the rebuttal letter that accompanies your revised manuscript. If you need to cite a retracted article, indicate the article’s retracted status in the References list and also include a citation and full reference for the retraction notice.

Additional Editor Comments:

Four out of five previous comments have been adequately addressed (the Figure 4 cross-reference, restored sentence subjects at lines 371 and 772, standardized GPT2_BENGALI nomenclature, and the corrected class label in the GPT2 discussion).

Comment 1 is still open. Table 6 values have been changed to Negative 4139, Positive 2189, Neutral 2826, Total 9154, but values in the rest of the manuscript have not been changed accordingly: line 263 and lines 333–336 still have a total of 9163, lines 335–336 still have 2829 and 2192 for the positive and neutral classes (which also still appear in the reversed order), Table 16 has the size of the dataset for the proposed system as 9163, and line 897 has 9,163. The result is that the paragraph introducing Table 6 no longer corresponds to the contents of Table 6. The authors should check the class counts against the deposited Mendeley dataset (doi: 10.17632/rh67mckhbh.2) and propagate the confirmed values consistently to the Data Description, the paragraph before Table 6, Table 16, and the Discussion.

[Note: HTML markup is below. Please do not edit.]

Reviewers' comments:

Reviewer's Responses to Questions

**Comments to the Author**

Reviewer #3: All comments have been addressed

**********

2. Is the manuscript technically sound, and do the data support the conclusions?

Reviewer #3: Yes

**********

3. Has the statistical analysis been performed appropriately and rigorously?

Reviewer #3: Yes

**********

4. Have the authors made all data underlying the findings in their manuscript fully available?

The PLOS Data policy

Reviewer #3: Yes

**********

5. Is the manuscript presented in an intelligible fashion and written in standard English?

Reviewer #3: Yes

**********

Reviewer #3: Four out of five of my previous comments have been adequately addressed (the Figure 4 cross-reference, restored sentence subjects at lines 371 and 772, standardised GPT2_BENGALI nomenclature, and the corrected class label in the GPT2 discussion).

Comment 1 is still open. Table 6 values have been changed to Negative 4139, Positive 2189, Neutral 2826, Total 9154, but values in the rest of the manuscript have not been changed accordingly: line 263 and lines 333–336 still have a total of 9163, lines 335–336 still have 2829 and 2192 for the positive and neutral classes (which also still appear in the reversed order), Table 16 has the size of the dataset for the proposed system as 9163, and line 897 has 9,163. The result is that the paragraph introducing Table 6 no longer corresponds to the contents of Table 6. The authors should check the class counts against the deposited Mendeley dataset (doi: 10.17632/rh67mckhbh.2) and propagate the confirmed values consistently to the Data Description, the paragraph before Table 6, Table 16 and the Discussion.

**********

what does this mean?). If published, this will include your full peer review and any attached files.

If you choose “no”, your identity will remain anonymous but your review may still be made public.

**Do you want your identity to be public for this peer review?**  For information about this choice, including consent withdrawal, please see our Privacy Policy

Reviewer #3: No

**********

[NOTE: If reviewer comments were submitted as an attachment file, they will be attached to this email and accessible via the submission site. Please log into your account, locate the manuscript record, and check for the action link "View Attachments". If this link does not appear, there are no attachment files.]

To ensure your figures meet our technical requirements, please review our figure guidelines: https://journals.plos.org/plosone/s/figures

You may also use PLOS’s free figure tool, NAAS, to help you prepare publication quality figures: https://journals.plos.org/plosone/s/figures#loc-tools-for-figure-preparation.

NAAS will assess whether your figures meet our technical requirements by comparing each figure against our figure specifications.

# Author comment: Author response to Decision Letter 4

**DOI:** 10.1371/journal.pone.0360952.r008

25 Sep 2026

Point 1: (The Reviewer 3 and Editor provided the same comment)

Four out of five previous comments have been adequately addressed (the Figure 4 cross-reference, restored sentence subjects at lines 371 and 772, standardized GPT2_BENGALI nomenclature, and the corrected class label in the GPT2 discussion).

Comment 1 is still open. Table 6 values have been changed to Negative 4139, Positive 2189, Neutral 2826, Total 9154, but values in the rest of the manuscript have not been changed accordingly: line 263 and lines 333–336 still have a total of 9163, lines 335–336 still have 2829 and 2192 for the positive and neutral classes (which also still appear in the reversed order), Table 16 has the size of the dataset for the proposed system as 9163, and line 897 has 9,163. The result is that the paragraph introducing Table 6 no longer corresponds to the contents of Table 6. The authors should check the class counts against the deposited Mendeley dataset (doi: 10.17632/rh67mckhbh.2) and propagate the confirmed values consistently to the Data Description, the paragraph before Table 6, Table 16, and the Discussion.

Author Response:

We sincerely thank the Reviewer and Editor. We carefully rechecked the dataset statistics and clarified the distinction between the initially collected data and the final processed dataset. Initially, 9,162 comments were collected, including 8 unlabeled comments. During preprocessing, these 8 unlabeled comments were removed, resulting in a final labeled dataset of 9,154 comments. The final dataset consists of 4,139 Negative, 2,189 Positive, and 2,826 Neutral comments. We have ensured that these final dataset counts are now reported consistently throughout the manuscript.

Author Action:

We have revised the Data Description, the paragraph preceding Table 6, Table 16, and the relevant statements in the Discussion section. The paragraph introducing Table 6 has also been updated to clearly distinguish the initially collected 9,162 comments, including 8 unlabeled comments, from the final processed dataset of 9,154 labeled comments. Table 6 now consistently reports Negative = 4,139, Positive = 2,189, Neutral = 2,826, and Total = 9,154. The corresponding dataset size and class counts have also been corrected consistently throughout the manuscript.

The revised text has been highlighted in red.

Attachment Submitted filename: Author_Response__Plos_One_auresp_4.docx

# Peer review: Decision Letter 4

**DOI:** 10.1371/journal.pone.0360952.r009

**Authors:** Sadiq Abdulhussain

4 Oct 2026

Sentiment Analysis in Bangla: A Three-Class Dataset and Comparative Evaluation of Machine Learning, Deep Learning, and Transformer Approaches

PONE-D-25-50471R4

Dear Dr. Biswas,

We’re pleased to inform you that your manuscript has been judged scientifically suitable for publication and will be formally accepted for publication once it meets all outstanding technical requirements.

Within one week, you’ll receive an e-mail detailing the required amendments. When these have been addressed, you’ll receive a formal acceptance letter and your manuscript will be scheduled for publication.

An invoice will be generated when your article is formally accepted. Please note, if your institution has a publishing partnership with PLOS and your article meets the relevant criteria, all or part of your publication costs will be covered. Please make sure your user information is up-to-date by logging into Editorial Manager at Editorial Manager® and clicking the ‘Update My Information' link at the top of the page. For questions related to billing, please contact billing support.

If your institution or institutions have a press office, please notify them about your upcoming paper to help maximize its impact. If they’ll be preparing press materials, please inform our press team as soon as possible -- no later than 48 hours after receiving the formal acceptance. Your manuscript will remain under strict press embargo until 2 pm Eastern Time on the date of publication. For more information, please contact onepress@plos.org.

Kind regards,

Sadiq H. Abdulhussain, Ph.D.

Academic Editor

PLOS One

Additional Editor Comments (optional):

Reviewers' comments:

Reviewer's Responses to Questions

**Comments to the Author**

Reviewer #3: All comments have been addressed

Reviewer #5: (No Response)

**********

2. Is the manuscript technically sound, and do the data support the conclusions?

Reviewer #3: Yes

Reviewer #5: (No Response)

**********

3. Has the statistical analysis been performed appropriately and rigorously?

Reviewer #3: Yes

Reviewer #5: (No Response)

**********

4. Have the authors made all data underlying the findings in their manuscript fully available?

The PLOS Data policy

Reviewer #3: Yes

Reviewer #5: (No Response)

**********

5. Is the manuscript presented in an intelligible fashion and written in standard English?

Reviewer #3: Yes

Reviewer #5: (No Response)

**********

Reviewer #3: Assessment of the revision

The authors have adequately addressed this comment. The manuscript now mentions that 9162 comments were collected and 8 unlabeled entries were deleted, resulting in a final dataset of 9154 comments (4139 negative, 2189 positive, 2826 neutral). The above values now correspond in the Data Description, paragraph before Table 6, Table 6, SMOTE discussion, Figure 5, Table 16 and Discussion. I would like to thank the authors for their careful work through the revision rounds.

Minor editorial suggestions (no further review necessary)

1- Line 239: consider saying that 9,162 comments were collected and 9,154 were kept, so the reader isn't confused by two totals.

2- Lines 304–305: please rephrase the sentence beginning "After processed the dataset, whereas…".

3- Line 814: remove the double period after "Table 16".

My Recommendation: Accepted

All previous comments have been addressed by the authors.

Reviewer #5: ok~~~~~~~~~~~~~~~~~~~~~~~~~~~~~~~~~~~~~~~~~~~~~~~~~~~~~~~~~~~~~~~~~~~~~~~~~~~~~~~~~~~~~~~~~~~~~~~~~~~~~~~~~~~~~

**********

what does this mean?). If published, this will include your full peer review and any attached files.

If you choose “no”, your identity will remain anonymous but your review may still be made public.

**Do you want your identity to be public for this peer review?**  For information about this choice, including consent withdrawal, please see our Privacy Policy

Reviewer #3: No

Reviewer #5: No

**********

# Editor report: Acceptance letter

**DOI:** 10.1371/journal.pone.0360952.r010

**Authors:** Sadiq Abdulhussain

PONE-D-25-50471R4

PLOS One

Dear Dr. Biswas,

I'm pleased to inform you that your manuscript has been deemed suitable for publication in PLOS One. Congratulations! Your manuscript is now being handed over to our production team.

At this stage, our production department will prepare your paper for publication. This includes ensuring the following:

* All references, tables, and figures are properly cited

* All relevant supporting information is included in the manuscript submission,

* There are no issues that prevent the paper from being properly typeset

You will receive further instructions from the production team, including instructions on how to review your proof when it is ready. Please keep in mind that we are working through a large volume of accepted articles, so please give us a few days to review your paper and let you know the next and final steps.

Lastly, if your institution or institutions have a press office, please let them know about your upcoming paper now to help maximize its impact. If they'll be preparing press materials, please inform our press team within the next 48 hours. Your manuscript will remain under strict press embargo until 2 pm Eastern Time on the date of publication. For more information, please contact onepress@plos.org.

You will receive an invoice from PLOS for your publication fee after your manuscript has reached the completed accept phase. If you receive an email requesting payment before acceptance or for any other service, this may be a phishing scheme. Learn how to identify phishing emails and protect your accounts at https://explore.plos.org/phishing.

If we can help with anything else, please email us at customercare@plos.org.

Thank you for submitting your work to PLOS One and supporting open access.

Kind regards,

PLOS One Editorial Office Staff

on behalf of

Dr. Sadiq H. Abdulhussain

Academic Editor

PLOS One
